# Mb- and FnCpf1 nucleases are active in mammalian cells: activities and PAM preferences of four wild-type Cpf1 nucleases and of their altered PAM specificity variants

**DOI:** 10.1093/nar/gky815

**Published:** 2018-09-20

**Authors:** Eszter Tóth, Bernadett C Czene, Péter I Kulcsár, Sarah L Krausz, András Tálas, Antal Nyeste, Éva Varga, Krisztina Huszár, Nóra Weinhardt, Zoltán Ligeti, Adrienn É Borsy, Elfrieda Fodor, Ervin Welker

**Affiliations:** 1 Institute of Enzymology, Research Centre for Natural Sciences of the Hungarian Academy of Sciences, Budapest, H-1117, Hungary; 2 Gene Design Kft., Szeged, H-6726, Hungary; 3 Doctoral School of Multidisciplinary Medical Science, University of Szeged, Szeged, H-6726, Hungary; 4 School of Ph.D. Studies, Semmelweis University, Budapest, H-1085, Hungary; 5 Doctoral School in Biology, Faculty of Science and Informatics, University of Szeged, Szeged, H-6726, Hungary; 6 Institute of Biochemistry, Biological Research Centre, Hungarian Academy of Sciences, Szeged, H-6726, Hungary; 7 Biospirál-2006 Kft., Szeged, H-6726, Hungary

## Abstract

Cpf1s, the RNA-guided nucleases of the class II clustered regularly interspaced short palindromic repeats system require a short motive called protospacer adjacent motif (PAM) to be present next to the targeted sequence for their activity. The TTTV PAM sequence of As- and LbCpf1 nucleases is relatively rare in the genome of higher eukaryotic organisms. Here, we show that two other Cpf1 nucleases, Fn- and MbCpf1, which have been reported to utilize a shorter, more frequently occurring PAM sequence (TTN) when tested *in vitro*, carry out efficient genome modification in mammalian cells. We found that all four Cpf1 nucleases showed similar activities and TTTV PAM preferences. Our approach also revealed that besides their activities their PAM preferences are also target dependent. To increase the number of the available targets for Fn- and MbCpf1 we generated their RVR and RR mutants with altered PAM specificity and compared them to the wild-type and analogous As- and LbCpf1 variants. The mutants gained new PAM specificities but retained their activity on targets with TTTV PAMs, redefining RR-Cpf1’s PAM-specificities as TTYV/TCCV, respectively. These variants may become versatile substitutes for wild-type Cpf1s by providing an expanded range of targets for genome engineering applications.

## INTRODUCTION

Some bacterial and archaeal clustered regularly interspaced short palindromic repeats (CRISPR)-associated nucleases (Cas) have been repurposed for genome modifications (1–4), transcription modulation (5–8) and chromosome imaging (9–12) in eukaryotic cells. The area of their application is continuously widening owing to the simplicity of reprogramming their target specificity. These nucleases harbor a guide RNA that directs the cleavage of available complementary sequences. The complementary sequence (protospacer) needs to be accompanied by a short protospacer adjacent motif (PAM) to be recognized and cleaved by these Cas nucleases (13–15). Beside the most frequently employed *Streptococcus pyogenes* Cas9 (SpCas9), several SpCas9 counterparts from other organisms [such as *Staphylococcus aureus* (Sa), *Streptococcus thermophilus* (St) and *Neisseria meningitidis* (Nm)] have been exploited (16–18); however, their versatility and activity in mammalian cells have not surpassed those of SpCas9 (16,19,20).

Recently, the newly discovered Cpf1 (CRISPR from *Prevotella* and *Francisella* 1 or Cas12a) nucleases [class II, type VA (21,22)] have also been repurposed for genome engineering in mammalian and plant cells. They offer several features distinct from Cas9 nucleases, such as (i) producing 5′ overhangs, (ii) utilization of a shorter guide RNA, (iii) recognition of T-rich PAM sequences, (iv) having a longer distance between the seed sequence and the cleavage site (20,23) and (v) processing their own crRNA (24). In the pioneering study, Zetsche *et al.* analyzed 16 Cpf1 nucleases for their PAM requirements *in vitro* exploring one target sequence. Eight nucleases showed activity that was further characterized in mammalian cells on another target (23). Their analyses revealed that these nucleases exploited thymidine-rich PAM sequences of various lengths, ranging from 2 to 5 nucleotides (23). However, they found that only two of them, As- (*Acidaminococcus sp. BV3L6*) and Lb- (*Lachnospiraceae bacterium MA2020*) Cpf1 nucleases demonstrated substantial activity in mammalian HEK293FT cells (23). Subsequent studies provided more details about their mechanism of action by determining their crystal structure (25–27), by discerning the activities of their nuclease domains (25–27) and by uncovering their RNA processing activity (28). In comparison with SpCas9, a lower off-target propensity is also suggested for these Cpf1s (29,30). As- and LbCpf1 nucleases have been successfully employed for genome engineering in human HEK293 (23,24,29–31), human U2OS (30), silkworm BmN (32), mouse N2a (19) and mouse primary neural (24) cells as well as in mouse (24,33–35), Xenopus (36), zebrafish (36), rice (37–41), tobacco (42), soybean (42) and cyanobacteria (43) species. These nucleases induce indels (short insertions or deletions) with efficiencies comparable to those of SpCas9 in HEK293 cells (23,29,30). In contrast, these Cpf1s demonstrated lower homology-directed repair- (HDR), inducing activities, than SpCas9, but comparable to those of the Cas9 counterparts’ (Sa-, St- and NmCas9) when examined in N2a cells (19). These differences may be attributable to the different repair systems exploited or to the different cells employed.

Regarding their PAM preferences, AsCpf1 showed an exclusive preference for three T nucleotides (TTTN), while LbCpf1 seems to be more permissive for a C nucleotide substitution in its TTTN PAM sequence requirement *in vitro* (23). From the co-crystal structure of AsCpf1, crRNA and target DNA, it is concluded that the three thymidine bases, but not the unrestricted N base of the PAM sequence, interact with the AsCpf1 protein, supporting the reported TTTN PAM sequence requirement (25). A later study found that a preference for the three T nucleotides is also evident in mammalian cells; however, both nucleases disfavor T at position -1 leading to a TTTV PAM requirement (31,44–46).

One of the disadvantages of Cpf1-based genome editing is the relatively rare occurrence of the three-thymidine-long PAM sequences of As- and LbCpf1s. Here we aim to test the applicability of MbCpf1 (*Moraxella bovoculi 237*) and FnCpf1 (*Francisella novicida U112*) nucleases which were reported to have shorter, two-thymidine-long PAM sequences (23) for efficient genome engineering in mammalian cells.

Another approach to extend the available target space for Cpf1 nucleases is to generate mutant variants with altered PAM specificities. Recently, it was reported that mutating the serine 542, lysine 548 and asparagine 552 amino acids to arginine-valine-arginine (RVR mutant) alters the PAM specificity of AsCpf1 to include TATV PAMs, while another mutation combination (S542R+K607R, RR mutant) results in increased activity on targets with TYCV PAMs (44). Here we generate analogous RVR and RR mutant variants of Fn- and MbCpf1 and characterize them in relation to the As- and LbCpf1 variants.

## MATERIALS AND METHODS

### Materials

Restriction enzymes and T4 ligase were purchased from Thermo Fischer Scientific. DNA oligonucleotides were acquired from Sigma-Aldrich. All DNA constructs were verified by Sanger sequencing (Microsynth AG). Plasmids were purified with GenElute HP Plasmid Miniprep kit (Sigma-Aldrich). Q5 polymerase was from New England BioLabs Inc. Dulbecco's modified Eagle's Medium, foetal bovine serum, Turbofect, penicillin and streptomycin were acquired from Thermo Fisher Scientific. The following plasmids were gifts from Feng Zhang (Addgene numbers: pY004 (pcDNA3.1-hFnCpf1) #69976 (23), pY014 (pcDNA3.1-hMbCpf1) #69986 (23), pY210 [pcDNA3.1-hAsCpf1(TYCV)] #89351 (44), pY220 ([pcDNA3.1-hAsCpf1(TATV)] #89353 (44), pY230 [pcDNA3.1-hLbCpf1(TYCV)] #89355 (44), px330-U6-Chimeric_BB-CBh-hSpCas9 #42230 (2)).

### Plasmid construction

Vectors were constructed using standard molecular biology techniques. For details see Supplementary Information. The sequences of DNA oligonucleotides used in these studies are listed in Supplementary Tables S1, S2, S3, S4 and S5.

Deposited plasmids are summarized in Supplementary Table S6.

### Cell culturing and transfection

N2a cells (Neuro-2a mouse neuroblastoma cells, ATCC – CCL-131) were grown at 37°C in a humidified atmosphere of 5% CO_2_ in high glucose Dulbecco's Modified Eagle medium (DMEM) supplemented with 10% heat inactivated fetal bovine serum, 4 mM L-glutamine (Gibco), 100 units/ml penicillin and 100 μg/ml streptomycin.

HEK-293.GFP cells were generated, maintained and transfected as described in (47).

#### Modified GFxFP assay

Cells were seeded onto 48-well plates a day before transfection at a density of 3 × 10^4^ cells/well. The next day, at around 40% confluence, cells were transfected with plasmid constructs using Turbofect reagent, briefly as follows: 250 ng total plasmid DNA (2 ng GFxFP plasmid, 124 ng crRNA and nuclease expression plasmid, and 124 ng mCherry expression plasmid to monitor the transfection efficiency) and 1 μl Turbofect were mixed in 50 μl serum-free DMEM and the mixture was incubated for 30 min at room temperature prior to adding to cells. Three parallel transfections were made from each sample. Cells were analyzed by flow cytometry 2 days post transfection.

Genomic HDR assays were carried out as described in (19).

GFP disruption assay was carried out as described in (47).

### Flow cytometry

Flow cytometry analysis was carried out on either an Attune Acoustic Focusing Cytometer (Applied Biosystems by Life Technologies) or a CytoFLEX 2000 (Life Technologies Ltd.) device. For data analysis, Attune Cytometric Software and CytExpert 1.2 software were used, respectively. In all experiments, a total of 10 000 viable single cells were acquired and were gated based on side and forward light-scatter parameters. Cells expressing GFP and mCherry from a control plasmid were used to adjust the parameters for the identification of GFP and mCherry positive cells in the samples. The GFP signal was detected using the 488 nm diode laser for excitation and either the 530/30 nm filter of the Attune Acoustic Focusing Cytometer or the 525/40 nm filter of the CytoFLEX 2000 for emission. The mCherry signal was detected using the 488 nm diode laser for excitation and a 640LP filter for emission in the case of the Attune Acoustic Focusing Cytometer, and the 638 nm diode laser for excitation and an 660/20 nm filter for emission in the case of the CytoFLEX 2000.

### Next-generation sequencing

HEK293 cells were seeded onto 48-well plates a day before transfection at a density of 2 × 10^4^ cells/well. The next day, at around 25% confluence, cells were transfected with plasmid constructs using Jetfect reagent (Biospiral-2006. Ltd.), briefly as follows: 234 ng total plasmid DNA (97 ng crRNA and mCherry expression plasmid, and 137 ng nuclease expression plasmid) and 1 μl LP-25 reagent were mixed in 50 μl serum free DMEM and the mixture was incubated for 30 min at room temperature prior adding to cells. Three parallel transfections were made from each sample. Transfection efficiency was analyzed by flow cytometry 5 days post transfection via mCherry fluorescence. Then cells were centrifuged at 1000 × *g* for 10 min and genomic DNA was purified according to Puregene DNA Purification protocol (Gentra Systems). Amplicons for deep sequencing were generated using two rounds of PCR by Q5 high fidelity polymerase to attach Illumina handles. The first step PCR primers used to amplify target genomic sequences are listed in Supplementary Table S7. After being quantified with Qubit dsDNA HS Assay kit (Invitrogen) PCR products were pooled for deep sequencing. Sequencing on Illumina Miseq instrument was performed by ATGandCo Ltd. Indels were counted computationally among reads that matched at least 75% to the first 20 bp of the reference amplicon. Indels and mismatches were searched at ±60 bp around the cut site. For each sample, the indel frequency was determined as (number of reads with an indel)/(number of total reads). Average reads per sample was 23 398 with a minimum as 8133. No sample was excluded due to fewer than 1000 total reads. The following software were used: BBMap 38.08, samtools 1.8, BioPython 1.71, PySam 0.13. SRA accession: SRP155357.

## RESULTS

### Mb- and FnCpf1 nucleases have comparable on-target activity to that of As- and LbCpf1 in mammalian cells employing a GFxFP reporter assay

**Figure 1.**
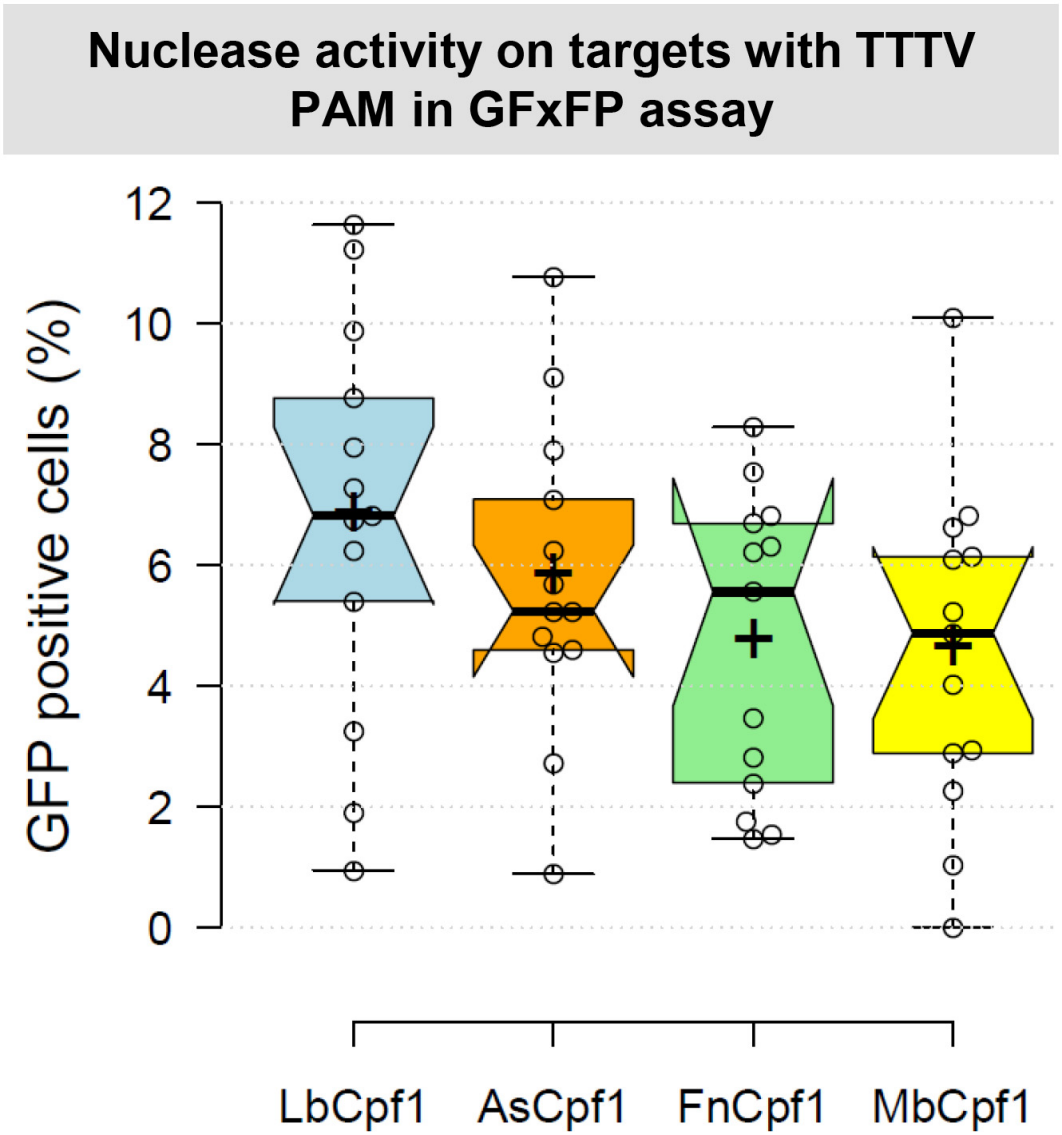
Comparison of cleavage efficiencies of different Cpf1 nucleases in GFxFP plasmid-based assay in mouse N2a cells. Percentages of GFP positive cells counted above the background level, resulting from the action of various nucleases (blue – LbCpf1, orange – AsCpf1, green – FnCpf1, yellow – MbCpf1). Thirteen randomly picked targets cloned into the pGF-ori-FP vector (19) were tested using the GFxFP assay. The target vectors along with the corresponding nuclease vector were transfected into N2a cells and GFP positive cells were counted 2 days after transfection. All samples are also cotransfected with an mCherry expression vector to monitor the transfection efficiency and the GFP signal is analyzed within the mCherry positive population. The background fluorescence was estimated by using a crRNA-less, inactive AsCpf1 nuclease expression vector as negative control and was subtracted from each sample. Three parallel transfections were made for each case. Tukey-type notched boxplots by BoxPlotR (53): center lines show the medians; box limits indicate the 25th and 75th percentiles; whiskers extend 1.5 times the interquartile range from the 25th and 75th percentiles; notches indicate the 95% confidence intervals for medians; crosses represent sample means; data points are plotted as open circles that correspond to the different targets tested. See also Supplementary Figure S3.

Since As- and LbCpf1 demonstrated higher activities when their crRNAs were expressed from a plasmid instead of from a PCR product (19), we introduced a human U6 promoter driven crRNA cassette into pY004-pcDNA3.1-hFnCpf1 and pY014-pcDNA3.1-hMbCpf1 vectors (Addgene numbers: #69976 and #69986, respectively) (23) (Supplementary Figure S1). In order to monitor the activity of Mb- and FnCpf1 nucleases we cloned thirteen different, randomly picked spacer sequences into these vectors (Supplementary Table S1). To test the cleavage efficiency of Cpf1 nucleases in mammalian cells, we employed a GFxFP reporter assay previously reported (Supplementary Figure S2 and Supplementary Table S2) (19) that is based on the recovery of an interrupted GFP (green fluorescent protein) sequence containing about 500 nucleotide-long homologous stretches. The assay was refined for this study by altering the ratios of the transfected plasmids in a way that dramatically decreased the background GFP level (below 1–2%) in N2a cells. The cleavage efficiency of the Lb-, As-, Mb- and FnCpf1 nucleases was comparable when assessed on the same targets with three-thymidine-nucleotide PAM sequence (Figure 1, Supplementary Figure S3); however, LbCpf1 seems to perform slightly better. To provide appropriate controls, we generated two inactivated RuvC nuclease domain mutants for each of the three Cpf1 nucleases (As-, Lb- and MbCpf1) based on the sequence similarity of these nucleases and the inactivating mutations identified in an earlier study (23), which in the case of FnCpf1 were the D917A and E1006A substitutions (23). Both mutations were equally effective in abolishing the activities of these Cpf1 nucleases, resulting in no detectable activity in the GFxFP assay (Supplementary Figure S4 and Supplementary Table S3) for both. These results agree with those of Yamano *et al.* (25).

On the basis of these results on the activity of Fn- and MbCpf1 employing a plasmid-based assay system, we examined their homology-directed recombination inducing activity on genomic targets.

### Homology directed recombination-inducing activity of different Cpf1 nucleases on targets with TTTN PAM sequence in mouse genomic environment

**Figure 2.**
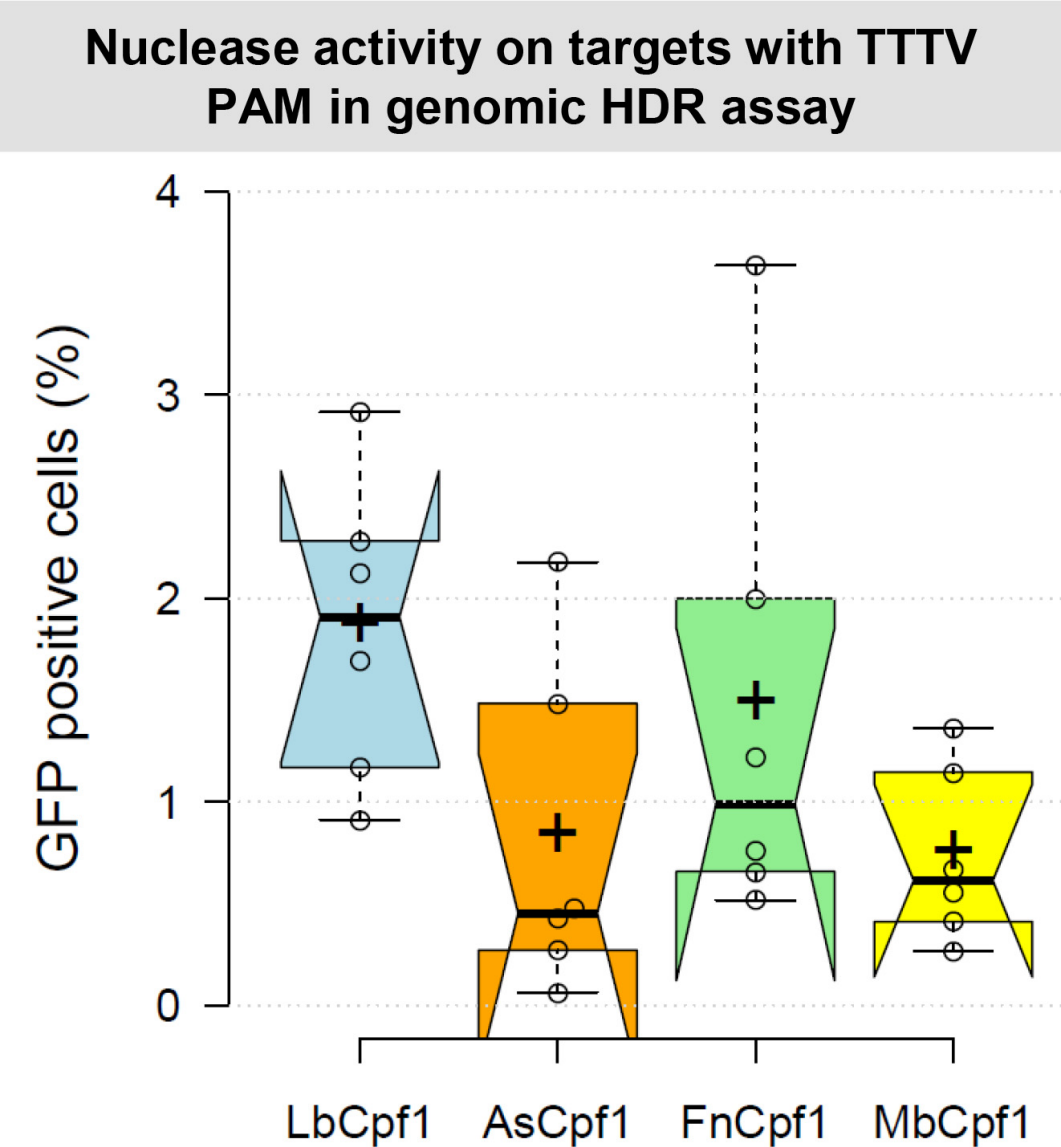
Cpf1 nucleases induced HDR at various genomic cleavage sites in N2a cells. Percentages of GFP fluorescent cells after HDR mediated integration of a donor GFP cassette are shown. The efficiencies of Cpf1 nucleases (blue – LbCpf1, orange – AsCpf1, green – FnCpf1, yellow – MbCpf1) in inducing HDR mediated integration were measured on six mouse doppel genomic targets. The nuclease vector and the homologous recombination donor molecule with the corresponding homologous arms were co-transfected into N2a cells. As negative control, the homologous recombination donor without the corresponding homologous arms was used. On the 14th day after transfection, GFP positive cells were counted. The corresponding negative control was subtracted from each sample. Three parallel transfections were made for each sample. Two days after transfection all the samples made on the same day showed similar GFP positive cell counts. This GFP fluorescence was used to normalize the results for variation in transfection efficiency. Tukey-type notched boxplots by BoxPlotR (53): center lines show the medians; box limits indicate the 25th and 75th percentiles; whiskers extend 1.5 times the interquartile range from the 25th and 75th percentiles; notches indicate the 95% confidence intervals for medians; crosses represent sample means; data points are plotted as open circles that correspond to the different targets tested. See also Supplementary Figure S6.

We examined the HDR-inducing activities of the four Cpf1 nucleases on the mouse *Prnd* gene exploiting six different target sequences (PRND 1–6). For these experiments, we generated two homologous recombination donor molecules, one with homologous arms matching the *Prnd* locus (pHRdonor-EGFP-Dpl1000) and another one with homologous arms directed to a different genomic locus (*Sprn* locus: pHRdonor-EGFP-Sho1000) to be used here as a control. Both donor molecules contain a GFP expression cassette. To see if the selected genomic target-positions are indeed available for RNA-guided nucleases, we analyzed the HDR-inducing activity of SpCas9 on overlapping positions with the Cpf1 targets (Supplementary Table S4). SpCas9 efficiently induced HDR-mediated integration of the GFP ORF to these target sites (Supplementary Figure S5) suggesting that the targeted regions are available for RNA-guided nuclease cleavage. To test the Cpf1 nucleases, the Cpf1-expressing plasmids containing a crRNA targeting the *Prnd* locus (Supplementary Table S1) were co-transfected with the donor plasmid and the number of GFP expressing cells were monitored two weeks later when the transient GFP expression from the transfected donor plasmid decreased to background level. The background fluorescence was at equally low level in case of the two control samples, where the Cpf1 nucleases were co-transfected with either a plasmid containing no corresponding homology arms (pHRdonor-EGFP-Sho1000), or where inactive nucleases were co-transfected with the donor plasmid (Supplementary Figure S6). These indicate that Cpf1 nuclease activity does not result in any significant fluorescence originating from non-homology mediated on- or off-target integrations with these targets that would exceed the random-integration background level. Figure 2 shows that all four nucleases can mediate HDR in mouse N2a cells, although to a different extent. Out of the six *Prnd* targets tested, LbCpf1 cleaved five, FnCpf1 cleaved three, AsCpf1 and MbCpf1 cleaved two targets resulting in more than 1% targeted integration above the background level (Figure 2, Supplementary Figure S6).

### NHEJ-inducing activity of different Cpf1 nucleases on targets with TTTN PAM sequence

**Figure 3.**
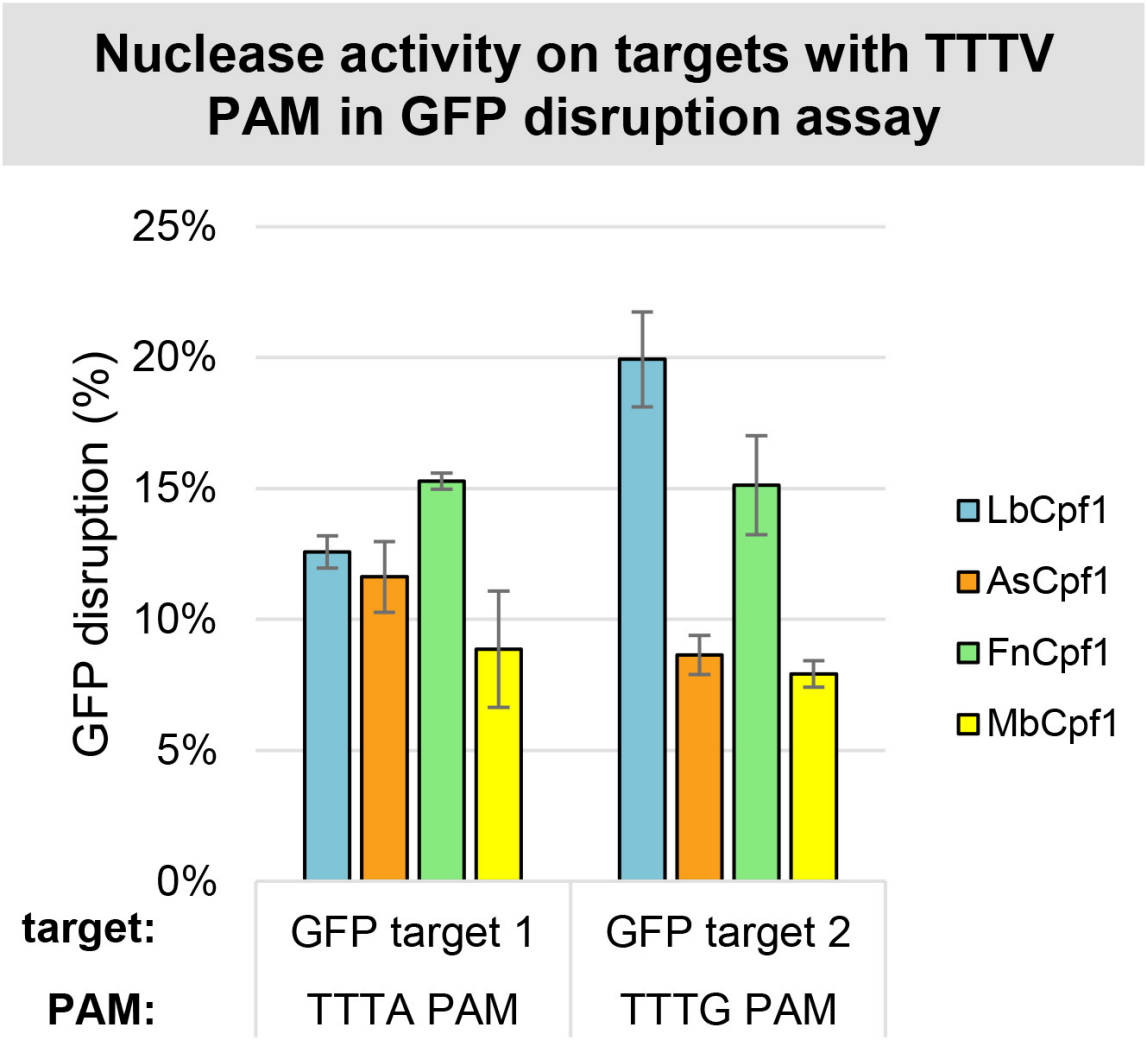
GFP disruption mediated by different Cpf1 nucleases on targets with TTTV PAMs in HEK-293.GFP cells. GFP disruption mediated by different Cpf1 nucleases (blue bars: LbCpf1, orange bars: AsCpf1, green bars: FnCpf1 and yellow bars: MbCpf1) on targets with a TTTA (GFP target 1) or a TTTG (GFP target 2) PAM sequence. The nuclease vector along with the corresponding crRNA expressing vector were transfected into HEK-293.GFP cells and GFP positive cells were counted 7 days post-transfection by flow cytometry. For controls we used either inactive Cpf1 expressing nuclease vectors or no-target crRNA expressing vectors. The average GFP % of negative controls was 95.72 ± 1.13%. GFP disruption activities of the nucleases were calculated as follows: 1 – (sample GFP %/average GFP % of negative controls). Bars correspond to averages of *n* = 3 parallel samples. The experiment was repeated six times and a representative dataset is presented here.

We examined the NHEJ-inducing activity of different Cpf1 nucleases by transfecting HEK-293.GFP cells with both a nuclease expressing plasmid and a crRNA expressing plasmid exploiting a GFP disruption assay (47). Because of the rarity of the TTTV PAM sequences, only two targets with TTTV PAM sequence (GFP target 1 and GFP target 2) are present in the GFP sequence. All four nucleases exhibited activities on both targets resulting in disruptions ranging between 4 and 22%, likely reflecting different target-dependencies (Figure 3). The results obtained here with Cpf1 nucleases seem to be lower than those of SpCas9, which reached ∼62% disruption on average in the same cells (47); however, the low number of targets tested are not sufficient to reach a definite conclusion upon their activities.

**Figure 4.**
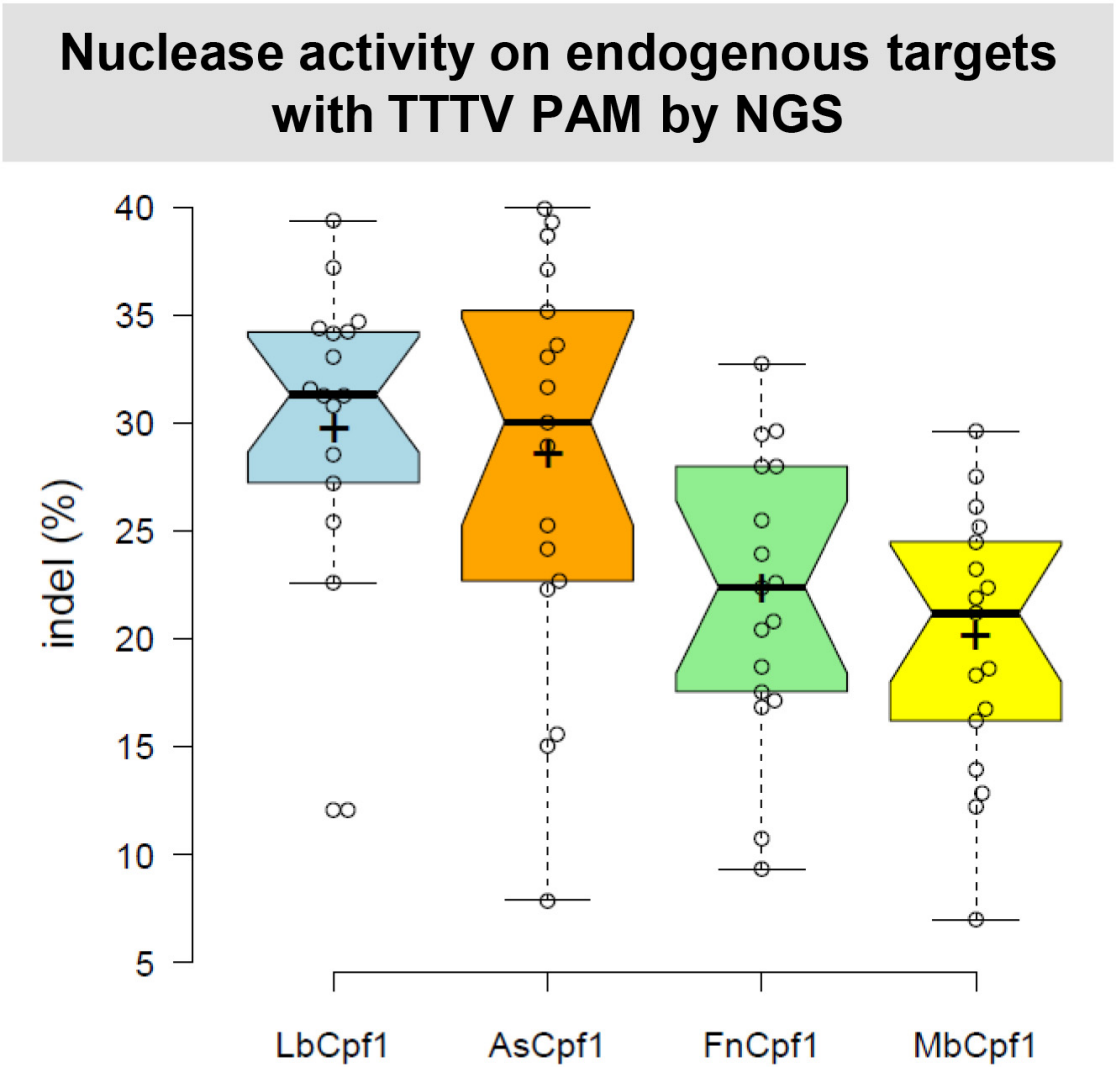
Indels mediated by different Cpf1 nucleases on endogenous targets with TTTV PAM sequence in HEK293 cells. Indels mediated by different Cpf1 nucleases (blue bars: LbCpf1, orange bars: AsCpf1, green bars: FnCpf1 and yellow bars: MbCpf1) on 17 endogenous targets with TTTV PAM sequence. The nuclease vector along with the corresponding crRNA expressing vector were transfected into HEK293 cells and indels were analyzed 5 days post-transfection by deep sequencing. Three parallel transfections were made for each sample. Tukey-type notched boxplots by BoxPlotR (53): center lines show the medians; box limits indicate the 25th and 75th percentiles; whiskers extend 1.5 times the interquartile range from the 25th and 75th percentiles; notches indicate the 95% confidence intervals for medians; crosses represent sample means; data points are plotted as open circles that correspond to the different targets tested. See also Supplementary Figures S7 and S8.

To get a more detailed characterization of the activity of these nucleases, we choose to test the indel-inducing activity of Cpf1 nucleases on 17 human endogenous genomic target sites (Supplementary Table S1) using HEK293 human cells. A nuclease expressing plasmid along with a cognate crRNA expressing plasmid was transfected into HEK293 cells and their genomic DNA was analyzed by next-generation sequencing. The four nucleases exhibited activities on all targets examined (Figure 4 and Supplementary Figures S7 and S8) in line with our former disruption results (Figure 3). However, Lb- and AsCpf1 demonstrated higher activities on these endogenous targets than Fn- and MbCpf1.

### PAM requirement of different Cpf1 nucleases

**Figure 5.**
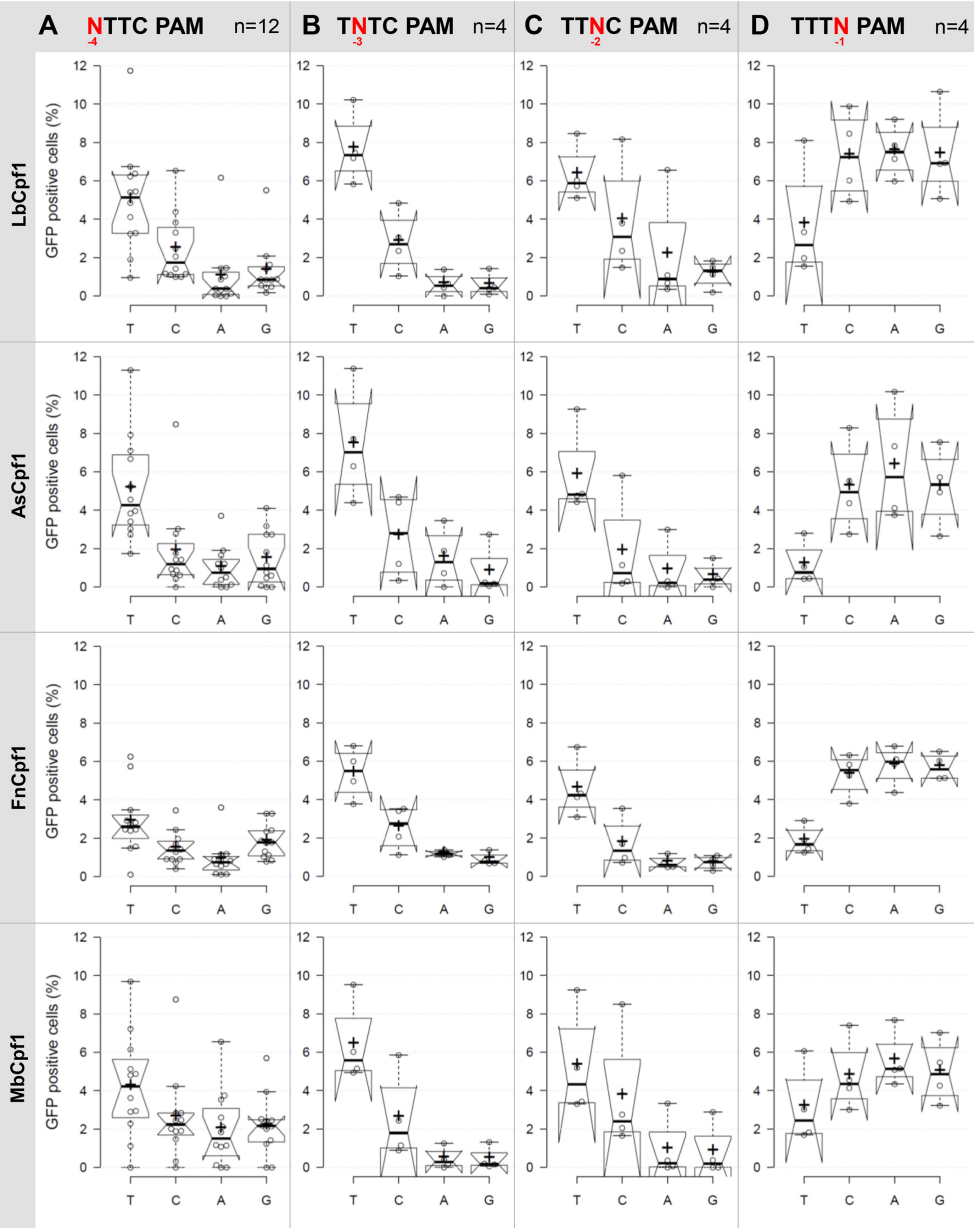
PAM requirement of Lb-, As-, Mb- and FnCpf1 nucleases. Percentages of GFP positive cells counted above the background level, resulting from the action of various nucleases on targets with different PAM sequences. Four or twelve (indicated as n) randomly picked targets with different PAM sequences (**A**) NTTC PAM sequence, (**B**) TNTC PAM sequence, (**C**) TTNC PAM sequence, (**D**) TTTN PAM sequence) cloned into the pGF-ori-FP vector were tested using the GFxFP assay (19). The target vectors along with the corresponding nuclease vector were transfected into N2a cells and GFP positive cells were counted 2 days after transfection. All samples were also cotransfected with an mCherry expression vector to monitor the transfection efficiency and the GFP signal was analyzed within the mCherry positive population. The background fluorescence was assessed by using a crRNA-less, inactive AsCpf1 nuclease expression vector as negative control and was subtracted from each sample. Three parallel transfections were made for each case. Tukey-type notched boxplots by BoxPlotR (53): center lines show the medians; box limits indicate the 25th and 75th percentiles; whiskers extend 1.5 times the interquartile range from the 25th and 75th percentiles; notches indicate the 95% confidence intervals for medians; crosses represent sample means; data points are plotted as open circles that correspond to the different targets tested. See also Supplementary Figures S9–S12.

**Figure 6.**
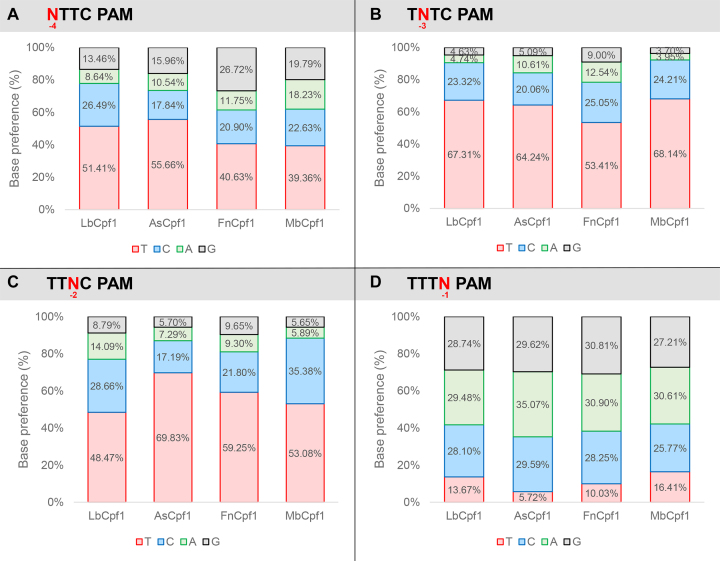
Summary of PAM requirement of Lb-, As-, Mb- and FnCpf1 nucleases. Ratios of activities of the four nucleases averaged over targets in which the nucleotides have been varied systematically at one of the positions of the TTTN PAM sequence. Base preference at (**A**) -4; (**B**) -3; (**C**) -2; (**D**) -1 PAM positions. Red: thymidine, blue: cytosine, green: adenine, gray: guanine. See also Supplementary Figures S9–S12.

Having successfully established that Fn- and MbCpf1 nucleases are active in mammalian N2a and HEK-293.GFP cells we aimed to characterize their PAM requirements by exploiting the previously employed GFxFP reporter system. The advantage of this system over testing a number of genomic targets with various PAM sequences is that the nucleotides of the PAM can be systematically varied while employing identical protospacer sequences; therefore, the effect of different targets on the cleavage activity can clearly be distinguished from the PAM preferences. We varied the four nucleotides, one at a time, at each of the four positions of the TTTC PAM sequences of four different targets (that are identical with targets 4, 5, 7 and 8 on Supplementary Figure S3) and counted the GFP positive cells at the second day post-transfection. Although we count the number of the GFP positive cells in the transfected cell population assessed by the co-transfection of an mCherry-expressing plasmid, we could not account fully for the varying transfection efficiencies. To minimize the impact of different transfection efficiencies on the results, the experiments to be compared by the four Cpf1 nucleases were carried out side by side on the same days. Figure 5 reveals the activities obtained for each nuclease with the different PAM sequences examined (Figures 5 and 6, Supplementary Figures S9–S12). A clear, but not exclusive thymidine nucleotide preference is apparent at PAM position -2 and -3 for all four nucleases (Figures 5B, C and 6B, C). These experiments also revealed that PAM position -1 is not unrestricted, since thymidine is disfavored by all four nucleases, without any further limitation on the use of the rest of the three bases (Figures 5D and 6D). Thus, the consensus sequences of the PAM requirement of As- and LbCpf1 is TTTV (V: all but T); although cytosine is also tolerated to some extent as a second option at position -3 and -2 (Figures 5 and 6 blue, Supplementary Figures S9–S12). Fn- and MbCpf1 nucleases seem to possess very similar PAM preferences (Figures 5 and 6). To be able to clearly distinguish whether Fn- and MbCpf1 really possess a more relaxed sequence requirement at position -4 in mammalian cells as was suggested by *in vitro* cleavage assays (23) and by PAM-SCANR in bacteria (48), we examined their base preferences exploring eight additional targets. Interestingly, we did not find sharp differences among the base preferences for this PAM position of the four nucleases. All four Cpf1 nucleases favor a T nucleotide at position -4, although in a lesser extent than in position -3 or -2. This preference is less pronounced for Fn- and MbCpf1 (Figure 6A, Supplementary Figures S9–S12) suggesting that As- and LbCpf1 have a more relaxed, while Fn- and MbCpf1 have a stricter sequence requirement in mammalian cells, as compared to when either under *in vitro* conditions or in bacterial system (23,48). Our approach also revealed that the Cpf1 nuclease's PAM preferences are target-dependent (Supplementary Figures S9–S12).

### Activities of different Cpf1 nucleases on targets with various VTTV PAMs

**Figure 7.**
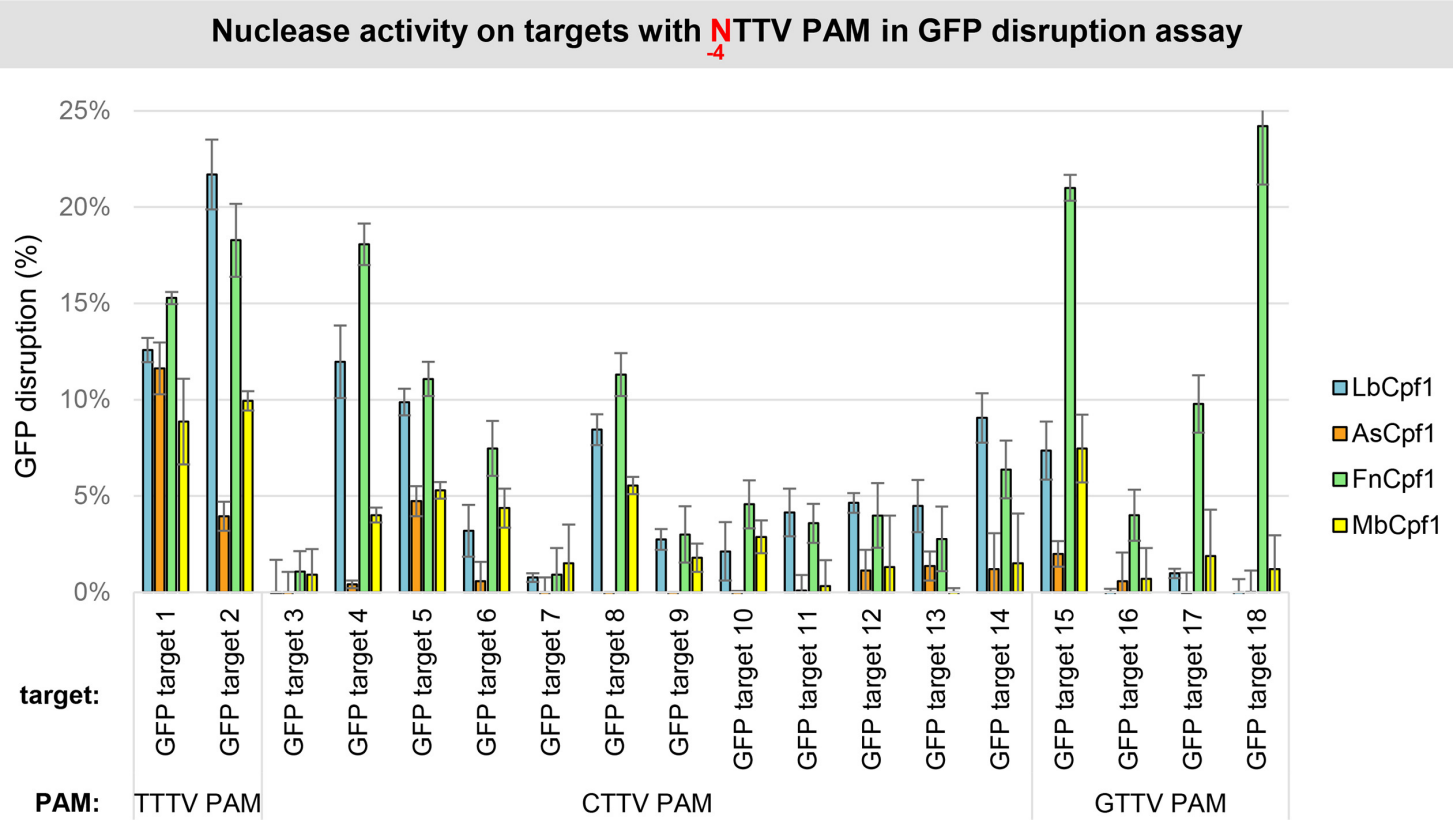
GFP disruption mediated by different Cpf1 nucleases on targets with NTTV PAMs in HEK-293.GFP cells. GFP disruption mediated by different Cpf1 nucleases (blue bars: LbCpf1, orange bars: AsCpf1, green bars: FnCpf1 and yellow bars: MbCpf1) on a range of different targets with an NTTV PAM sequence (GFP target 1–18). The nuclease vector along with the corresponding crRNA expressing vector were transfected into HEK-293.GFP cells and GFP positive cells were counted 7 days post-transfection by flow cytometry. For controls we used either inactive Cpf1 expressing nuclease vectors or no-target crRNA expressing vectors. GFP disruption activities of the nucleases were calculated as follows: the measured percentage of GFP-positive cells for each sample was subtracted from the total average obtained on the controls. Bars correspond to averages of *n* = 3 parallel samples. The experiment was repeated two times and a representative dataset is presented here.

Although we could not verify that Fn- and MbCpf1 nucleases require only two-nucleotide-long PAM sequences by employing the GFxFP assay in mouse N2a cells, we analyzed the activity of the four Cpf1 nucleases using the disruption assay [HEK-293.GFP cells, (47)] on 16 different targets with various VTTV PAMs (CTTC, CTTG, GTTC and GTTG PAMs; GFP target 3–18 on Figure 7). On half of the targets none of the four Cpf1 nucleases induce GFP disruption exceeding 5% (target 3, 7, 9–13, 16 on Figure 5). On the rest, Lb- and FnCpf1 result in higher indel-inducing activity on a target-dependent manner (Figure 7). Interestingly, FnCpf1, in contrast to LbCpf1, shows higher activity on the four targets with GTTV PAM (GFP target 15–18 on Figure 7) that is consistent with its slight preference for G in position -4 seen on Figure 5 and reported in (49).

### Engineered RVR mutants of Fn- and MbCpf1 with altered PAM specificities

**Figure 8.**
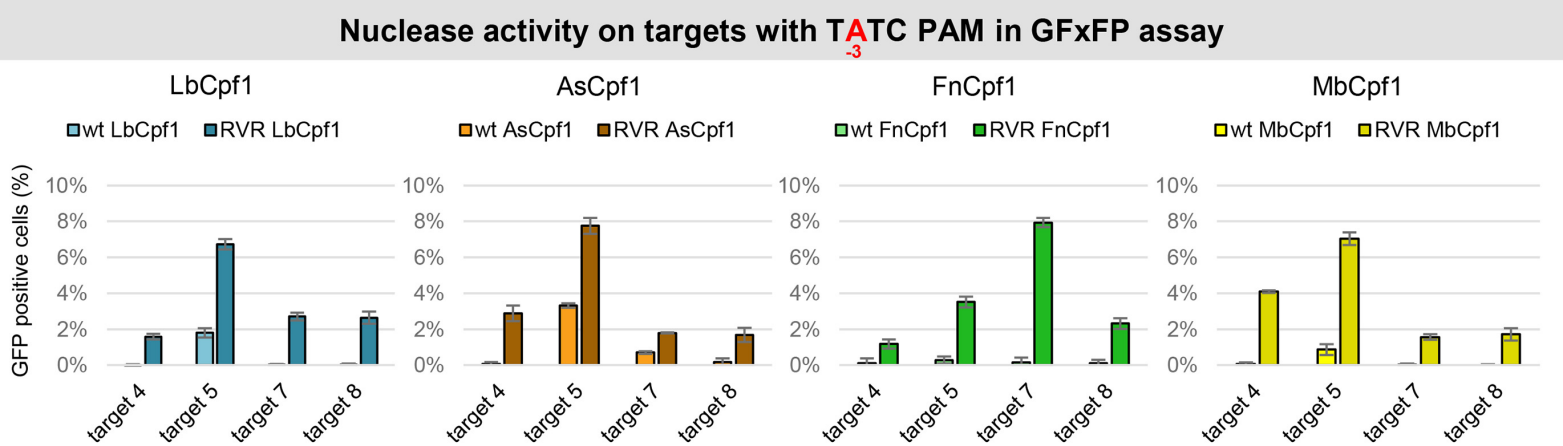
Activity of WT and RVR mutants on targets with TATC PAM sequence in N2a cells. We compared the activity of WT and RVR mutant Cpf1 nucleases on targets with TATC PAM sequences in a GFxFP assay. Percentages of GFP positive cells counted above the background level resulting from the action of As- (orange), Lb- (blue), Fn- (green) and MbCpf1 (yellow) are shown. The target vectors along with the corresponding nuclease vectors were transfected into N2a cells and GFP positive cells were counted 2 days post-transfection. All samples were also cotransfected with an mCherry expression vector to monitor the transfection efficiency and the GFP signal was analyzed within the mCherry positive population. The background fluorescence was estimated by using a crRNA-less, inactive LbCpf1 nuclease expression vector as negative control and was subtracted from each sample. Three parallel transfections were made for each case. Error bars show the mean ± standard deviation of percentages measured in *n* = 3 independent transfections. See also Supplementary Figure S14.

**Figure 9.**
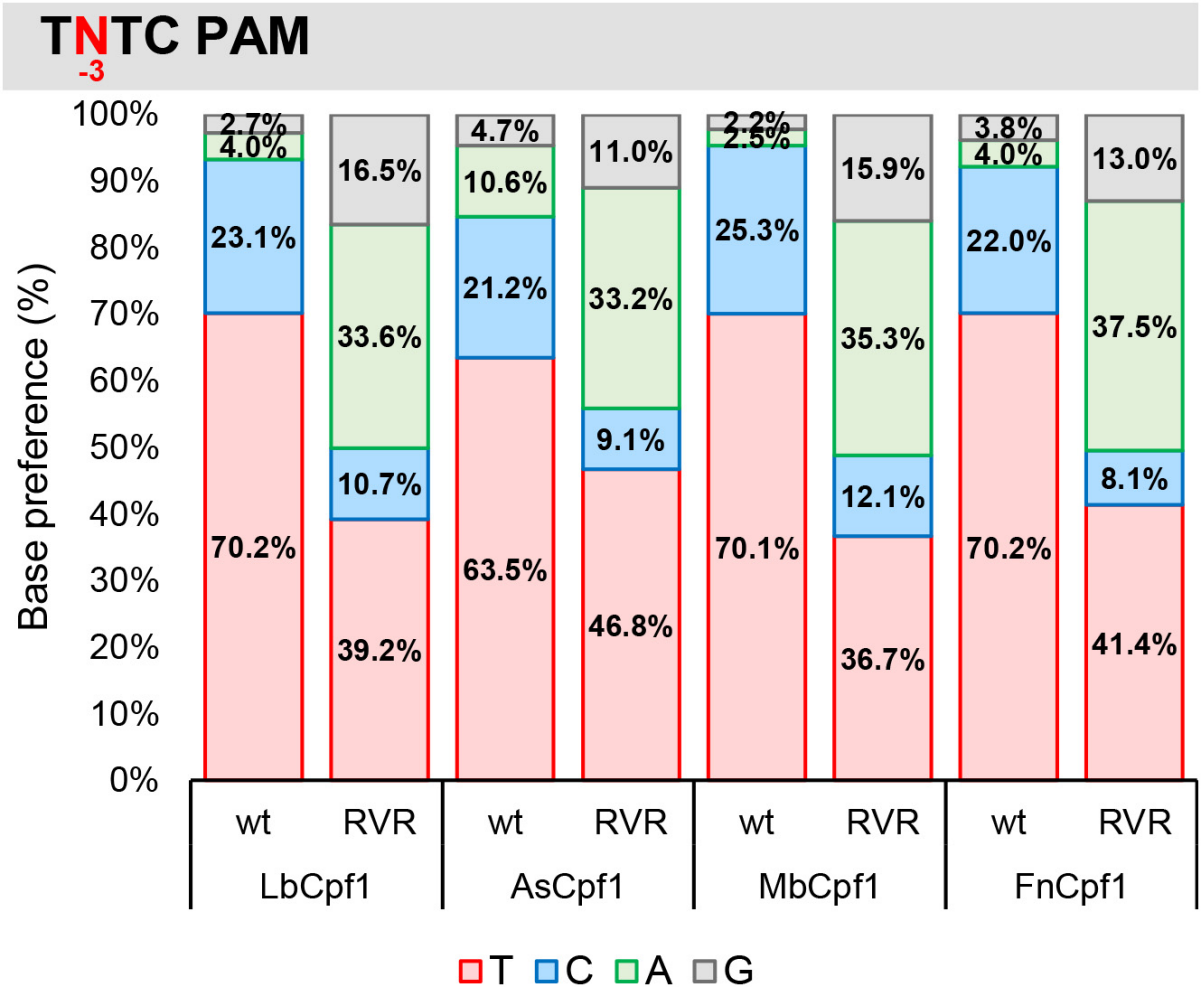
Comparison of base preference of WT and RVR mutant Cpf1s at PAM position -3. Ratios of activities of the four nucleases averaged over targets in which the nucleotides have been varied systematically at PAM position -3 (TNTC). Red: thymidine, blue: cytosine, green: adenine, gray: guanine. See also Supplementary Figure S14.

**Figure 10.**
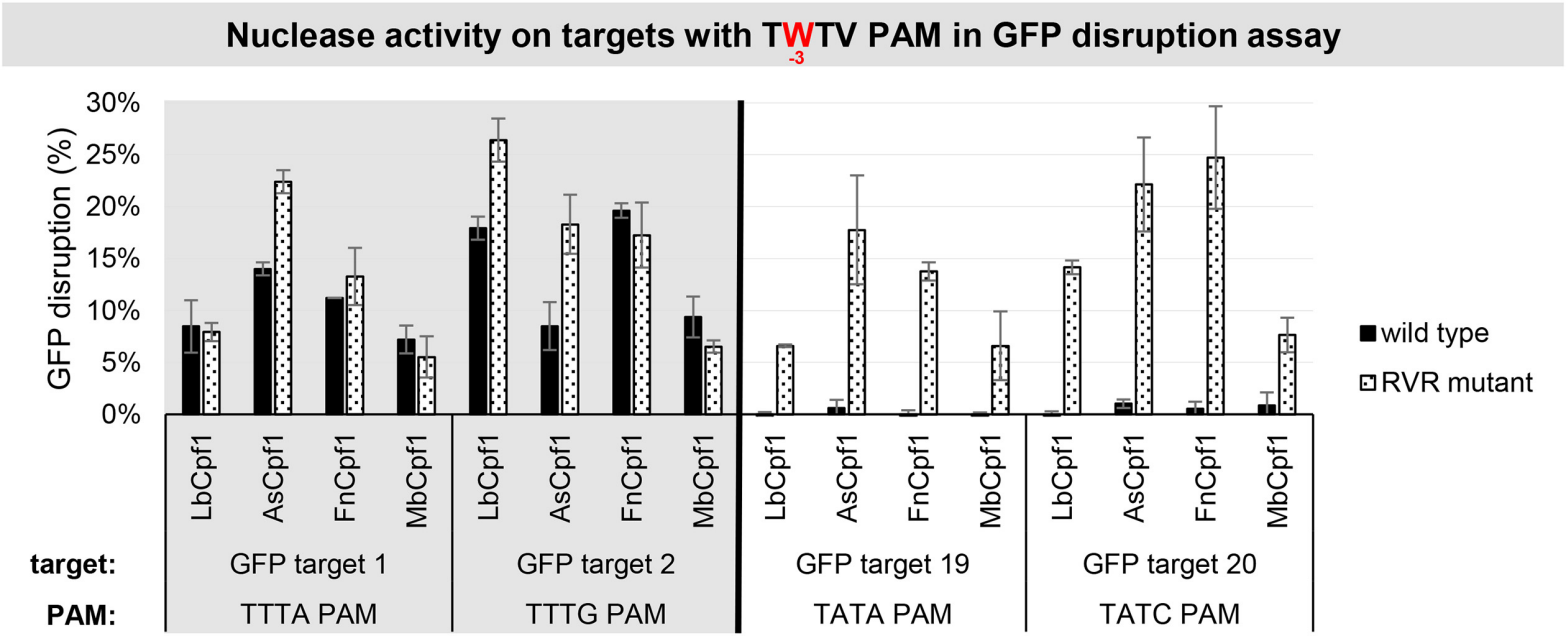
GFP disruption mediated by WT and RVR Cpf1 variants on targets with TWTV PAMs in HEK-293.GFP cells. GFP disruption mediated by WT (black) and RVR (dotted) Cpf1 variants on targets with TWTV PAM sequence (TTTV PAM: GFP target 1–2, gray background; TATV PAM: GFP target 19–20, no background). The nuclease vector along with the corresponding crRNA expressing vector were transfected into HEK-293.GFP cells and GFP positive cells were counted 7 days post-transfection by flow cytometry. For controls we used either inactive Cpf1 expressing nuclease vectors or no-target crRNA expressing vectors. GFP disruption activities of the nucleases were calculated as follows: the measured percentage of GFP-positive cells for each sample was subtracted from the total average obtained with the controls. Bars correspond to averages of *n* = 3 parallel samples.

Since neither Fn- nor MbCpf1 proved to utilize a shorter TTN PAM sequence, we turned to another approach to lessen the stringency of the PAM requirement of Cpf1 nucleases. Recently, it was reported that mutating the serine 542, lysine 548 and asparagine 552 amino acids to arginine-valine-arginine (RVR mutant) alters the PAM specificity of AsCpf1 toward TATV PAMs, therefore expanding the number of available targets of AsCpf1 (44). We proposed that the analogous mutations (FnCpf1: N607R+K613V+N617R, MbCpf1: N576R+K582V+N586R) will alter the PAM specificity of Fn- and MbCpf1 too (Supplementary Figure S13). As we found that Cpf1s may have different activity and PAM preference on different targets (Supplementary Figures S9–S12), we expected that these mutations would become a useful complement to the existing RVR mutants of As- and LbCpf1. We introduced these mutations into Fn- and MbCpf1 nucleases and analyzed their PAM requirements in comparison to the As- and LbCpf1 variants, as well as to the corresponding wild-type (WT) protein on four different targets (targets 4, 5, 7 and 8 on Supplementary Figure S3) in the GFxFP assay. The mutations altered the PAM specificities in a similar manner for all four variants affecting the -3 position (TTTV) of their PAM sequences. Compared to the WT Cpf1 nucleases all RVR mutants have an elevated activity on targets with TATC PAMs that are comparable to that of the WT Cpf1 nucleases on the same targets with the canonical TTTV PAM (Figures 8 and 9, Supplementary Figure S14). This is in accord with the results of Gao *et al.* (44). Their activity on targets with TCTC PAMs slightly decreased, while on targets with TGTC PAMs slightly increased in a nuclease- and target-dependent manner (Figure 9 and Supplementary Figure S14). The mutation had no effect on the PAM preference at position -4 (NTTV; Supplementary Figures S15 and S16). Interestingly, in these experiments, all the RVR mutants retained their activity on TTTC PAMs comparable to those of the WT proteins (Figure 9 and Supplementary Figure S14) as reported earlier for AsCpf1 (45). Since the RVR mutants recognize targets with both A or T at position -3 of the PAM sequences with comparable activities on the same targets, we suggest that a TWTV sequence (where W is A or T and V is G, C or A) better describes their PAM preferences. We verified this on four different genomic targets with either TTTV (GFP target 1, 2) or TATV (GFP target 19, 20) PAM sequences in comparison to the WT proteins in HEK-293.GFP cells (Figure 10).

### Engineered RR mutants of Fn- and MbCpf1 with altered PAM specificities

**Figure 11.**
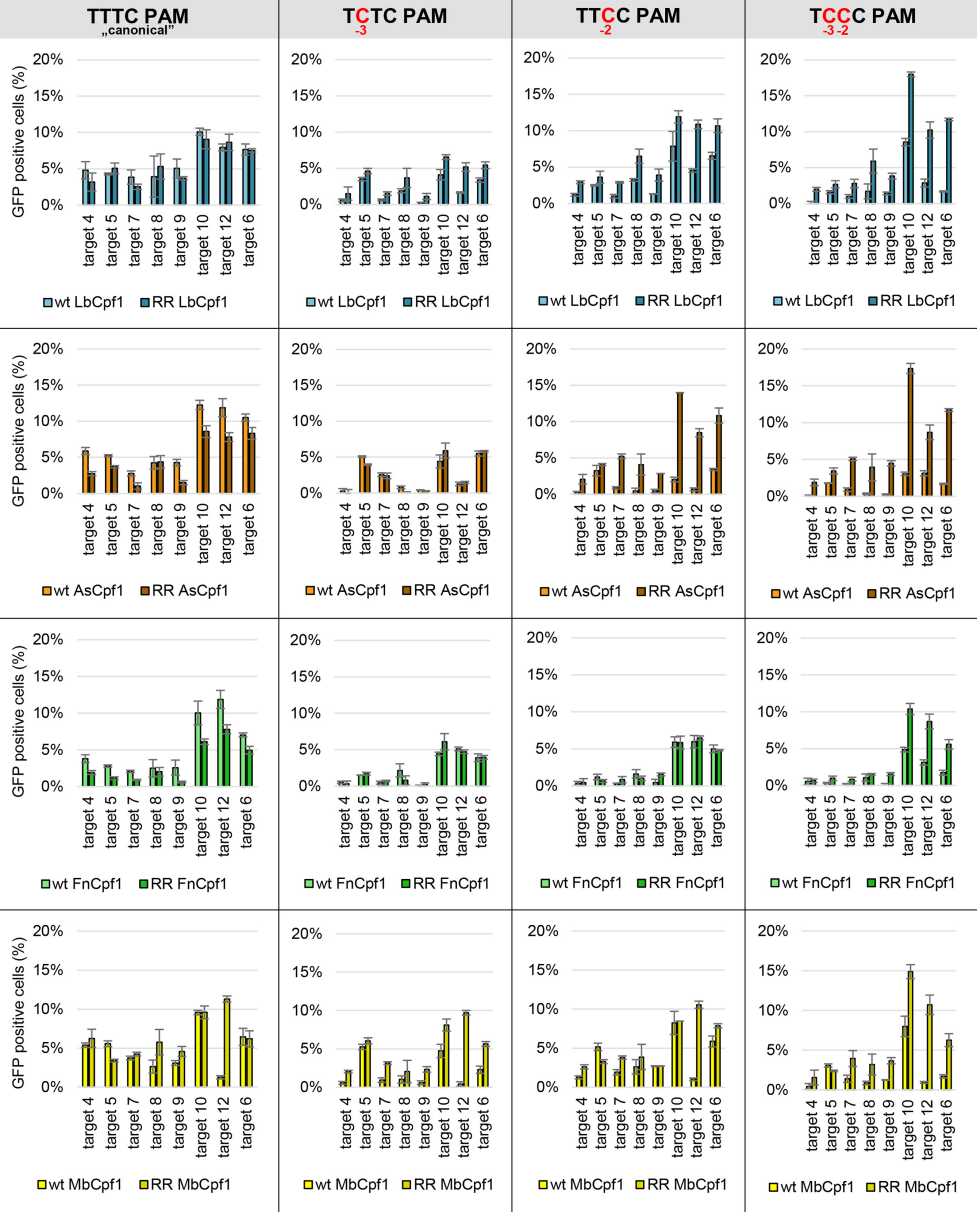
Activity of WT and RR mutants on targets with TYYC PAM sequences in N2a cells. We compared the activity of WT and RR mutant Cpf1 nucleases on targets with TYYC PAM sequences (first column: TTTC, second column: TCTC, third column: TTCC, fourth column: TCCC) in a GFxFP assay. Percentages of GFP positive cells counted above the background level resulting from the action of As- (orange), Lb- (blue), Fn- (green) and MbCpf1 (yellow) are shown. The target vectors along with the corresponding nuclease vectors were transfected into N2a cells and GFP positive cells were counted 2 days post-transfection. All samples were also cotransfected with an mCherry expression vector to monitor the transfection efficiency and the GFP signal was analyzed within the mCherry positive population. The background fluorescence was estimated by using a crRNA-less, inactive LbCpf1 nuclease expression vector as negative control and was subtracted from each sample. Three parallel transfections were made for each case. Error bars show the mean ± standard deviation of percentages measured in *n* = 3 independent transfections. See also Supplementary Figures S17–S21.

Gao *et al.* reported another mutation combination (S542R+K607R, RR mutant), which also altered the PAM preferences of AsCpf1 resulting in increased activity on targets with TYCV PAMs (44). We generated the analogous RR mutants of Fn- and MbCpf1 (Supplementary Figure S13) and compared their PAM preferences to that of As- and LbCpf1 nucleases in GFxFP assay on eight targets (target 4–10 and 12 on Supplementary Figure S3). We found that their activity toward targets with the ‘canonical’ TTTC PAMs have not changed much although for the RR mutants of As- and FnCpf1 a slight decrease can be observed (Figure 11, first column, Supplementary Figure S17). Analyzing PAM position -3 (TNTC), we confirmed that all four WT Cpf1 nucleases exhibit some activity on targets with TCTC PAM [Figures 5 and 11, Supplementary Figures S9–S12, S17 (23,31)]. The RR mutations slightly increase their tolerance on a target dependent manner in the case of Lb- and MbCpf1, but not for As- and FnCpf1 nucleases (Figure 11, second column, Supplementary Figure S18). Analyzing PAM position -2, we corroborate our earlier results (Figure 5) that all WT Cpf1 nucleases have low and target-dependent activity toward targets with TTCC PAMs (Figure 11, third column). The RR variants exhibit increased activity on many of the same targets, although this difference is less prominent in the case of Mb- and FnCpf1 nucleases (Figure 11, third column, Supplementary Figures S19 and S20). All WT Cpf1 nucleases have low target dependent activity on targets with C at both PAM positions -2 and -3 (TCCC PAMs, Figure 11, fourth column, Supplementary Figure S21) in a target-dependent manner similar to those seen with PAMs containing C in just one of the two positions (TCTC and TTCC, Figure 11, second and third column). In contrast, the four RR mutants have elevated activity on targets with TCCC PAMs, comparable to that which WT nucleases exhibit on the same targets with the "canonical" TTTC PAMs (Figure 11, fourth column). Interestingly, the data of each RR variant with TTCC and TCCC PAM in Figure 11 show an almost identical pattern. This suggests that the RR mutations actually affect the preferences for the -2 position of the PAM of these four nucleases with a smaller effect on the position -3. Next, we examined the effect of these RR mutations on the PAM specificities of these four Cpf1 nucleases toward single nucleotide changes at position -4. These mutations did not alter their tolerance; however, the overall activity of As- and FnCpf1 slightly decreased in a target- and nuclease-dependent manner (Supplementary Figures S22–S23).

**Figure 12.**
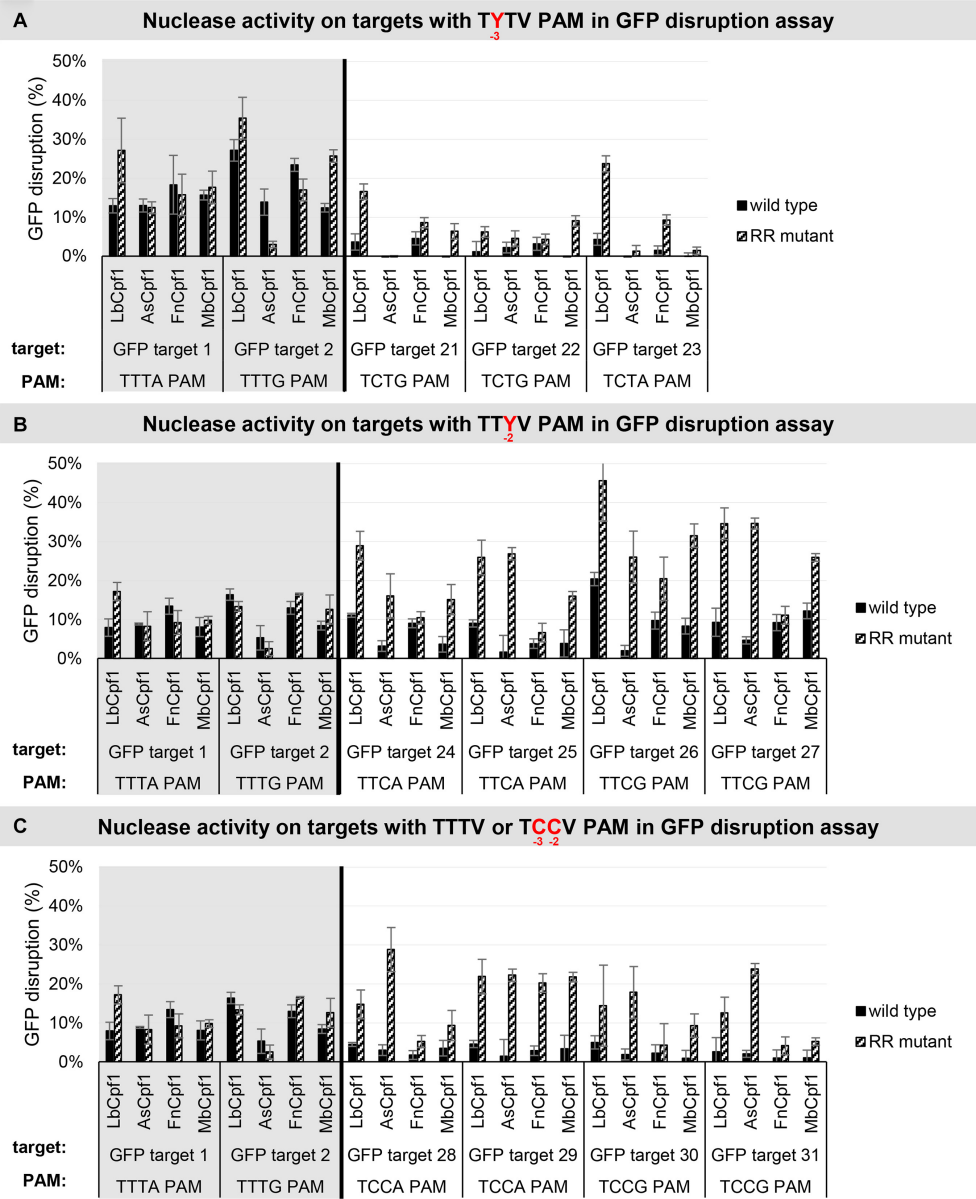
GFP disruption mediated by WT and RR Cpf1 variants in HEK-293.GFP cells. GFP disruption mediated by WT (black) and RR (striped) Cpf1 variants on targets with (**A**) TYTV, (**B**) TTYV or (**C**) TTTV/TCCV PAM sequences (TTTV PAM: GFP target 1–2, gray background; TCTV PAM: GFP target 21–24, TTCV A PAM: GFP target 25–28, TCCV PAM: GFP target 29–32, no background). The nuclease vector along with the corresponding crRNA expressing vector were transfected into HEK-293.GFP cells and GFP positive cells were counted 7 days post-transfection by flow cytometry. For controls we used either inactive Cpf1 expressing nuclease vectors or no-target crRNA expressing vectors. GFP disruption activities of the nucleases were calculated as follows: the measured percentage of GFP-positive cells for each sample was subtracted from the total average obtained with the controls. Bars correspond to averages of *n* = 3 parallel samples.

Next, we monitored the activities of the four RR variants on genomic targets with TCTV, TTCV and TCCV PAMs in comparison to the four WT proteins in a disruption assay using HEK-293.GFP cells (Figure 12). All four mutant nucleases demonstrated increased activities compared to the WT proteins on targets with TTCV or TCCV PAMs in a nuclease- and target-dependent manner, although these effects were much less prominent with the FnCpf1 mutant (Figure 12B, C). In contrast, on targets with a TCTV PAM a smaller effect is apparent, three variants (Lb-, Mb- and FnCpf1 RR variants) show increased activities, and among them, the RR variant of LbCpf1 is the most active when compared to the WT nucleases (Figure 12A). These results are in accord with the PAM preferences seen in the GFxFP assay (Figure 11).

## DISCUSSION

Cpf1 proteins are promising alternatives to the widely used Cas9 nucleases in genome editing applications. Among them, As- and LbCpf1s are employed in most of the applications at present, despite the rarity of their TTTV PAM sequence in the genome of higher eukaryotic organisms that limits their applications. Their potential alternatives, Fn- and MbCpf1 are reported to require shorter, two-thymidine-long PAM sequences and exhibited high cleavage activity *in vitro*, while their activity was hardly detectable in mammalian cells (23). In contrast, one of them, FnCpf1, in the presence of a DNA repair template, was demonstrated to induce precise gene insertions as well as indel mutations at the target site in the rice genome (40,41) and, in another study, in tobacco (50). More recently, Tu *et al.* showed that FnCpf1 exhibits indel-inducing activity in human cells on targets with KYTV PAM sequences (49). Here, we examined the activity of Fn- and MbCpf1 using two different methods, a plasmid-based assay and one exploiting genomic targets and revealed that in mammalian N2a cells both Fn- and MbCpf1 nucleases can be as useful as As- and LbCpf1 to induce HDR. This conclusion upon their potential usefulness was further supported on genomic targets in another mammalian cell line (HEK-293.GFP) exploiting NHEJ repair. Interestingly, when their indel-inducing activities were tested on endogenous targets by next-generation sequencing, Lb- and AsCpf1 performed better. By comparing the four nucleases on identical targets using the different assays, a nonidentical, although similar target selectivity is discernible that may explain why the activity of Fn- and MbCpf1 nucleases was not revealed in earlier studies in mammalian cells when only one or additional two targets, were examined (23,29). This also suggests that some other Cpf1 nucleases investigated by the Zetsche study (23) may prove to be useful in mammalian cells if they are tested on increased number of targets.

Our experiments also suggest a slightly different PAM requirement for As- and LbCpf1 nucleases than that was originally described (23). We confirm that both nucleases prefer T in position -2, -3 and -4, but disfavor it in position -1. The latter effect is more evident with AsCpf1 than with LbCpf1 (Figures 5 and 6). A slight preference for A at position -1 may also be observable for AsCpf1, but not for LbCpf1 (Figure 5), in line with the previous study (31). In contrast, we found that LbCpf1 is slightly more permissive for a C nucleotide in either the second or fourth positions than AsCpf1, also in accord with (31). Our approach, using 96 PAM-target combinations for each nuclease, appears to reveal the same features of PAM requirements for Lb- and AsCpf1 as the more systematic study of Kim *et al.* exploring approximately 1100 combinations (31), underscoring the validity of our approach. By the same argument the PAM preferences for the Fn- and MbCpf1 nucleases are also likely to be generally valid.

Based on the crystal structure of FnCpf1 with crRNA and target DNA, interactions of the protein with positions -2 and -3 but not the first base pair of the PAM sequence were reported supporting a TTN PAM requirement (51,52) as was suggested in (23) for both Fn- and MbCpf1 based on *in vitro* assays. Here, we confirm the T preferences for both the second and third positions concerning both nucleases. However, our results further refine the understanding of the PAM requirements of both nucleases in mammalian cells. Both Fn- and MbCpf1 disfavor T nucleotide in position -1 although MbCpf1 is somewhat more tolerant, similar to LbCpf1 (Figures 5 and 6). By contrast, they prefer a T nucleotide in position -4 leading to the same TTTV PAM sequence that has been established for As- and LbCpf1. The structural bases of this preference are not fully understood. However, the -4 T preference of Fn- and MbCpf1 is less prominent than that of As- and LbCpf1. Fn- and MbCpf1 are also permissive for a C nucleotide as a secondary choice at positions -2 and -3 of TTTV (Figures 5 and 6). Interestingly, the permissiveness of all four nucleases for a C nucleotide as a second choice at positions -2, -3 or -4 or a G nucleotide as a second choice for FnCpf1 at position -4 varies with the target sequences and shows an apparent target dependency (Supplementary Figures S9–12). This might also explain the slight differences for the -4 PAM preferences of FnCpf1 reported by Tu *et al.* (49), systematically examining two targets in contrast to the 12 different targets exploited in this study. Although none of the two Cpf1 nucleases proved to possess only a shorter TTN PAM in mammalian cells, due to their differing activities on various targets, these experiments established Fn- and MbCpf1 as useful complement to As- and LbCpf1 for genetic engineering in mammalian cells.

To further increase the number of available targets of Cpf1 nucleases in higher eukaryotic genomes we generated the RVR and RR variants of Fn- and MbCpf1 analogous to those of As- and LbCpf1 (44) based on their sequence homology (Supplementary Figure S13). The RVR and RR mutations in Fn- and MbCpf1 resulted in PAM recognition patterns very similar to those of As- and LbCpf1 analogous mutants. Thus, our results suggest that despite some sequence differences in the implicated region between the four Cpf1s, these nucleases employ an identical mechanism for PAM recognition.

One interesting outcome of the side-by-side comparison of the mutants to the WT nucleases is that the RVR mutant variants, beside gaining new PAM recognition abilities, retained activity on targets with the canonical TTTV PAM comparable to that of the WT proteins as reported earlier for AsCpf1 (45). Thus, our results further refine our understanding of the PAM preferences of this variant of LbCpf1 and suggest that these RVR mutants are capable of efficiently mediating genome editing on targets with TWTV PAM. For RR mutants, the LbCpf1 variant utilizes a TYYV PAM, while the AsCpf1 variants utilize TTYV or TCCV PAM, since the activities of the RR mutant of AsCpf1 on the same targets with TCTV PAM do not seem to exceed those of the corresponding WT proteins. The RR mutant of FnCpf1 is more similar to LbCpf1, whereas MbCpf1 is more similar to AsCpf1.

In accord with the observed target dependency of the PAM recognition of the WT Cpf1s, the mutations increased the tolerances of the variants to the altered PAMs in a nuclease- and target-dependent manner. Thus, although the PAM preferences of the mutants of Fn- and MbCpf1 generated here are very similar to those of the As- and LbCpf1 variants, they increase the number of the targets that are available for genome modifications by Cpf1s.

In conclusion, our experiments demonstrate that Fn- and MbCpf1 can be used to perform genome engineering tasks with a considerable efficiency in mammalian cells. However, neither Fn-, nor MbCpf1 demonstrate a general higher activity on targets with VTTV PAM sequences compared to As- or LbCpf1, suggesting that the sequence requirements in mammalian cells for As- and LbCpf1 are more relaxed than those identified *in vitro* whereas those for Fn- and MbCpf1 are stricter. Nevertheless, since their target specificities are not identical and because there is a target dependency of their PAM preferences, they seem destined to become useful complements to As- and LbCpf1. We also generated the RVR and RR mutant variants of both Fn- and MbCpf1 and characterized them along with the analogous As- and LbCpf1 variants and the corresponding WT proteins. Our results revealed that the mutations altered the PAM preferences of all four Cpf1s in a similar manner and redefined the known PAM preferences of the As- and LbCpf1 variants: RVR mutants recognize targets with TWTV PAMs, while RR mutants prefer targets with TYYV or TTYV/TCCV PAMs. We also showed here that the mutant variants retained their activity on the canonical TTTV PAM, suggesting that they are superior alternatives with a more relaxed PAM recognition compared to the WT proteins in most applications.

## Supplementary Material

Supplementary Data
