# Supplementary material for: Mb- and FnCpf1 nucleases are active in mammalian cells: activities and PAM preferences of four wild-type Cpf1 nucleases and of their altered PAM specificity variants
Source: Nucleic Acids Res. 2018 Sep 20;46(19):10272–85. doi: 10.1093/nar/gky815 (PMC6212782; doi:10.1093/nar/gky815)
Supplement: Supplementary Data [file gky815_supplemental_files.zip › 2018-08-30 Supplementary information_revised-final-proof.docx]

**Supplementary Data**

**Supplementary Figures**


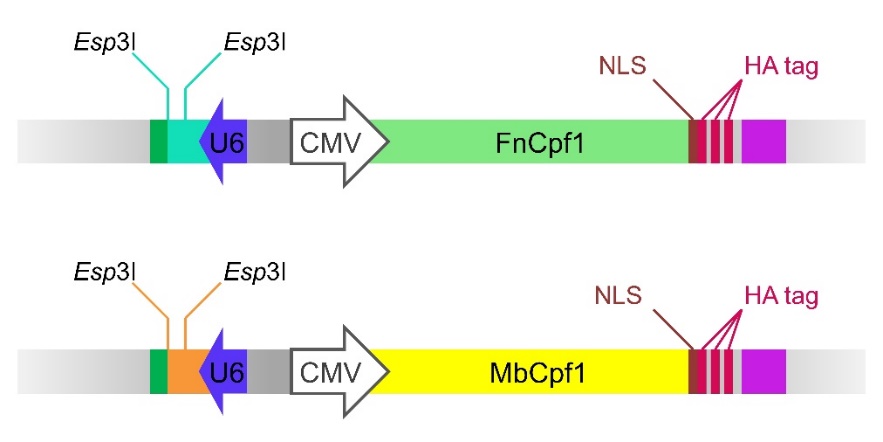


**Supplementary Figure S1. Schematic structure of Fn- (pTE4497) and MbCpf1 (pTE4495) expression plasmids with crRNA expression cassettes**

To facilitate an easier use of these Cpf1 nucleases against various targets, we cloned the Fn- and Mb crRNAs driven by a human U6 promoter into the pY004 and pY014 plasmids (23), respectively, in such a way that a spacer can be precisely inserted at the 3' end of the crRNA between two sites of a type IIS restriction endonuclease (*Esp*3I). The plasmids created (pTE4497 for FnCpf1 and pTE4495 for MbCpf1) are deposited at Addgene (#80339 and #80338, respectively).

green: RNA polymerase III terminator, light blue or orange: Fn- or MbCpf1 crRNAs, respectively, blue: human U6 promoter, white: CMV enhancer and promoter, light green or yellow: Fn- or MbCpf1, respectively, scarlet: nuclear localization signal sequence (NLS), pink: three consecutive HA tags, purple: bGH poly(A) signal

**
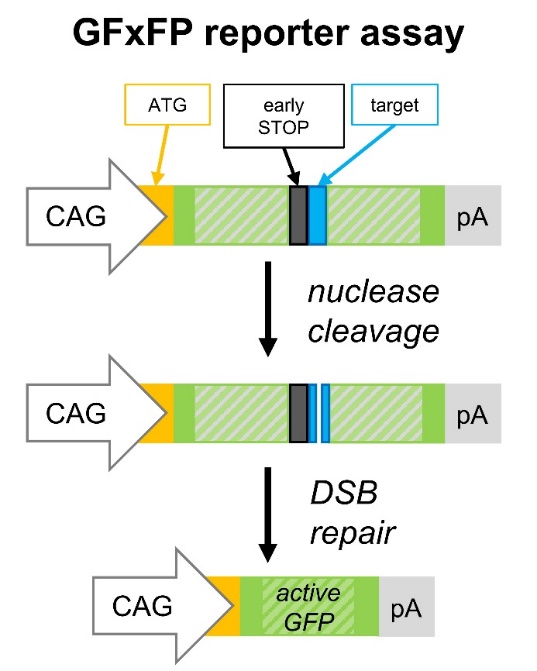
**

**Supplementary Figure S2. GFxFP reporter assay (19)**

Schematics of the principle of the reporter assay used for monitoring the activity of nucleases. The sequences of the two GFP halves are indicated in green. Each of them contains both a non-overlapping segment (green), and an overlapping segment (green with grey lines). The expression cassette is driven by a CAG promoter (white arrow) and terminated by a SV40 (Simian Vacuolating Virus 40) polyA signal (pA, grey box). Expression from the first GFP half is terminated by an early STOP codon (black mark), and thus, results in no detectable green fluorescence. The second GFP half has neither Kozak sequence nor ATG. Upon nuclease cleavage at the target site (blue box) the generated double-strand DNA break can be repaired by HDR of the overlapping homologous sequences. Thus, an intact GFP sequence is created from which a functional fluorescent GFP (green) is transcribed*.*

**Supplementary Figure S3. Comparison of cleavage efficiencies of different Cpf1 nucleases in plasmid based GFxFP assay in mouse N2a cells**

Percentages of GFP positive cells counted above the background level, resulting from the action of various nucleases. Thirteen randomly picked targets cloned into the pGF-ori-FP vector (19) were tested using the GFxFP assay. The target vectors along with the corresponding nuclease vector were transfected into N2a cells and GFP positive cells were counted two days after transfection. All samples were also cotransfected with an mCherry expression vector to monitor the transfection efficiency and the GFP signal was analysed within the mCherry positive population. The background fluorescence was estimated by using a crRNA-less, inactive AsCpf1 nuclease expression vector as negative control and was subtracted from each sample. Three parallel transfections were made for each case. Error bars show the mean ± standard deviation of percentages measured in n=3 independent transfections. **Fn- and MbCpf1 nucleases demonstrate activity comparable to that of As- and LbCpf1.**


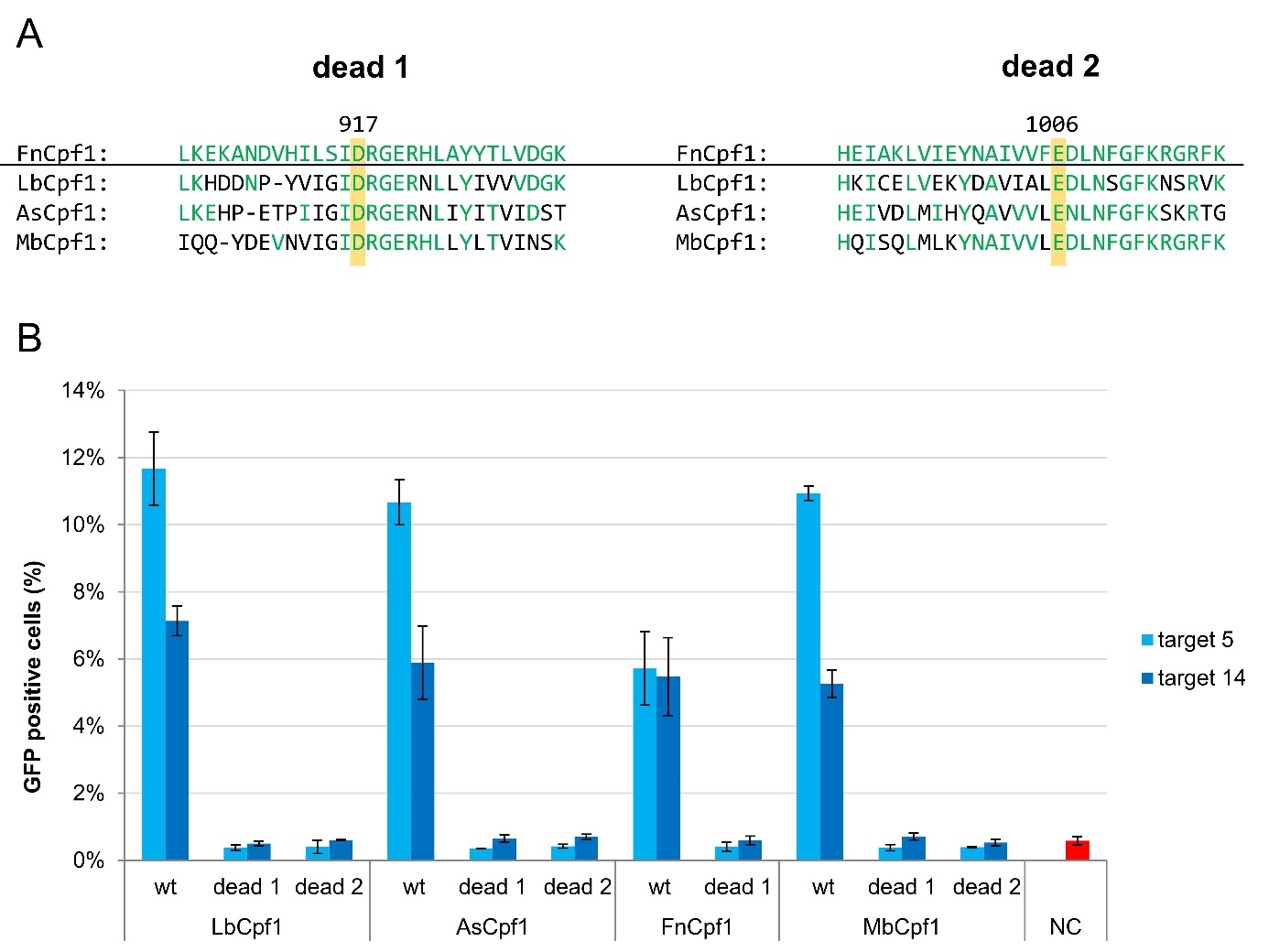


**Supplementary Figure S4. Activity of different Cpf1 RuvC mutants on two different targets in mammalian N2a cells**

**A:** Alignment of protein segments of different Cpf1 nucleases flanking the RuvC inactivating mutations (green: match, black: no match or gap). RuvC inactive mutants of FnCpf1 nuclease were characterized by Zetsche and his co-workers (23) in bacteria. Based on these mutations we identified an aspartic acid and a glutamic acid residue in Lb-, As- and MbCpf1 and generated the following mutants, *dead 1*: LbCpf1(D832A), AsCpf1(D908A), MbCpf1(D986A), *dead 2*: LbCpf1(E925A), AsCpf1(E993A), MbCpf1(E1080A).

**B:** Percentages of GFP positive cells counted above the background level, resulting from the action of various nucleases (LbCpf1, AsCpf1, FnCpf1, MbCpf1). Two target sequences (target 5 and target 9) cloned into the pGF-ori-FP vector (19) were explored in a GFxFP assay. The target vectors along with the corresponding nuclease vector were transfected into N2a cells and GFP positive cells were counted two days after transfection. All samples were also cotransfected with an mCherry expression vector to monitor the transfection efficiency and the GFP signal is analyzed within the mCherry positive population. The background fluorescence was estimated by using a guideRNA-less, inactive SpCas9 nuclease expression vector (NC = negative control, red column). Three parallel transfections were made for each case. Error bars show the mean ± standard deviation measured in n=3 independent transfections. wt: wild type

**Supplementary Figure S5. SpCas9 nucleases induced HDR-mediated integration at various genomic cleavage sites**

Percentages of GFP fluorescent cells after HDR-mediated integration of a donor GFP cassette. The efficiency with which SpCas9 nuclease induced HDR-mediated integration was tested on six mouse *Prnd* genomic targets (PRND 1, 2a, 2b, 3a, 3b, 5-6). These targets were selected to overlap with the Cpf1 PRND targets on Figure 3. The nuclease vector and the homologous recombination donor molecule (with – green – or, as control, without – red – the corresponding homologous arms) were cotransfected into N2a cells. As another negative control, cells were transfected with the donor molecule and an inactive SpCas9 expression plasmid. On the fourteenth day after transfection GFP positive cells were counted. Three parallel transfections were made for each sample. Two days after transfection all samples showed similar GFP positive cell counts. This GFP fluorescence was used to normalize the results for variation in transfection efficiency. Error bars show the mean ± standard deviation of percentages measured in n=3 independent transfections.


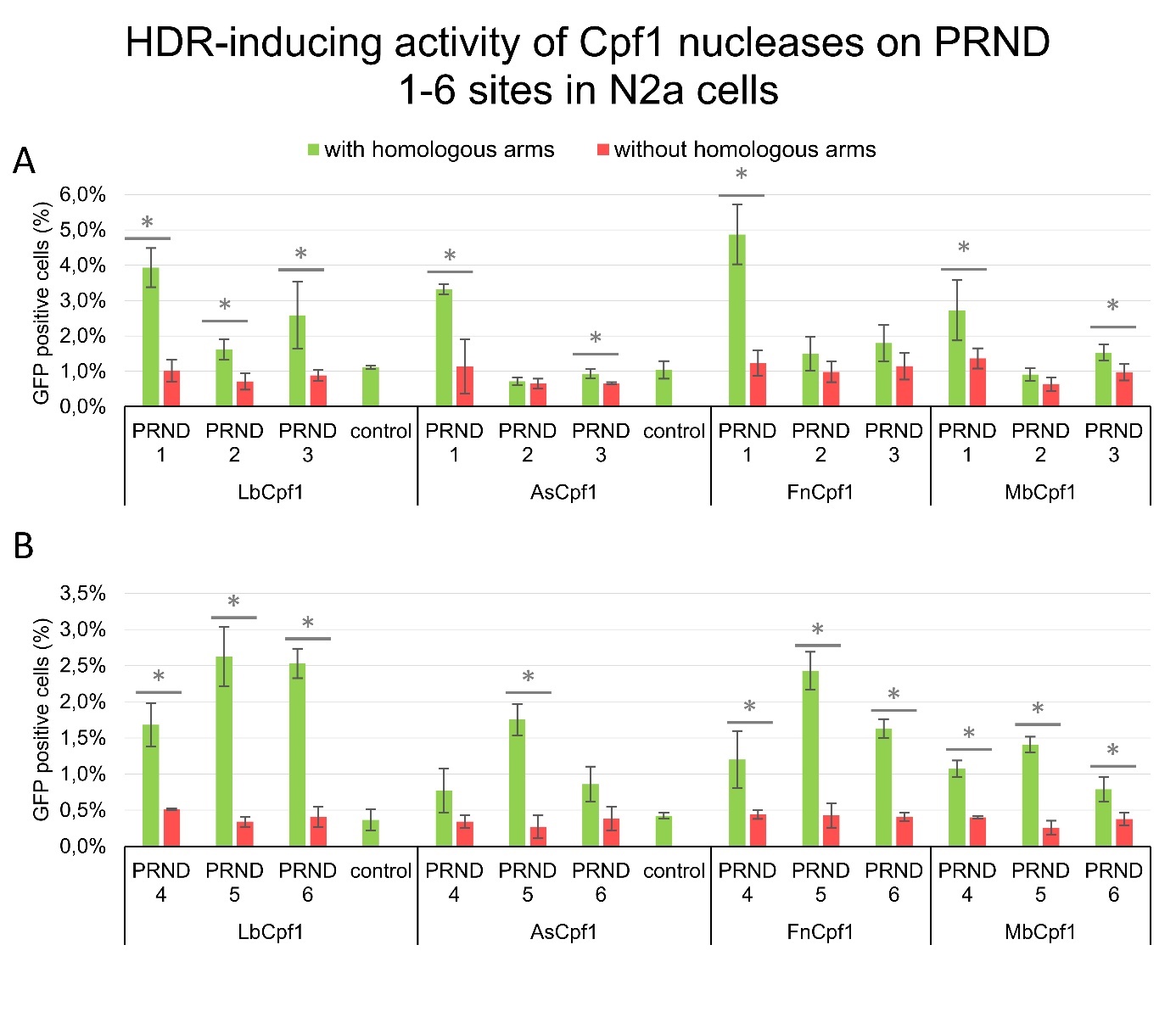


**Supplementary Figure S6. Cpf1 nucleases induced HDR-mediated integration at various genomic cleavage sites**

Percentages of GFP fluorescent cells after HDR-mediated integration of a donor GFP cassette. The efficiency with which Cpf1 nucleases induced HDR-mediated integration was tested on six mouse *PRND* genomic targets (**A:** PRND 1-3, **B:** PRND: 4-6). The nuclease vector and the homologous recombination donor molecule (with or, as control, without the corresponding homologous arms) were co-transfected into N2a cells. As another negative control, cells were transfected with the donor molecule and an inactive Lb- or AsCpf1 expression plasmid without crRNA expression cassette (control with homologous arms). On the fourteenth day after transfection GFP positive cells were counted. Three parallel transfections were made for each sample. Two days after transfection all the samples showed similar GFP positive cell counts. This GFP fluorescence was used to normalize the results for variation in transfection efficiency. Error bars show the mean ± standard deviation of percentages measured in n=3 independent transfections. Student's t-test for independent samples was used to compare means of corresponding samples (with or without homologous arms) (SPSS 9.0 Statistica Program). p value: * p<0.05. **Fn- and MbCpf1 nucleases are as active in mammalian cells as As- and LbCpf1 in mediating homology directed integration of a donor DNA cassette.**


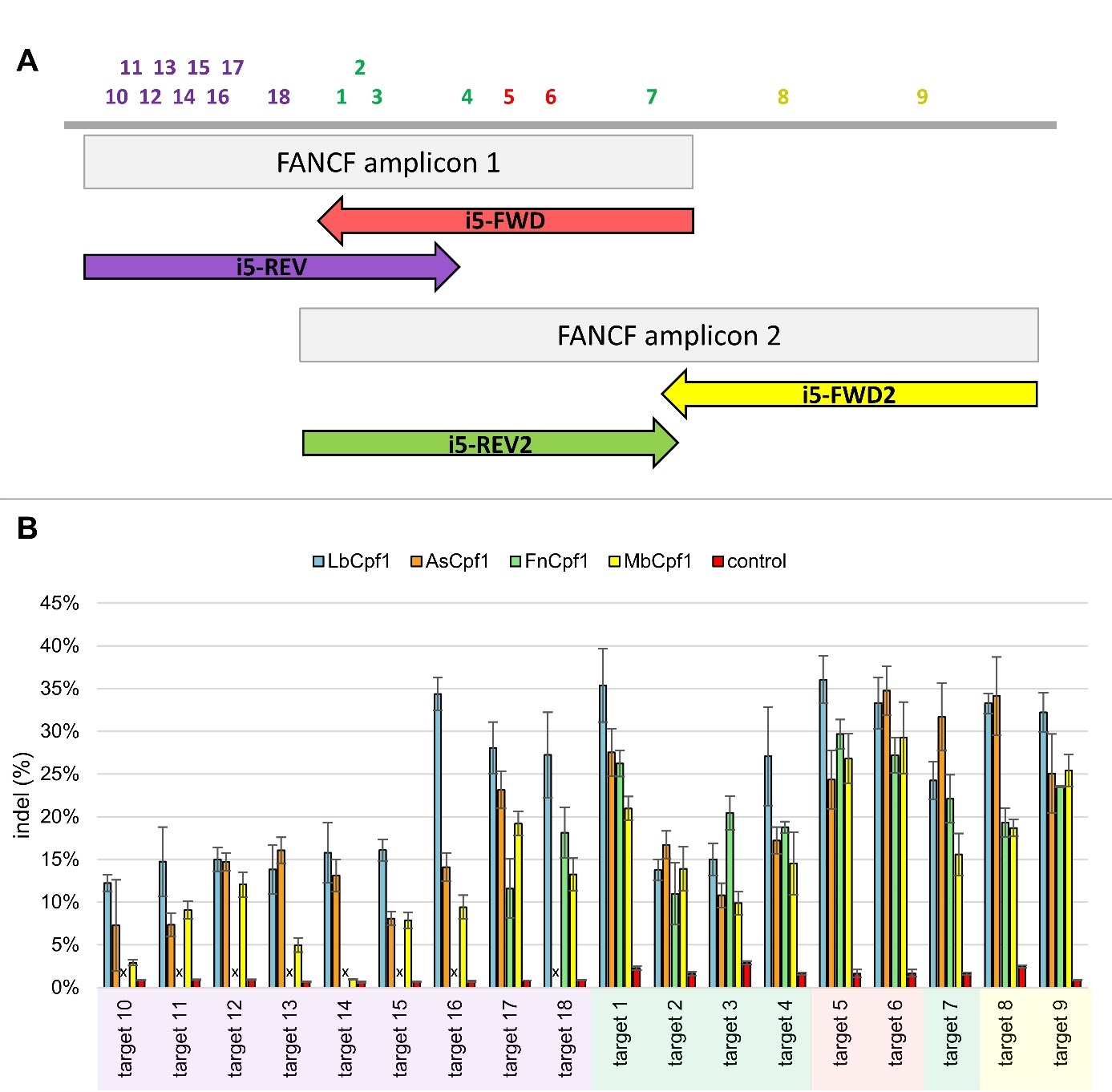


**Supplementary Figure S7. Indels mediated by different Cpf1 nucleases on human endogenous targets 1-18 (FANCF)**

**A:** Location of the targets chosen around the FANCF genomic site. Two amplicons (FANCF amplicon 1 and 2) were PCR amplified from genomic DNA and indexed for next generation sequencing. Amplicons were sequenced from either forward or reverse direction resulting in i5-FWD (red), i5-REV (purple), i5-FWD2 (green) and i5-REV2 (yellow) reference sequences. The relative position of the target sites is marked by the numbers on the top, and their colouring indicates the reference sequence for alignment. **B:** Indel percentages of different targets resulting from Cpf1 (blue – LbCpf1, orange – AsCpf1, green – FnCpf1, yellow – MbCpf1) nuclease cleavage compared to the control, untransfected cells (red). Samples corresponding to FnCpf1 activity at target 10-16 and to AsCpf1 activity at target 18 are not examined (their absence is marked by x on the graph). Average transfection efficiency was ~51%. Bars correspond to averages of n = 3 parallel samples.

**
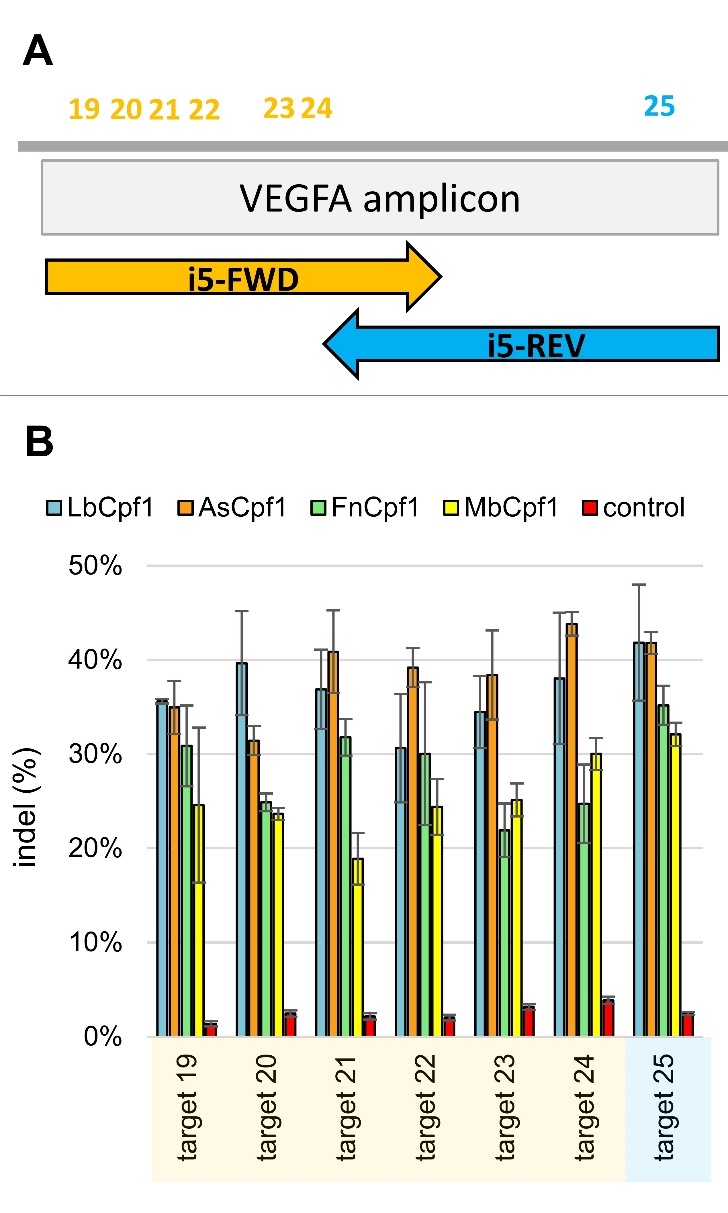
**

**Supplementary Figure S8. Indels mediated by different Cpf1 nucleases on human endogenous targets 19-25 (VEGFA)**

**A:** Chosen targets located around VEGFA genomic site. An amplicon (VEGFA amplicon) was PCR amplified from genomic DNA and indexed for next generation sequencing. The VEGFA amplicon was sequenced from either forward or reverse direction resulting in i5-FWD (orange) and i5-REV (blue) reference sequences. The position of the target sites is marked by the numbers on the top, and their colouring matches the colour of the reference sequence for alignment. **B:** Indel percentages of different targets resulting from Cpf1 (blue – LbCpf1, orange – AsCpf1, green – FnCpf1, yellow – MbCpf1) nuclease cleavage compared to the control, untransfected cells (red). Average transfection efficiency was ~61%. Bars correspond to averages of n = 3 parallel samples.


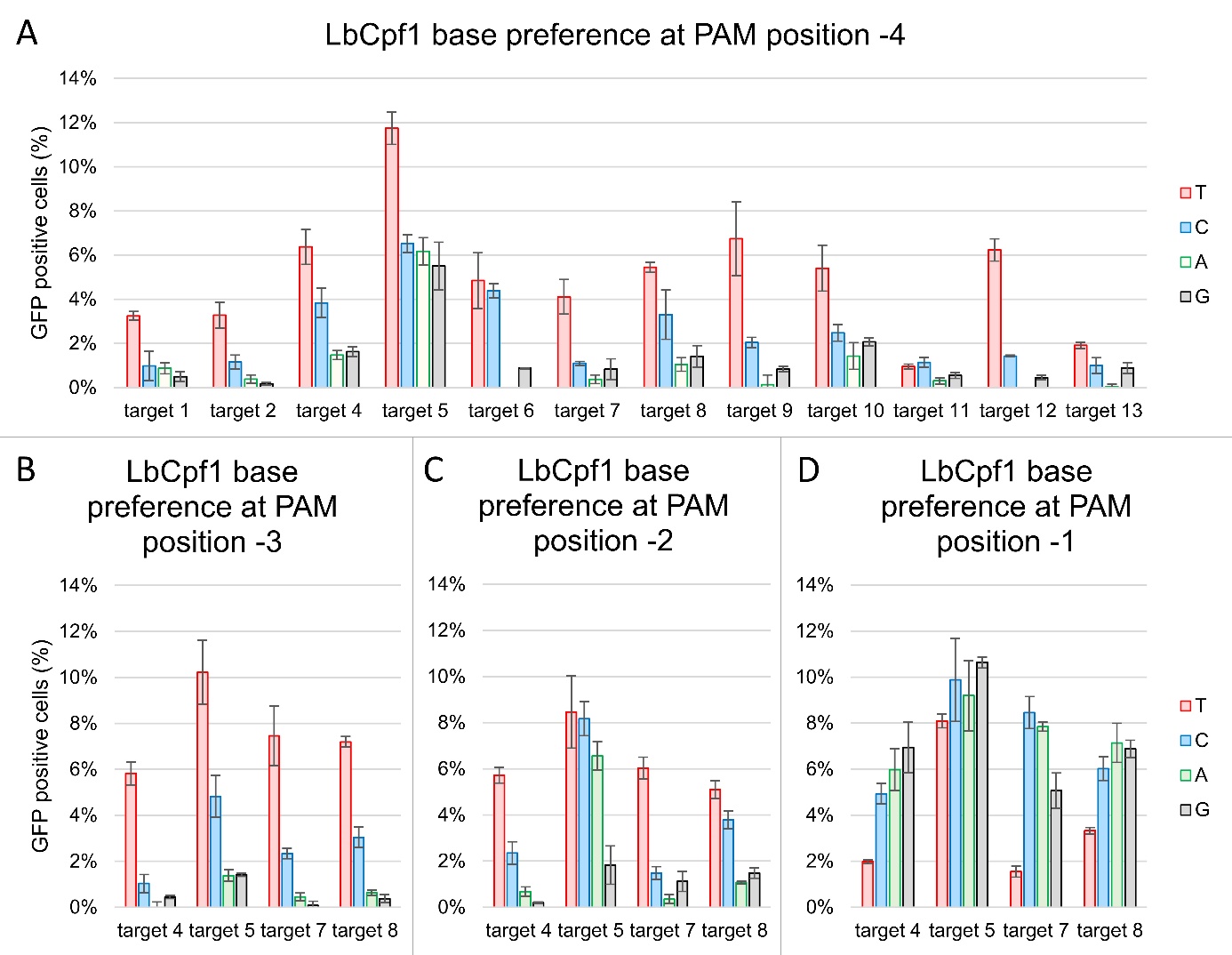


**Supplementary Figure S9. Detailed results of LbCpf1 PAM screen**

Base preference of LbCpf1 nuclease measured at PAM position **A:** -4 (NTTC), **B:** -3 (TNTC), **C:** -2 (TTNC) and **D:** -1 (TTTN). The effect of different PAM sequences was measured on four to twelve different targets with the GFxFP assay (19). Percentages of GFP positive cells counted above the background level resulting from the action of LbCpf1 are shown. The target vectors along with the corresponding nuclease vectors were transfected into N2a cells and GFP positive cells were counted two days after transfection. All samples were also cotransfected with an mCherry expression vector to monitor the transfection efficiency and the GFP signal is analysed within the mCherry positive population. The background fluorescence was estimated by using a crRNA-less, inactive AsCpf1 nuclease expression vector as negative control and was subtracted from each sample. Three parallel transfections were made for each case. Error bars show the mean ± standard deviation of percentages measured in n=3 independent transfections. T (thymidine): red, C (cytosine): blue, A (adenosine): green, G (guanine): grey.

**
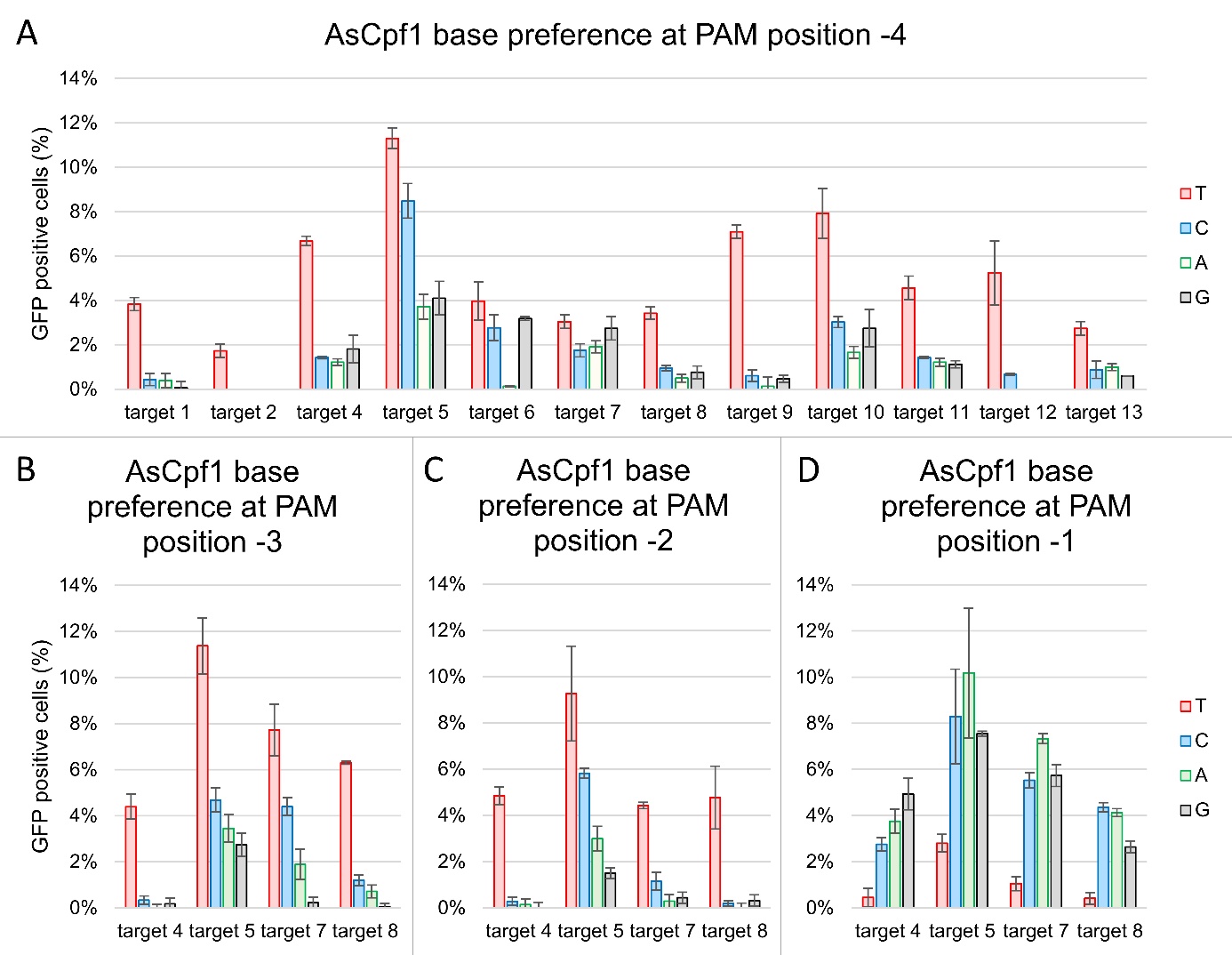
**

**Supplementary Figure S10. Detailed results of AsCpf1 PAM screen**

Base preference of AsCpf1 nuclease measured at PAM position **A:** -4 (NTTC), **B:** -3 (TNTC), **C:** -2 (TTNC) and **D:** -1 (TTTN). The effect of different PAM sequences was measured on four to twelve different targets with the GFxFP assay (19). Percentages of GFP positive cells counted above the background level resulting from the action of AsCpf1 are shown. The target vectors along with the corresponding nuclease vectors were transfected into N2a cells and GFP positive cells were counted two days after transfection. All samples were also cotransfected with an mCherry expression vector to monitor the transfection efficiency and the GFP signal was analysed within the mCherry positive population. The background fluorescence was estimated by using a crRNA-less, inactive AsCpf1 nuclease expression vector as negative control and was subtracted from each sample. Three parallel transfections were made for each case. Error bars show the mean ± standard deviation of percentages measured in n=3 independent transfections. T (thymidine): red, C (cytosine): blue, A (adenosine): green, G (guanine): grey.


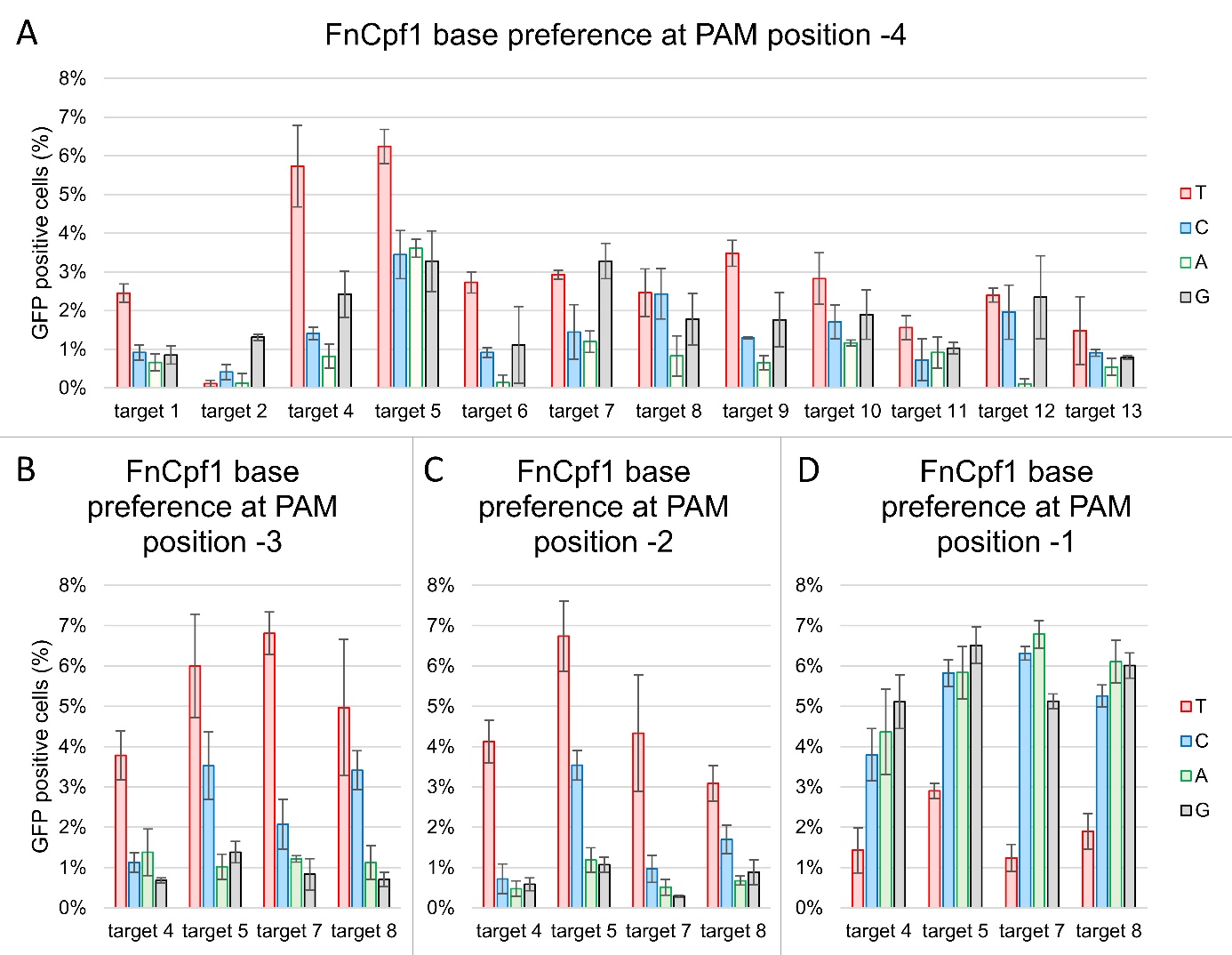


**Supplementary Figure S11. Detailed results of FnCpf1 PAM screen**

Base preference of FnCpf1 nuclease measured at PAM position **A:** -4 (NTTC), **B:** -3 (TNTC), **C:** -2 (TTNC) and **D:** -1 (TTTN). The effect of different PAM sequences was measured on four to twelve different targets with the GFxFP assay (19). Percentages of GFP positive cells counted above the background level resulting from the action of FnCpf1 are shown. The target vectors along with the corresponding nuclease vectors were transfected into N2a cells and GFP positive cells were counted two days after transfection. All samples were also cotransfected with an mCherry expression vector to monitor the transfection efficiency and the GFP signal was analysed within the mCherry positive population. The background fluorescence was estimated by using a crRNA-less, inactive AsCpf1 nuclease expression vector as negative control and was subtracted from each sample. Three parallel transfections were made for each case. Error bars show the mean ± standard deviation of percentages measured in n=3 independent transfections. T (thymidine): red, C (cytosine): blue, A (adenosine): green, G (guanine): grey.


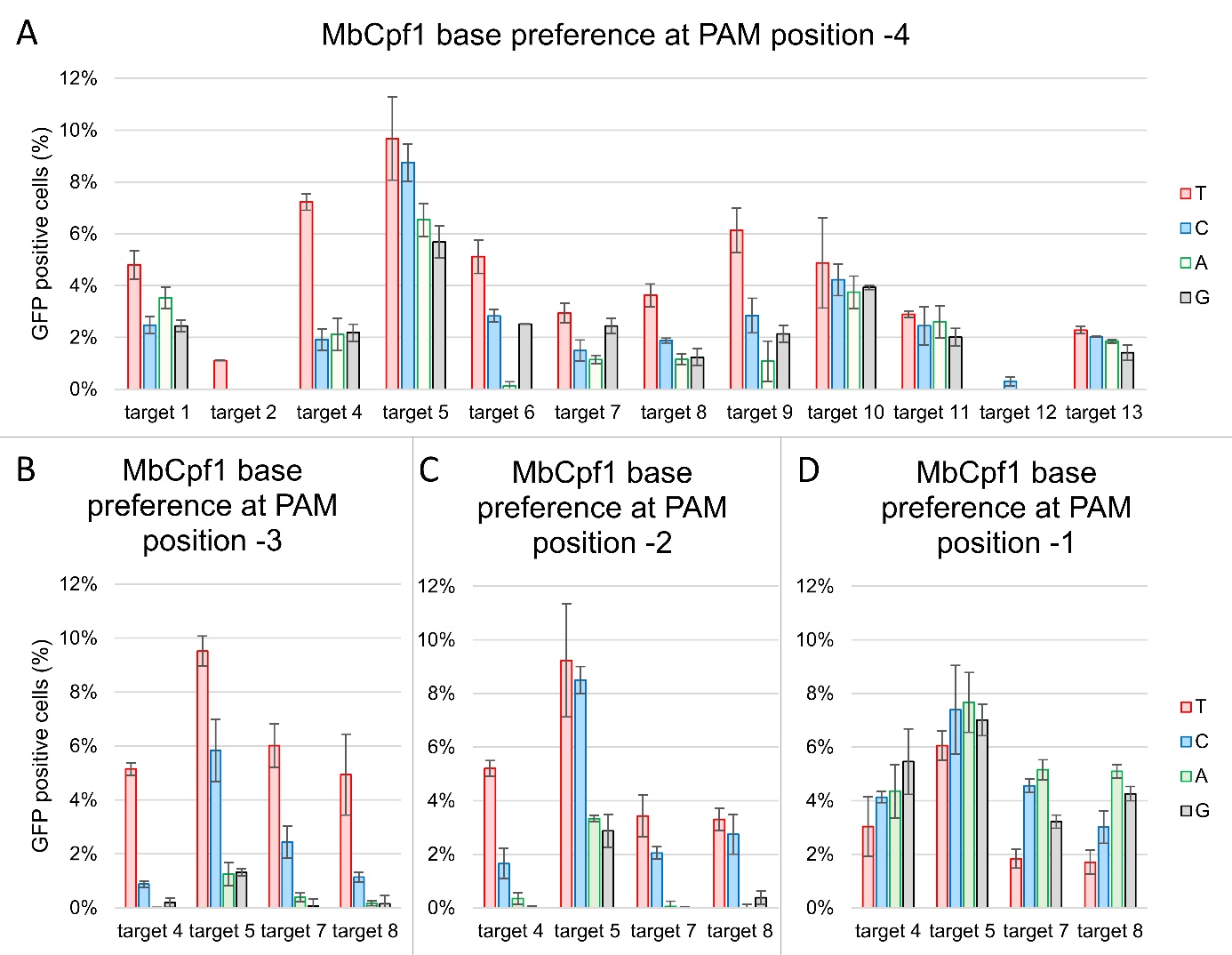


**Supplementary Figure S12. Detailed results of MbCpf1 PAM screen**

Base preference of MbCpf1 nuclease measured at PAM position **A:** -4 (NTTC), **B:** -3 (TNTC), **C:** -2 (TTNC) and **D:** -1 (TTTN). The effect of different PAM sequences was measured on four to twelve different targets with the GFxFP assay (19). Percentages of GFP positive cells counted above the background level resulting from the action of MbCpf1 are shown. The target vectors along with the corresponding nuclease vectors were transfected into N2a cells and GFP positive cells were counted two days after transfection. All samples were also cotransfected with an mCherry expression vector to monitor the transfection efficiency and the GFP signal was analysed within the mCherry positive population. The background fluorescence was estimated by using a crRNA-less, inactive AsCpf1 nuclease expression vector as negative control and was subtracted from each sample. Three parallel transfections were made for each case. Error bars show the mean ± standard deviation of percentages measured in n=3 independent transfections. T (thymidine): red, C (cytosine): blue, A (adenosine): green, G (guanine): grey.


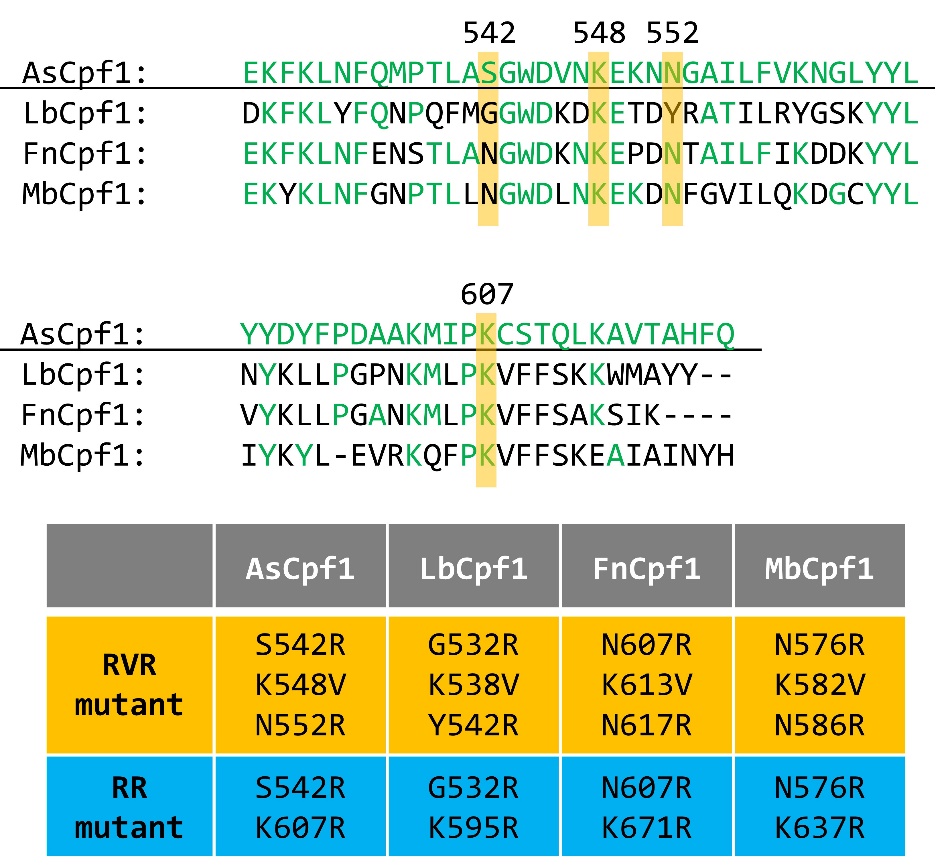


**Supplementary Figure S13. Altered PAM recognition mutants of different Cpf1 nucleases**

Alignment of protein segments of different Cpf1 nucleases flanking the PAM recognition altering mutations (green: match, black: no match or gap). Altered PAM recognition mutants of As- and LbCpf1 nucleases were characterized by Gao and his co-workers (44). Based on these mutations we identified and generated the following mutants: *RVR mutants (orange background):*  FnCpf1(N607R, K613V, N617R), MbCpf1(N576R, K582V, N586R), *RR mutants (blue background):* FnCpf1(N607R, K671R), MbCpf1(N576R, K637R).


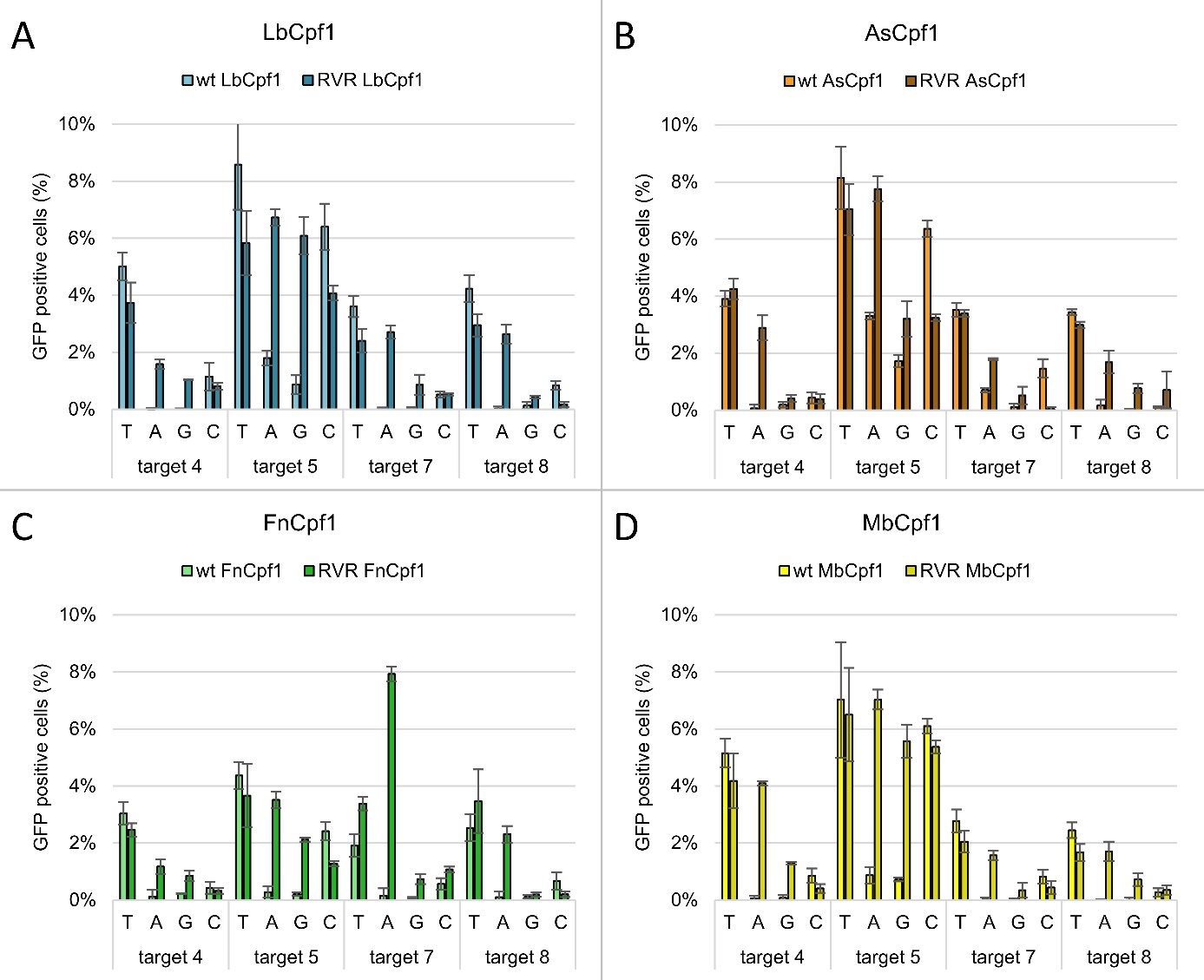


**Supplementary Figure S14. Detailed results of wild type and RVR mutant nuclease activity on targets with TNTC PAM sequence**

We compared the activity of wild type and RVR mutant Cpf1 nucleases on targets with TNTC PAM sequence in the GFxFP assay. Percentages of GFP positive cells counted above the background level resulting from the action of **A:** LbCpf1 (blue), **B:** AsCpf1 (orange), **C:** MFnCpf1 (green), **D:** MbCpf1 (yellow) are shown. The target vectors along with the corresponding nuclease vectors were transfected into N2a cells and GFP positive cells were counted two days after transfection. All samples were also cotransfected with an mCherry expression vector to monitor the transfection efficiency and the GFP signal was analysed within the mCherry positive population. The background fluorescence was estimated by using a crRNA-less, inactive LbCpf1 nuclease expression vector as negative control and was subtracted from each sample. Three parallel transfections were made for each case. Error bars show the mean ± standard deviation of percentages measured in n=3 independent transfections.

**
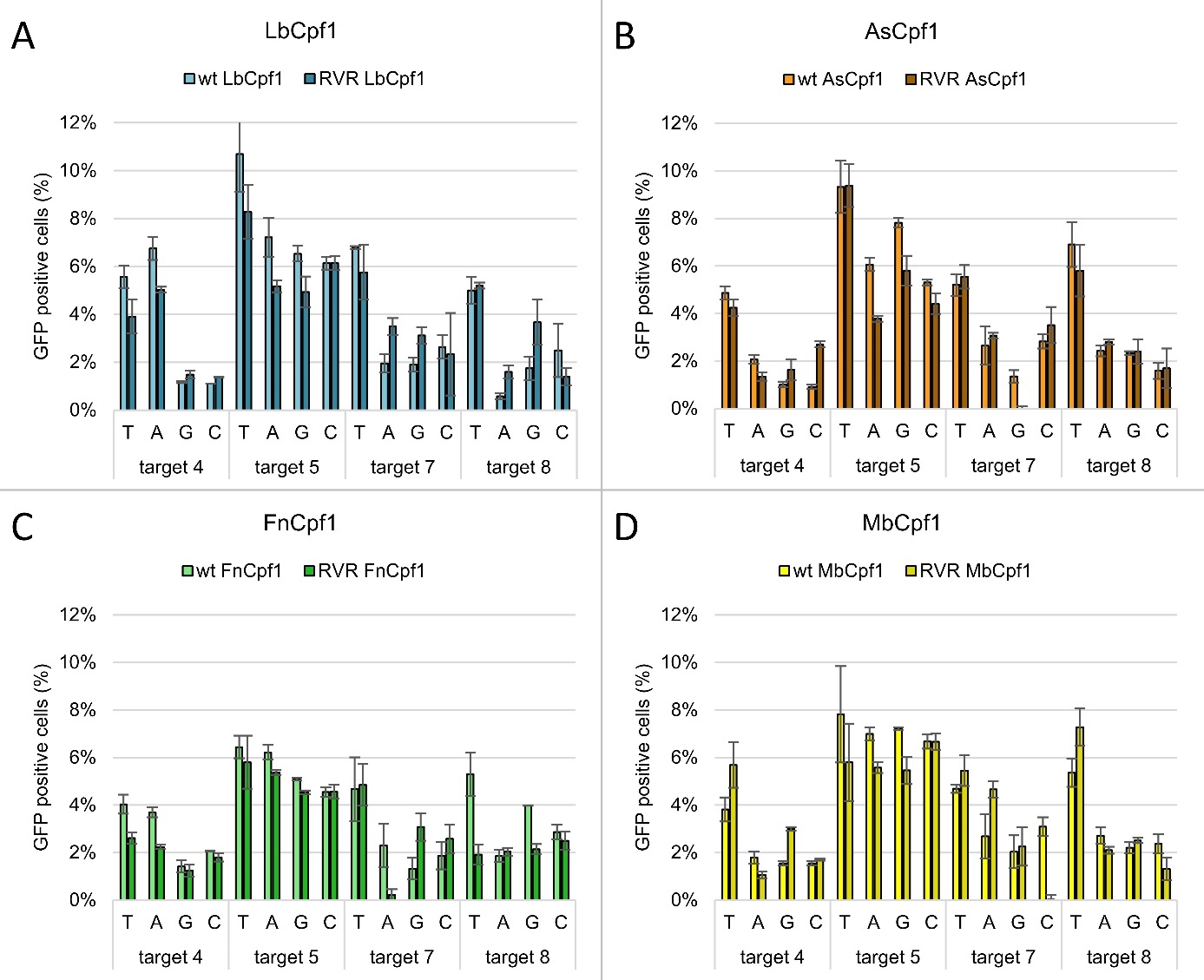
**

**Supplementary Figure S15. Detailed results of wild type and RVR mutant nuclease activity on targets with NTTC PAM sequence**

We compared the activity of wild type and RVR mutant Cpf1 nucleases on targets with NTTC PAM sequence in the GFxFP assay. Percentages of GFP positive cells counted above the background level resulting from the action of **A:** LbCpf1 (blue), **B:** AsCpf1 (orange), **C:** FnCpf1 (green), **D:** MbCpf1 (yellow) are shown. The target vectors along with the corresponding nuclease vectors were transfected into N2a cells and GFP positive cells were counted two days after transfection. All samples were also cotransfected with an mCherry expression vector to monitor the transfection efficiency and the GFP signal was analysed within the mCherry positive population. The background fluorescence was estimated by using a crRNA-less, inactive LbCpf1 nuclease expression vector as negative control and was subtracted from each sample. Three parallel transfections were made for each case. Error bars show the mean ± standard deviation of percentages measured in n=3 independent transfections.

**Supplementary Figure S16. Base preference of wild type and RVR mutant nucleases at PAM position -4**

Ratios of activities of the four nucleases averaged over targets in which the nucleotides have been varied systematically at PAM position -4 (NTTC). Green: thymidine, purple: cytosine, orange: adenine, blue: guanine.


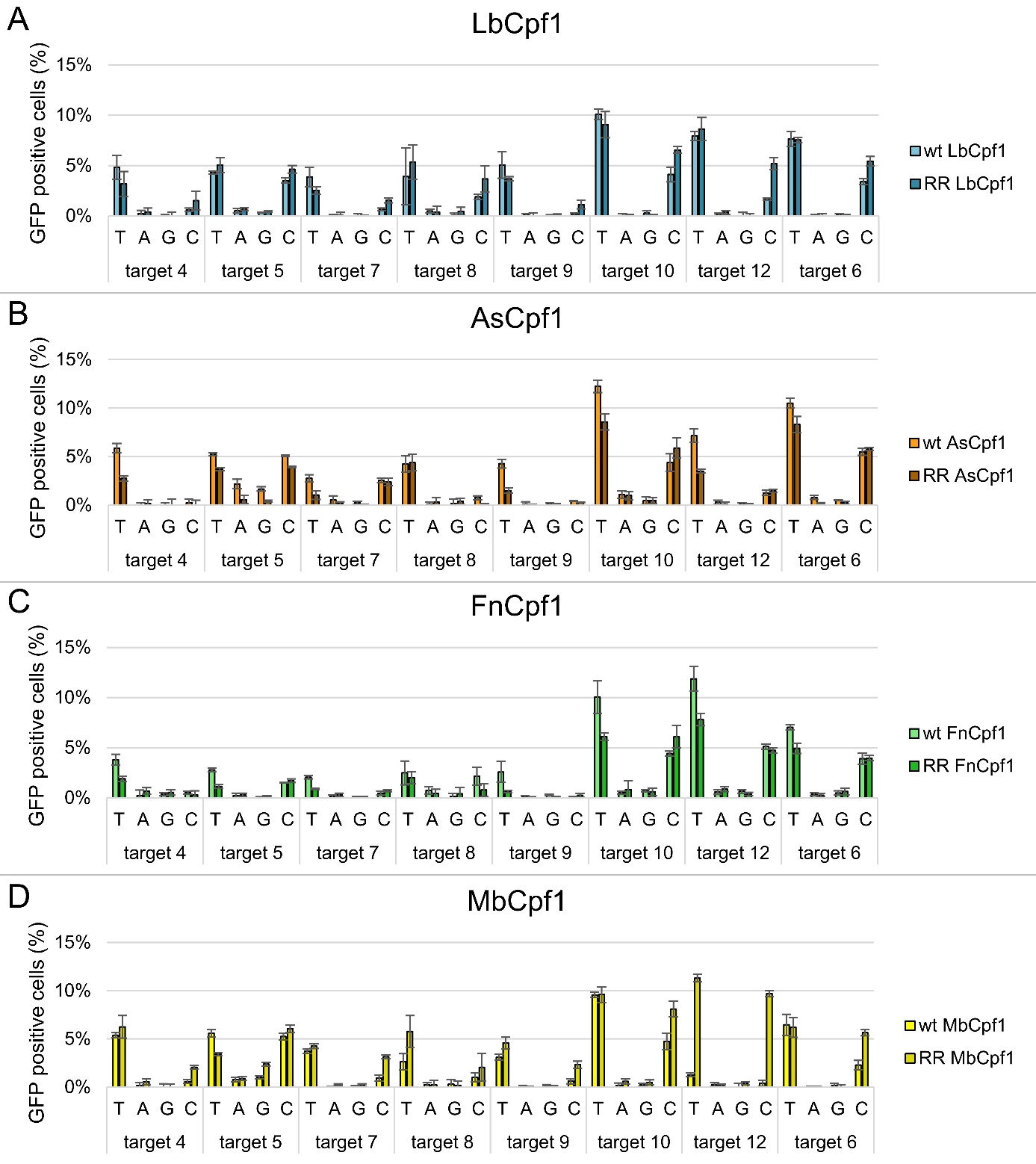


**Supplementary Figure S17. Detailed results of wild type and RR mutant nuclease activity on targets with TNTC PAM sequence**

We compared the activity of wild type and RR mutant Cpf1 nucleases on targets with TNTC PAM sequence in the GFxFP assay. Percentages of GFP positive cells counted above the background level resulting from the action of **A:** LbCpf1 (blue), **B:** AsCpf1 (orange), **C:** FnCpf1 (green), **D:** MbCpf1 (yellow) are shown. The target vectors along with the corresponding nuclease vectors were transfected into N2a cells and GFP positive cells were counted two days after transfection. All samples were also cotransfected with an mCherry expression vector to monitor the transfection efficiency and the GFP signal was analysed within the mCherry positive population. The background fluorescence was estimated by using a crRNA-less, inactive LbCpf1 nuclease expression vector as negative control and was subtracted from each sample. Three parallel transfections were made for each case. Error bars show the mean ± standard deviation of percentages measured in n=3 independent transfections.

**Supplementary Figure S18. Base preference of wild type and RR mutant nucleases at PAM position -3**

Ratios of activities of the four nucleases averaged over targets in which the nucleotides have been varied systematically at PAM position -3 (TNTC). Green: thymidine, purple: cytosine, orange: adenine, blue: guanine.

**
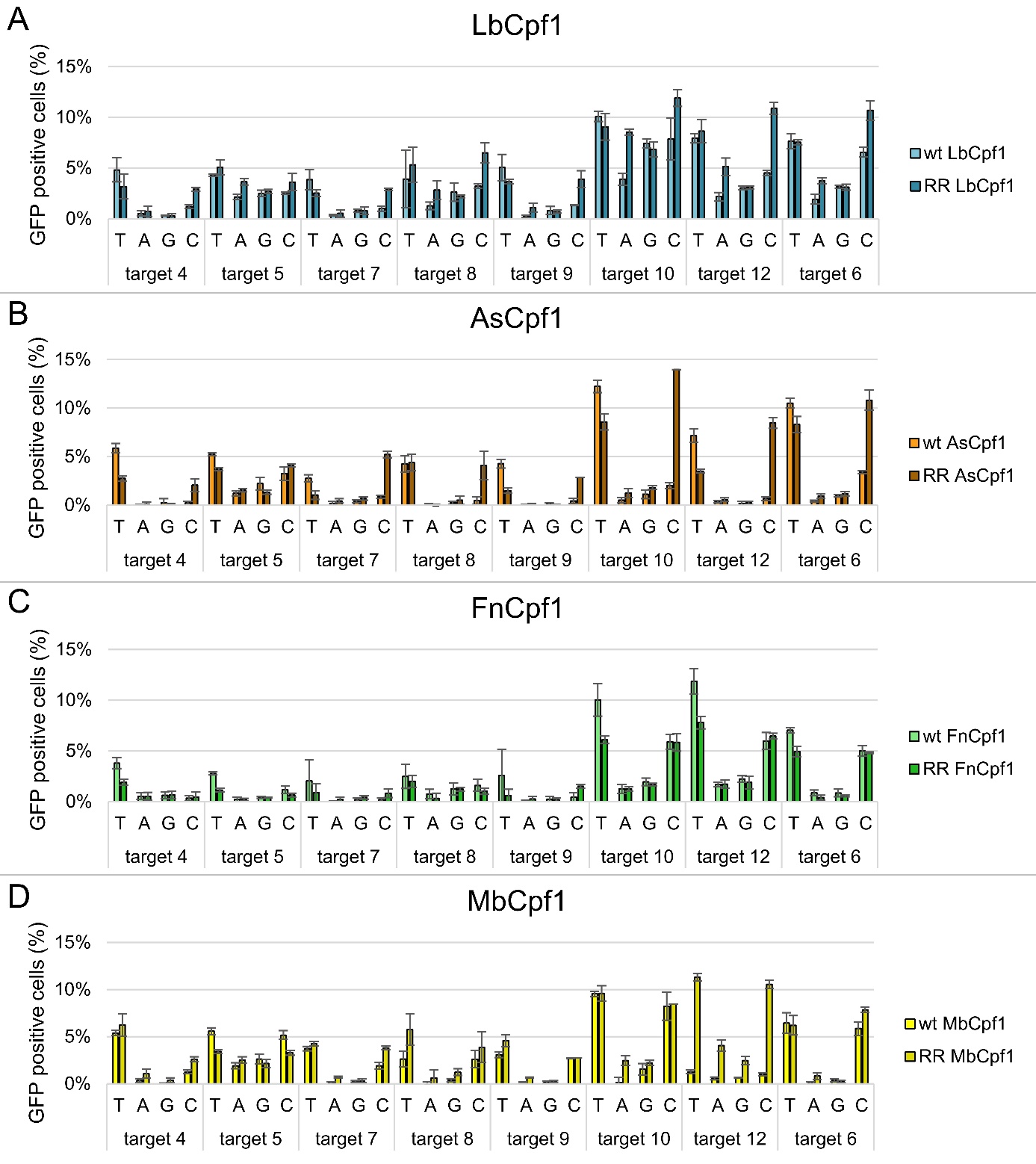
**

**Supplementary Figure S19. Detailed results of wild type and RR mutant nuclease activity on targets with TTNC PAM sequence**

We compared the activity of wild type and RR mutant Cpf1 nucleases on targets with TTNC PAM sequence in the GFxFP assay. Percentages of GFP positive cells counted above the background level resulting from the action of **A:** LbCpf1 (blue), **B:** AsCpf1 (orange), **C:** FnCpf1 (green), **D:** MbCpf1 (yellow) are shown. The target vectors along with the corresponding nuclease vectors were transfected into N2a cells and GFP positive cells were counted two days after transfection. All samples were also cotransfected with an mCherry expression vector to monitor the transfection efficiency and the GFP signal was analysed within the mCherry positive population. The background fluorescence was estimated by using a crRNA-less, inactive LbCpf1 nuclease expression vector as negative control and was subtracted from each sample. Three parallel transfections were made for each case. Error bars show the mean ± standard deviation of percentages measured in n=3 independent transfections.

**Supplementary Figure S20. Base preference of wild type and RR mutant nucleases at PAM position -2**

Ratios of activities of the four nucleases averaged over targets in which the nucleotides have been varied systematically at PAM position -2 (TTNC). Green: thymidine, purple: cytosine, orange: adenine, blue: guanine.


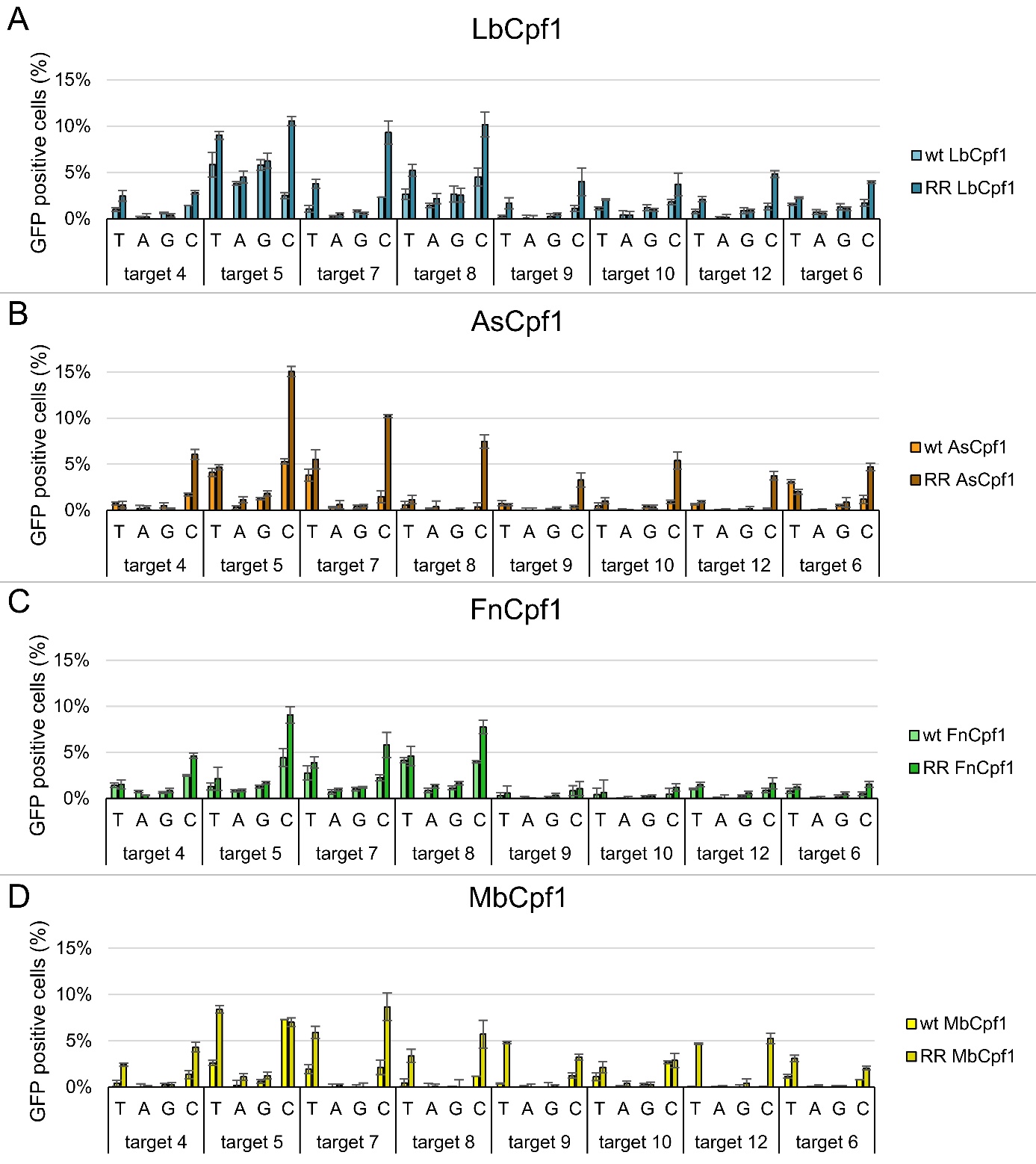


**Supplementary Figure S21. Detailed results of wild type and RR mutant nuclease activity on targets with TCNC PAM sequence**

We compared the activity of wild type and RR mutant Cpf1 nucleases on targets with TCNC PAM sequence in the GFxFP assay. Percentages of GFP positive cells counted above the background level resulting from the action of **A:** LbCpf1 (blue), **B:** AsCpf1 (orange), **C:** FnCpf1 (green), **D:** MbCpf1 (yellow) are shown. The target vectors along with the corresponding nuclease vectors were transfected into N2a cells and GFP positive cells were counted two days after transfection. All samples were also cotransfected with an mCherry expression vector to monitor the transfection efficiency and the GFP signal was analysed within the mCherry positive population. The background fluorescence was estimated by using a crRNA-less, inactive LbCpf1 nuclease expression vector as negative control and was subtracted from each sample. Three parallel transfections were made for each case. Error bars show the mean ± standard deviation of percentages measured in n=3 independent transfections.

**
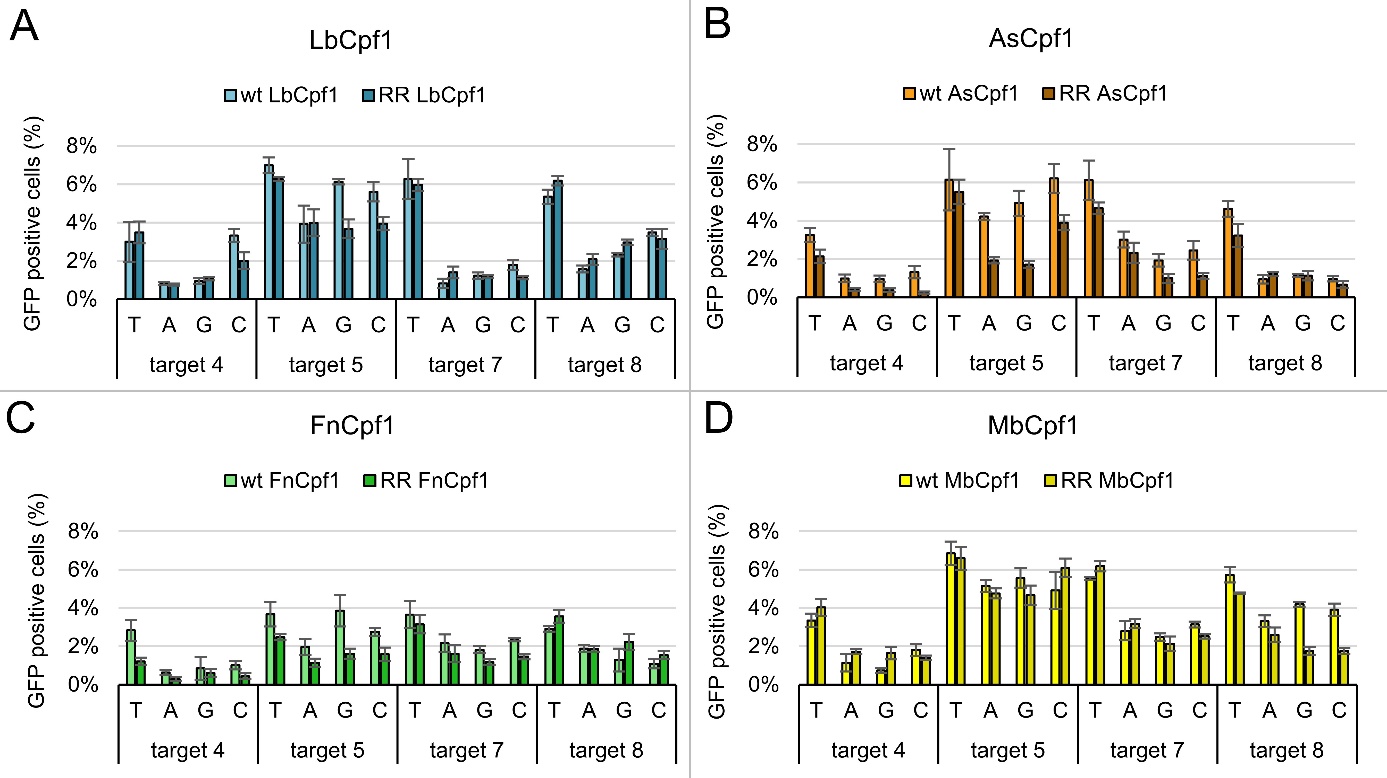
**

**Supplementary Figure S22. Detailed results of wild type and RR mutant nuclease activity on targets with NTTC PAM sequence**

We compared the activity of wild type and RR mutant Cpf1 nucleases on targets with NTTC PAM sequence in the GFxFP assay. Percentages of GFP positive cells counted above the background level resulting from the action of **A:** LbCpf1 (blue), **B:** AsCpf1 (orange), **C:** FnCpf1 (green), **D:** MbCpf1 (yellow) are shown. The target vectors along with the corresponding nuclease vectors were transfected into N2a cells and GFP positive cells were counted two days after transfection. All samples were also cotransfected with an mCherry expression vector to monitor the transfection efficiency and the GFP signal was analysed within the mCherry positive population. The background fluorescence was estimated by using a crRNA-less, inactive LbCpf1 nuclease expression vector as negative control and was subtracted from each sample. Three parallel transfections were made for each case. Error bars show the mean ± standard deviation of percentages measured in n=3 independent transfections.

**Supplementary Figure S23. Base preference of wild type and RR mutant nucleases at PAM position -4**

Ratios of activities of the four nucleases averaged over targets in which the nucleotides have been varied systematically at PAM position -4 (NTTC). Green: thymidine, purple: cytosine, orange: adenine, blue: guanine.

**Supplementary Tables**

**Supplementary Table S1. List of the oligonucleotides used for spacer cloning into the pTE4396, pTE4398, pTE4495 pTE4497, pTE4561, pTE4560, pTE4938, pTE3179, pTE3330, pTE3329, pTE3327, pTE3328, pTE3331, pTE3333, pTE3334 or pTE3336 vectors.** The matching targets, the potential cleavage positions in the ORF (when applicable) and the sequence of the oligonucleotides that form the corresponding linkers are listed. Addgene numbers for the vectors are: #74042 (pTE4398) (19), #74041 (pTE4396) (19), #80338 (pTE4495), #80339 (pTE4497), #107525 (pTE4561), #107526 (pTE4560), #107527 (pTE4938), #107528 (pTE3179), #107534 (pTE3330), #107538 (pTE3329), #107535 (pTE3327), #107539 (pTE3328), #107536 (pTE3331), #107540 (pTE3333), #107537 (pTE3334), #107541 (pTE3336).

| **Target** | **Potential genomic cleavage position** | **Oligonucleotide used*** |
| --- | --- | --- |
| **1** | mouse Prion protein ORF 5^th^ bp | *AAAA*GGTTCGCCATGATGACTGATCTG |
|  |  | *AGAT*CAGATCAGTCATCATGGCGAACC |
| **2** | mouse Prion protein ORF 55^th^ bp | *AAAA*GCCGACATCAGTCCACATAGTCA |
|  |  | *AGAT*TGACTATGTGGACTGATGTCGGC |
| **3** | mouse Prion protein ORF 58^th^ bp | *AAAA*TGTCGGCCTCTGCAAAAAGCGGC |
|  |  | *AGAT*GCCGCTTTTTGCAGAGGCCGACA |
| **4** | mouse Prion protein ORF 287^th^ bp | *AAAA*CATAATCAGTGGAACAAGCCCAG |
|  |  | *AGAT*CTGGGCTTGTTCCACTGATTATG |
| **5** | - | *AAAA*TGATTATGGGTACCCCCTCCTTG |
|  |  | *AGAT*CAAGGAGGGGGTACCCATAATCA |
| **6** | mouse Prion protein ORF 726^th^ bp | *AAAA*AGGATGACAGGAGGGGAGGA |
|  |  | *AGAT*TCCTCCCCTCCTGTCATCCT |
| **7 or PRND 1** | mouse Doppel protein ORF 3^th^ bp | *AAAA*TTCTTCATGGTGAATCTGCAAGG |
|  |  | *AGAT*CCTTGCAGATTCACCATGAAGAA |
| **8** | - | *AAAA*GATTCACCATGAAGAACCGGCTG |
|  |  | *AGAT*CAGCCGGTTCTTCATGGTGAATC |
| **9** | - | *AAAA*GTGACTATGTGGACTGATGTCGG |
|  |  | *AGAT*CCGACATCAGTCCACATAGTCAC |
| **10** | mouse Prion protein ORF 292^th^ bp | *AAAA*GTGGAACAAGCCCAGCAAAC |
|  |  | *AGAT*GTTTGCTGGGCTTGTTCCAC |
| **11** | mouse Prion protein ORF 432^th^ bp | *AAAA*GGGAGGACCGCTACTACCGT |
|  |  | *AGAT*ACGGTAGTAGCGGTCCTCCC |
| **12** | mouse Prion protein ORF 47^th^ bp | *AAAA*TGGACTGATGTCGGCCTCTG |
|  |  | *AGAT*CAGAGGCCGACATCAGTCCA |
| **13** | mouse Prion protein ORF 439^th^ bp | *AAAA*GCGGTCCTCCCAGTCGTTGC |
|  |  | *AGAT*GCAACGACTGGGAGGACCGC |
| **14** | DNMT 1.3[^1^](#_ENREF_1) | *AAAA*GAGTAACAGACATGGACCATCAG |
|  |  | *AGAT*CTGATGGTCCATGTCTGTTACTC |
| **PRND 2** | mouse Doppel protein ORF 67^th^ bp | *AAAA*CTCCACGGTCAAGGCAAGGGGCA |
|  |  | *AGAT*TGCCCCTTGCCTTGACCGTGGAG |
| **PRND 3** | mouse Doppel protein ORF 438^th^ bp | *AAAA*CCAAGCACTGCGATTTCTGGCTG |
|  |  | *AGAT*CAGCCAGAAATCGCAGTGCTTGG |
| **PRND 4** | mouse Doppel protein ORF 468^th^ bp | *AAAA*AGCGCAGCTCCCCTTTCCAGCCA |
|  |  | *AGAT*TGGCTGGAAAGGGGAGCTGCGCT |
| **PRND 5** | mouse Doppel protein ORF 519^th^ bp | *AAAA*GTTTCGTTTGGTTCATTGTGAAG |
|  |  | *AGAT*CTTCACAATGAACCAAACGAAAC |
| **PRND 6** | mouse Doppel protein ORF 537^th^ bp | *AAAA*CTTTACTTCACAATGAACCAAAC |
|  |  | *AGAT*GTTTGGTTCATTGTGAAGTAAAG |
| **GFP target 1** | GFP ORF in HEK-GFP cells | *AAAA*GGTCGAGCTGGACGGCGACG |
|  |  | *AGAT*CGTCGCCGTCCAGCTCGACC |
| **GFP target 2** | GFP ORF in HEK-GFP cells | *AAAA*AGCACCCAGTCCGCCCTGAG |
|  |  | *AGAT*CTCAGGGCGGACTGGGTGCT |
| **GFP target 3** | GFP ORF in HEK-GFP cells | *AAAA*GCTAGTGCTGGTACAGTGAG |
|  |  | *AGAT*CTCACTGTACCAGCACTAGC |
| **GFP target 4** | GFP ORF in HEK-GFP cells | *AGAT*CCGTAGGTGGCATCGCCCTC |
|  |  | *AAAA*GAGGGCGATGCCACCTACGG |
| **GFP target 5** | GFP ORF in HEK-GFP cells | *AGAT*CCGGTGGTGCAGATGAACTT |
|  |  | *AAAA*AAGTTCATCTGCACCACCGG |
| **GFP target 6** | GFP ORF in HEK-GFP cells | *AGAT*AGCCGCTACCCCGACCACAT |
|  |  | *AAAA*ATGTGGTCGGGGTAGCGGCT |
| **GFP target 7** | GFP ORF in HEK-GFP cells | *AGAT*AAGAAGTCGTGCTGCTTCAT |
|  |  | *AAAA*ATGAAGCAGCACGACTTCTT |
| **GFP target 8** | GFP ORF in HEK-GFP cells | *AGAT*GGGCATGGCGGACTTGAAGA |
|  |  | *AAAA*TCTTCAAGTCCGCCATGCCC |
| **GFP target 9** | GFP ORF in HEK-GFP cells | *AGAT*TTCAAGTCCGCCATGCCCGA |
|  |  | *AAAA*TCGGGCATGGCGGACTTGAA |
| **GFP target 10** | GFP ORF in HEK-GFP cells | *AGAT*AAGTCCGCCATGCCCGAAGG |
|  |  | *AAAA*CCTTCGGGCATGGCGGACTT |
| **GFP target 11** | GFP ORF in HEK-GFP cells | *AGAT*AAGAAGATGGTGCGCTCCTG |
|  |  | *AAAA*CAGGAGCGCACCATCTTCTT |
| **GFP target 12** | GFP ORF in HEK-GFP cells | *AGAT*TAGTTGCCGTCGTCCTTGAA |
|  |  | *AAAA*TTCAAGGACGACGGCAACTA |
| **GFP target 13** | GFP ORF in HEK-GFP cells | *AGAT*TTCAAGGACGACGGCAACTA |
|  |  | *AAAA*TAGTTGCCGTCGTCCTTGAA |
| **GFP target 14** | GFP ORF in HEK-GFP cells | *AGAT*AAGGACGACGGCAACTACAA |
|  |  | *AAAA*TTGTAGTTGCCGTCGTCCTT |
| **GFP target 15** | GFP ORF in HEK-GFP cells | *AGAT*ACCGGGGTGGTGCCCATCCT |
|  |  | *AAAA*AGGATGGGCACCACCCCGGT |
| **GFP target 16** | GFP ORF in HEK-GFP cells | *AGAT*AGCGTGTCCGGCGAGGGCGA |
|  |  | *AAAA*TCGCCCTCGCCGGACACGCT |
| **GFP target 17** | GFP ORF in HEK-GFP cells | *AGAT*ATCTGCACCACCGGCAAGCT |
|  |  | *AAAA*AGCTTGCCGGTGGTGCAGAT |
| **GFP target 18** | GFP ORF in HEK-GFP cells | *AGAT*CCGTCGTCCTTGAAGAAGAT |
|  |  | *AAAA*ATCTTCTTCAAGGACGACGG |
| **GFP target 19** | GFP ORF in HEK-GFP cells | *AGAT*TCATGGCCGACAAGCAGAAG |
|  |  | *AAAA*CTTCTGCTTGTCGGCCATGA |
| **GFP target 20** | GFP ORF in HEK-GFP cells | *AGAT*ATGGCCGACAAGCAGAAGAA |
|  |  | *AAAA*TTCTTCTGCTTGTCGGCCAT |
| **GFP target 21** | GFP ORF in HEK-GFP cells | *AGAT*CACCACCGGCAAGCTGCCCG |
|  |  | *AAAA*CGGGCAGCTTGCCGGTGGTG |
| **GFP target 22** | GFP ORF in HEK-GFP cells | *AGAT*CTTGTCGGCCATGATATAGA |
|  |  | *AAAA*TCTATATCATGGCCGACAAG |
| **GFP target 23** | GFP ORF in HEK-GFP cells | *AGAT*TATCATGGCCGACAAGCAGA |
|  |  | *AAAA*TCTGCTTGTCGGCCATGATA |
| **GFP target 24** | GFP ORF in HEK-GFP cells | *AGAT*CCGGGGTGGTGCCCATCCTG |
|  |  | *AAAA*CAGGATGGGCACCACCCCGG |
| **GFP target 25** | GFP ORF in HEK-GFP cells | *AGAT*TCTGCACCACCGGCAAGCTG |
|  |  | *AAAA*CAGCTTGCCGGTGGTGCAGA |
| **GFP target 26** | GFP ORF in HEK-GFP cells | *AGAT*GGCATGGCGGACTTGAAGAA |
|  |  | *AAAA*TTCTTCAAGTCCGCCATGCC |
| **GFP target 27** | GFP ORF in HEK-GFP cells | *AGAT*AGGGCGACACCCTGGTGAAC |
|  |  | *AAAA*GTTCACCAGGGTGTCGCCCT |
| **GFP target 28** | GFP ORF in HEK-GFP cells | *AGAT*GCGAGGGCGAGGGCGATGCC |
|  |  | *AAAA*GGCATCGCCCTCGCCCTCGC |
| **GFP target 29** | GFP ORF in HEK-GFP cells | *AGAT*GGAGCGCACCATCTTCTTCA |
|  |  | *AAAA*TGAAGAAGATGGTGCGCTCC |
| **GFP target 30** | GFP ORF in HEK-GFP cells | *AGAT*GCTTGTGCCCCAGGATGTTG |
|  |  | *AAAA*CAACATCCTGGGGCACAAGC |
| **GFP target 31** | GFP ORF in HEK-GFP cells | *AGAT*CCACAACATCGAGGACGGCA |
|  |  | *AAAA*TGCCGTCCTCGATGTTGTGG |
| **FANCF target 1** | FANCF amplicon 2 – target 1 | *AGAT*TGTGGCGAAAGTAAAAGTAT |
|  |  | *AAAA*ATACTTTTACTTTCGCCACA |
| **FANCF target 2** | FANCF amplicon 2 – target 2 | *AGAT*AGTTGCCCAGAGTCAAGGAA |
|  |  | *AAAA*TTCCTTGACTCTGGGCAACT |
| **FANCF target 3** | FANCF amplicon 2 – target 3 | *AGAT*TCCGTGTTCCTTGACTCTGG |
|  |  | *AAAA*CCAGAGTCAAGGAACACGGA |
| **FANCF target 4** | FANCF amplicon 2 – target 4 | *AGAT*GACCAATAGCATTGCAGAGA |
|  |  | *AAAA*TCTCTGCAATGCTATTGGTC |
| **FANCF target 5** | FANCF amplicon 1 – target 5 | *AGAT*GCGGATGTTCCAATCAGTAC |
|  |  | *AAAA*GTACTGATTGGAACATCCGC |
| **FANCF target 6** | FANCF amplicon 1 – target 6 | *AGAT*ACCTTGGAGACGGCGACTCT |
|  |  | AAAAAGAGTCGCCGTCTCCAAGGT |
| **FANCF target 7** | FANCF amplicon 2 – target 7 | *AGAT*CGAGCTTCTGGCGGTCTCAA |
|  |  | *AAAA*TTGAGACCGCCAGAAGCTCG |
| **FANCF target 8** | FANCF amplicon 2 – target 8 | *AGAT*GTCGGCATGGCCCCATTCGC |
|  |  | *AAAA*GCGAATGGGGCCATGCCGAC |
| **FANCF target 9** | FANCF amplicon 9 – target 9 | *AGAT*GGCGGGGTCCAGTTCCGGGA |
|  |  | *AAAA*TCCCGGAACTGGACCCCGCC |
| **FANCF target 10** | FANCF amplicon 1 – target 10 | *AGAT*CCTAAAACTAGAATCTGTGT |
|  |  | *AAAA*ACACAGATTCTAGTTTTAGG |
| **FANCF target 11** | FANCF amplicon 1 – target 11 | *AGAT*AAAAACCTCAACACAGATTC |
|  |  | *AAAA*GAATCTGTGTTGAGGTTTTT |
| **FANCF target 12** | FANCF amplicon 1 – target 12 | *AGAT*TGAGCTTTGCCTAAAACTAG |
|  |  | *AAAA*CTAGTTTTAGGCAAAGCTCA |
| **FANCF target 13** | FANCF amplicon 1 – target 13 | *AGAT*GAAATATCCTTAAGTAGAAA |
|  |  | *AAAA*TTTCTACTTAAGGATATTTC |
| **FANCF target 14** | FANCF amplicon 1 – target 14 | *AGAT*GGCAAAGCTCAGAAAATTTC |
|  |  | *AAAA*GAAATTTTCTGAGCTTTGCC |
| **FANCF target 15** | FANCF amplicon 1 – target 15 | *AGAT*GCTTTGGAAATATCCTTAAG |
|  |  | *AAAA*CTTAAGGATATTTCCAAAGC |
| **FANCF target 16** | FANCF amplicon 1 – target 16 | *AGAT*TACTTAAGGATATTTCCAAA |
|  |  | *AAAA*TTTGGAAATATCCTTAAGTA |
| **FANCF target 17** | FANCF amplicon 1 – target 17 | *AGAT*CAAAGCGAAAGGAAGCGCGG |
|  |  | *AAAA*CCGCGCTTCCTTTCGCTTTG |
| **FANCF target 18** | FANCF amplicon 1 – target 18 | *AGAT*CGGAACCACGTGCGAGATGA |
|  |  | *AAAA*TCATCTCGCACGTGGTTCCG |
| **VEGFA target 19** | VEGFA amplicon – target 19 | *AGAT*GGACTGGAGTTGCTTCATGT |
|  |  | *AAAA*ACATGAAGCAACTCCAGTCC |
| **VEGFA target 20** | VEGFA amplicon – target 20 | *AGAT*TGACCTCCCAAACAGCTACA |
|  |  | *AAAA*TGTAGCTGTTTGGGAGGTCA |
| **VEGFA target 21** | VEGFA amplicon – target 21 | *AGAT*CTCCTGGACCCCCTATTTCT |
|  |  | *AAAA*AGAAATAGGGGGTCCAGGAG |
| **VEGFA target 22** | VEGFA amplicon – target 22 | *AGAT*GGAGGTCAGAAATAGGGGGT |
|  |  | *AAAA*ACCCCCTATTTCTGACCTCC |
| **VEGFA target 23** | VEGFA amplicon – target 23 | *AGAT*CAAAGCCCATTCCCTCTTTA |
|  |  | *AAAA*TAAAGAGGGAATGGGCTTTG |
| **VEGFA target 24** | VEGFA amplicon – target 24 | *AGAT*GCCAGAGCCGGGGTGTGCAG |
|  |  | *AAAA*CTGCACACCCCGGCTCTGGC |
| **VEGFA target 25** | VEGFA amplicon – target 25 | *AGAT*CTAGGAATATTGAAGGGGGC |
|  |  | *AAAA*GCCCCCTTCAATATTCCTAG |

*: The overhangs of the DNA linkers are marked in italics.

**Supplementary Table S2. Oligonucleotides used for nuclease target cloning into the pWN10042 (pGF-ori-FP) vector.**

The names of the targets and the sequences of the corresponding oligonucleotides used are listed. Two targets are generally incorporated into one oligonucleotide linker. Addgene number of pWN10042: #89052 (19).

| **Target** | **Oligonucleotide used*** |
| --- | --- |
| **1** | *GATC*GCCTTTGCAGATCAGTCATCATGGCGAACCTTGGAAAAATTGGAGACGAAGCTTCGTCTCCGATCC |
|  | *AATT*GGATCGGAGACGAAGCTTCGTCTCCAATTTTTCCAAGGTTCGCCATGATGACTGATCTGCAAAGGC |
| **2** | *GATC*GCCCTCTTTGTGACTATGTGGACTGATGTCGGCAAATTGGAGACGGTCGACCGTCTCCGATC |
|  | *AATT*GATCGGAGACGGTCGACCGTCTCCAATTTGCCGACATCAGTCCACATAGTCACAAAGAGGGC |
| **3** | *GATC*TTTATGTCGGCCTCTGCAAAAAGCGGCCAAAGCAATTGGAGACGCTGCAGCGTCTCCGATC |
|  | *AATT*GATCGGAGACGCTGCAGCGTCTCCAATTGCTTTGGCCGCTTTTTGCAGAGGCCGACATAAA |
| **4, 5, 7, 8** | *GATC*TTTCCCTTGCAGATTCACCATGAAGAACCGGCTGGAAATTGGAGACGTTCGAATCGTCTCCGATC |
|  | *AATT*GATCGGAGACGATTCGAACGTCTCCAATTTCCAGCCGGTTCTTCATGGTGAATCTGCAAGGGAAA |
| **6** | *GATC*TTTTCTCCTCCCCTCCTGTCATCCTCAAAGATATCTTTCGGTCGAGCTGGACGGCGACGTA |
|  | *AATT*TACGTCGCCGTCCAGCTCGACCGAAAGATATCTTTGAGGATGACAGGAGGGGAGGAGAAAA |
| **14** | *GATC*CACTCCCGTCTTCGATATCGAAGACCCTTTCCTGATGGTCCATGTCTGTTACTCGGAGACGAAGCTTCGTCTCGTTTCG |
|  | *AATT*CGAAACGAGACGAAGCTTCGTCTCCGAGTAACAGACATGGACCATCAGGAAAGGGTCTTCGATATCGAAGACGGGAGTG |
| **4, 5, 7, 8**  **PAM 1.1** | *GATC*TTCCAAGGAGGGGGTACCCATAATCAGTGGAACAAGCCCAGCAACCCGGGTTCCAGCCGGTTCTTCATGGTGAATCTGCAAGGG |
|  | *AATT*CCCTTGCAGATTCACCATGAAGAACCGGCTGGAACCCGGGTTGCTGGGCTTGTTCCACTGATTATGGGTACCCCCTCCTTGGAA |
| **4, 5, 7, 8**  **PAM 1.2** | *GATC*GTTCCAAGGAGGGGGTACCCATAATCAGTGGAACAAGCCCAGCAAGCCGGCTTCCAGCCGGTTCTTCATGGTGAATCTGCAAGGGAAC |
|  | *AATT*GTTCCCTTGCAGATTCACCATGAAGAACCGGCTGGAAGCCGGCTTGCTGGGCTTGTTCCACTGATTATGGGTACCCCCTCCTTGGAAC |
| **4, 5, 7, 8**  **PAM 1.3** | *GATC*ATTCCAAGGAGGGGGTACCCATAATCAGTGGAACAAGCCCAGCAATCCGGATTCCAGCCGGTTCTTCATGGTGAATCTGCAAGGGAAG |
|  | *AATT*CTTCCCTTGCAGATTCACCATGAAGAACCGGCTGGAATCCGGATTGCTGGGCTTGTTCCACTGATTATGGGTACCCCCTCCTTGGAAT |
| **4, 5, 7, 8**  **PAM 2.1** | *GATC*TATCCAAGGAGGGGGTACCCATAATCAGTGGAACAAGCCCAGCATAGATATCTATCCAGCCGGTTCTTCATGGTGAATCTGCAAGGGAT |
|  | *AATT*ATCCCTTGCAGATTCACCATGAAGAACCGGCTGGATAGATATCTATGCTGGGCTTGTTCCACTGATTATGGGTACCCCCTCCTTGGATA |
| **4, 5, 7, 8**  **PAM 2.2** | *GATC*TGTCCAAGGAGGGGGTACCCATAATCAGTGGAACAAGCCCAGCACAGATATCTGTCCAGCCGGTTCTTCATGGTGAATCTGCAAGGGAC |
|  | *AATT*GTCCCTTGCAGATTCACCATGAAGAACCGGCTGGACAGATATCTGTGCTGGGCTTGTTCCACTGATTATGGGTACCCCCTCCTTGGACA |
| **4, 5, 7, 8**  **PAM 2.3** | *GATC*TCTCCAAGGAGGGGGTACCCATAATCAGTGGAACAAGCCCAGCAGAGATATCTCTCCAGCCGGTTCTTCATGGTGAATCTGCAAGGGAG |
|  | *AATT*CTCCCTTGCAGATTCACCATGAAGAACCGGCTGGAGAGATATCTCTGCTGGGCTTGTTCCACTGATTATGGGTACCCCCTCCTTGGAGA |
| **4, 5, 7, 8**  **PAM 3.1** | *GATC*TTACCAAGGAGGGGGTACCCATAATCAGTGGAACAAGCCCAGCTAAGATATCTTACCAGCCGGTTCTTCATGGTGAATCTGCAAGGGT |
|  | *AATT*ACCCTTGCAGATTCACCATGAAGAACCGGCTGGTAAGATATCTTAGCTGGGCTTGTTCCACTGATTATGGGTACCCCCTCCTTGGTAA |
| **4, 5, 7, 8**  **PAM 3.2** | *GATC*TTGCCAAGGAGGGGGTACCCATAATCAGTGGAACAAGCCCAGCCAAGATATCTTGCCAGCCGGTTCTTCATGGTGAATCTGCAAGGGC |
|  | *AATT*GCCCTTGCAGATTCACCATGAAGAACCGGCTGGCAAGATATCTTGGCTGGGCTTGTTCCACTGATTATGGGTACCCCCTCCTTGGCAA |
| **4, 5, 7, 8**  **PAM 3.3** | *GATC*TTCCCAAGGAGGGGGTACCCATAATCAGTGGAACAAGCCCAGCGAAGATATCTTCCCAGCCGGTTCTTCATGGTGAATCTGCAAGGGG |
|  | *AATT*CCCCTTGCAGATTCACCATGAAGAACCGGCTGGGAAGATATCTTCGCTGGGCTTGTTCCACTGATTATGGGTACCCCCTCCTTGGGAA |
| **4, 5, 7, 8**  **PAM 4.1** | *GATC*TTTTCAAGGAGGGGGTACCCATAATCAGTGGAACAAGCCCAGAAAAGATATCTTTTCAGCCGGTTCTTCATGGTGAATCTGCAAGGAA |
|  | *AATT*TTCCTTGCAGATTCACCATGAAGAACCGGCTGAAAAGATATCTTTTCTGGGCTTGTTCCACTGATTATGGGTACCCCCTCCTTGAAAA |
| **4, 5, 7, 8**  **PAM 4.2** | *GATC*TTTACAAGGAGGGGGTACCCATAATCAGTGGAACAAGCCCAGTAAAGATATCTTTACAGCCGGTTCTTCATGGTGAATCTGCAAGGTA |
|  | *AATT*TACCTTGCAGATTCACCATGAAGAACCGGCTGTAAAGATATCTTTACTGGGCTTGTTCCACTGATTATGGGTACCCCCTCCTTGTAAA |
| **4, 5, 7, 8**  **PAM 4.3** | *GATC*TTTGCAAGGAGGGGGTACCCATAATCAGTGGAACAAGCCCAGGAAAGATATCTTTGCAGCCGGTTCTTCATGGTGAATCTGCAAGGCA |
|  | *AATT*TGCCTTGCAGATTCACCATGAAGAACCGGCTGCAAAGATATCTTTCCTGGGCTTGTTCCACTGATTATGGGTACCCCCTCCTTGCAAA |
| **9, 10 PAM 5.1** | *GATC*TTTCGTTTGCTGGGCTTGTTCCACCTCGAGTTTCACGGTAGTAGCGGTCCTCCC |
|  | *AATT*GGGAGGACCGCTACTACCGTGAAACTCGAGGTGGAACAAGCCCAGCAAACGAAA |
| **9, 10 PAM 5.2** | *GATC*ATTCGTTTGCTGGGCTTGTTCCACCTCGAGATTCACGGTAGTAGCGGTCCTCCC |
|  | *AATT*GGGAGGACCGCTACTACCGTGAATCTCGAGGTGGAACAAGCCCAGCAAACGAAT |
| **9, 10 PAM 5.3** | *GATC*GTTCGTTTGCTGGGCTTGTTCCACCTCGAGGTTCACGGTAGTAGCGGTCCTCCC |
|  | *AATT*GGGAGGACCGCTACTACCGTGAACCTCGAGGTGGAACAAGCCCAGCAAACGAAC |
| **9, 10 PAM 5.4** | *GATC*CTTCGTTTGCTGGGCTTGTTCCACCTCGAGCTTCACGGTAGTAGCGGTCCTCCC |
|  | *AATT*GGGAGGACCGCTACTACCGTGAAGCTCGAGGTGGAACAAGCCCAGCAAACGAAG |
| **1, 11 PAM 6.1** | *GATC*TTTCCAGATCAGTCATCATGGCGACTCGAGTTTCCAGAGGCCGACATCAGTCCA |
|  | *AATT*TGGACTGATGTCGGCCTCTGGAAACTCGAGTCGCCATGATGACTGATCTGGAAA |
| **1, 11 PAM 6.2** | *GATC*ATTCCAGATCAGTCATCATGGCGACTCGAGATTCCAGAGGCCGACATCAGTCCA |
|  | *AATT*TGGACTGATGTCGGCCTCTGGAATCTCGAGTCGCCATGATGACTGATCTGGAAT |
| **1, 11 PAM 6.3** | *GATC*GTTCCAGATCAGTCATCATGGCGACTCGAGGTTCCAGAGGCCGACATCAGTCCA |
|  | *AATT*TGGACTGATGTCGGCCTCTGGAACCTCGAGTCGCCATGATGACTGATCTGGAAC |
| **1, 11 PAM 6.4** | *GATC*CTTCCAGATCAGTCATCATGGCGACTCGAGCTTCCAGAGGCCGACATCAGTCCA |
|  | *AATT*TGGACTGATGTCGGCCTCTGGAAGCTCGAGTCGCCATGATGACTGATCTGGAAG |
| **6, 12 PAM 7.1** | *GATC*TTTCGCAACGACTGGGAGGACCGCCTCGAGTTTCTCCTCCCCTCCTGTCATCCT |
|  | *AATT*AGGATGACAGGAGGGGAGGAGAAACTCGAGGCGGTCCTCCCAGTCGTTGCGAAA |
| **6, 12 PAM 7.2** | *GATC*ATTCGCAACGACTGGGAGGACCGCCTCGAGATTCTCCTCCCCTCCTGTCATCCT |
|  | *AATT*AGGATGACAGGAGGGGAGGAGAATCTCGAGGCGGTCCTCCCAGTCGTTGCGAAT |
| **6, 12 PAM 7.3** | *GATC*GTTCGCAACGACTGGGAGGACCGCCTCGAGGTTCTCCTCCCCTCCTGTCATCCT |
|  | *AATT*AGGATGACAGGAGGGGAGGAGAACCTCGAGGCGGTCCTCCCAGTCGTTGCGAAC |
| **6, 12 PAM 7.4** | *GATC*CTTCGCAACGACTGGGAGGACCGCCTCGAGCTTCTCCTCCCCTCCTGTCATCCT |
|  | *AATT*AGGATGACAGGAGGGGAGGAGAAGCTCGAGGCGGTCCTCCCAGTCGTTGCGAAG |
| **2, 13 PAM 8.1** | *GATC*GCCCTCTTTGTGACTATGTGGACTGATGTCGGCAAATTGGAGACGGTCGACCGTCTCCGATC |
|  | *AATT*GATCGGAGACGGTCGACCGTCTCCAATTTGCCGACATCAGTCCACATAGTCACAAAGAGGGC |
| **2, 13 PAM 8.2** | GATCATTGTGACTATGTGGACTGATGTCGGCAAT |
|  | *AATT*ATTGCCGACATCAGTCCACATAGTCACAAT |
| **2, 13 PAM 8.3** | *GATC*GTTGTGACTATGTGGACTGATGTCGGCAAC |
|  | *AATT*GTTGCCGACATCAGTCCACATAGTCACAAC |
| **2, 13 PAM 8.4** | *GATC*CTTGTGACTATGTGGACTGATGTCGGCAAG |
|  | *AATT*CTTGCCGACATCAGTCCACATAGTCACAAG |
| **9, 10**  **PAM 9** | *GATC*TCTCGTTTGCTGGGCTTGTTCCACCTCGAGTCTCACGGTAGTAGCGGTCCTCCC |
|  | *AATT*GGGAGGACCGCTACTACCGTGAGACTCGAGGTGGAACAAGCCCAGCAAACGAGA |
| **9, 10**  **PAM 10** | *GATC*TTCCGTTTGCTGGGCTTGTTCCACCTCGAGTTCCACGGTAGTAGCGGTCCTCCC |
|  | *AATT*GGGAGGACCGCTACTACCGTGGAACTCGAGGTGGAACAAGCCCAGCAAACGGAA |
| **9, 10**  **PAM 11** | *GATC*TCCCGTTTGCTGGGCTTGTTCCACCTCGAGTCCCACGGTAGTAGCGGTCCTCCC |
|  | *AATT*GGGAGGACCGCTACTACCGTGGGACTCGAGGTGGAACAAGCCCAGCAAACGGGA |
| **12, 6**  **PAM 12** | *GATC*TCTCGCAACGACTGGGAGGACCGCCTCGAGTCTCTCCTCCCCTCCTGTCATCCT |
|  | *AATT*AGGATGACAGGAGGGGAGGAGAGACTCGAGGCGGTCCTCCCAGTCGTTGCGAGA |
| **12, 6**  **PAM 13** | *GATC*TTCCGCAACGACTGGGAGGACCGCCTCGAGTTCCTCCTCCCCTCCTGTCATCCT |
|  | *AATT*AGGATGACAGGAGGGGAGGAGGAACTCGAGGCGGTCCTCCCAGTCGTTGCGGAA |
| **12, 6**  **PAM 14** | *GATC*TCCCGCAACGACTGGGAGGACCGCCTCGAGTCCCTCCTCCCCTCCTGTCATCCT |
|  | *AATT*AGGATGACAGGAGGGGAGGAGGGACTCGAGGCGGTCCTCCCAGTCGTTGCGGGA |
| **4, 5, 7, 8**  **PAM 15** | *GATC*TCCCCAAGGAGGGGGTACCCATAATCAGTGGAACAAGCCCAGGGGAGATATCTCCCCAGCCGGTTCTTCATGGTGAATCTGCAAGGGGG |
|  | *AATT*CCCCCTTGCAGATTCACCATGAAGAACCGGCTGGGGAGATATCTCCCCTGGGCTTGTTCCACTGATTATGGGTACCCCCTCCTTGGGGA |

*: The overhangs of the DNA linkers are marked in italics.

**Supplementary Table S3. List of the oligonucleotides used for generating inactive Cpf1 mutants.** The name given to the inactive nuclease, the inactivating mutations and the sequence of the oligonucleotides used for PCR are listed.

| **Nuclease** | **Mutation** | **Oligonucleotide used** |
| --- | --- | --- |
| **LbCpf1 dead 1** | D832A | GCGGGACTCTGGGGTTCG |
|  |  | CGCGATGCCGATCACATAGGGGT |
|  |  | ATGTGATCGGCATCGCGAGGGGCGAGCGCAATCTG |
|  |  | AACCCCAGAGTCCCGCTCAGAAGAACTC |
| **LbCpf1 dead 2** | E925A | GCGGGACTCTGGGGTTCG |
|  |  | GGCCAGGGCGATCACGGCATCGTA |
|  |  | CGTGATCGCCCTGGCCGACCTGAACTCTGGC |
|  |  | AACCCCAGAGTCCCGCTCAGAAGAACTC |
| **AsCpf1 dead 1** | D908A | GCGGGACTCTGGGGTTCG |
|  |  | GGCGATGCCGATGATAGGTGTCTCGG |
|  |  | CACCTATCATCGGCATCGCCCGGGGCGAGAGAAACCTGATCT |
|  |  | AACCCCAGAGTCCCGCTCAGAAGAACTC |
| **AsCpf1 dead 2** | E993A | GCGGGACTCTGGGGTTCG |
|  |  | GGCCAGCACCACCACGGCCT |
|  |  | GGCCGTGGTGGTGCTGGCCAACCTGAATTTCGGCTTTAAGAGCAAGAGG |
|  |  | AACCCCAGAGTCCCGCTCAGAAGAACTC |
| **MbCpf1 dead 1** | D986A | GCGGGACTCTGGGGTTCG |
|  |  | TGAACGTGATCGGCATCGCCCGGGGCGAGAGACA |
|  |  | GGCGATGCCGATCACGTTCACCTCGTC |
|  |  | AACCCCAGAGTCCCGCTCAGAAGAACTC |
| **MbCpf1 dead 2** | E1080A | GCGGGACTCTGGGGTTCG |
|  |  | GCTAGCACCACGATGGCGTTGTACTTCAG |
|  |  | AACGCCATCGTGGTGCTAGCGGACCTGAATTTCGGC |
|  |  | AACCCCAGAGTCCCGCTCAGAAGAACTC |
| **FnCpf1 dead 1** | D917A | GCGGGACTCTGGGGTTCG |
|  |  | CCGCTCTCCTCGAGCAATGCTCAGGATGTGCAC |
|  |  | GTGCACATCCTGAGCATTGCTCGAGGAGAGCGG |
|  |  | AACCCCAGAGTCCCGCTCAGAAGAACTC |

**Supplementary Table S4. List of the oligonucleotides used for spacer cloning into the px330-U6-Chimeric_BB-CBh-hSpCas9 vector.** The matching target names, the potential cleavage positions in the ORF and the sequence of the oligonucleotides that form the corresponding linkers are listed. Addgene number of px330-U6-Chimeric_BB-CBh-hSpCas9 is #42230 (2).

| **Target** | **Potential cleavage position** | **Oligonucleotide used*** |
| --- | --- | --- |
| **PRND 1** | mouse Doppel protein +10 bp before ATG | *CACC*GTTCTTCATGGTGAATCTGCA |
|  |  | *AAAC*TGCAGATTCACCATGAAGAAC |
| **PRND 2a** | mouse Doppel protein ORF 70^th^ bp | *CACC*GTTATGCCCCTTGCCTTGACCG |
|  |  | *AAAC*CGGTCAAGGCAAGGGGCATAAC |
| **PRND 2b** | mouse Doppel protein ORF 77^th^ bp | *CACC*GCTCTCCACGGTCAAGGCAAG |
|  |  | *AAAC*CTTGCCTTGACCGTGGAGAGC |
| **PRND 3a** | mouse Doppel protein ORF 436^th^ bp | *CACC*GCCAGAAATCGCAGTGCTTGG |
|  |  | *AAAC*CCAAGCACTGCGATTTCTGGC |
| **PRND 3b** | mouse Doppel protein ORF 451^th^ bp | *CACC*GCAAGCACTGCGATTTCTGGC |
|  |  | *AAAC*GCCAGAAATCGCAGTGCTTGC |
| **PRND 5-6** | mouse Doppel protein ORF 519^h^ bp | *CACC*GTCTGCCTGCTGGGTTTCGTT |
|  |  | *AAAC*AACGAAACCCAGCAGGCAGAC |

*: The overhangs of the DNA linkers are marked in italics.

**Supplementary Table S5. List of the oligonucleotides used for generating RVR and RR Cpf1 mutants.** The name given to the mutant nuclease, the mutations and the sequence of the oligonucleotides used for PCR are listed.

| **Nuclease** | **Mutation** | **Oligonucleotide used** |
| --- | --- | --- |
| **LbCpf1 RVR** | G532R K538V Y542R | GCGGGACTCTGGGGTTCG |
|  |  | GGTGGCCCGCCGATCGGTCTCCACATCCTTGTCCCAGCCCCTCATGAACTGAGGGTTCTGAAA |
|  |  | AACCCCAGAGTCCCGCTCAGAAGAACTC |
|  |  | CAGTTCATGAGGGGCTGGGACAAGGATGTGGAGACCGATCGGCGGGCCACCATCCTGAGATAC |
| **MbCpf1 RVR** | N576R K582V N586R | GCGGGACTCTGGGGTTCG |
|  |  | CACGCCGAACCTATCCTTCTCCACGTTCAGATCCCAGCCCCTCAGCAGTGTTGGATTGCCAAA |
|  |  | AACCCCAGAGTCCCGCTCAGAAGAACTC |
|  |  | ACACTGCTGAGGGGCTGGGATCTGAACGTGGAGAAGGATAGGTTCGGCGTGATCCTGCAGAAG |
| **MbCpf1 RR** | N576R K637R | GCGGGACTCTGGGGTTCG |
|  |  | CTTGTTCAGATCCCAGCCCCTCAGCAGTGTTGGATTGCCAAA |
|  |  | AACCCCAGAGTCCCGCTCAGAAGAACTC |
|  |  | ACACTGCTGAGGGGCTGGGATCTGAACAAGGAGAAGGATAAT |
|  |  | GGAAAAGAACACCCGGGGGAACTGCTTCCTCACCTC |
|  |  | CAGTTCCCCCGGGTGTTCTTTTCCAAGGAGGCC |
| **FnCpf1 RVR** | N607R K613V N617R | GCGGGACTCTGGGGTTCG |
|  |  | GCTGTCCTGTCAGGCTCCACGTTCTTATCCCACCCCCTGGCTAGCGTACTATTTTCGAAGTTCAG |
|  |  | AACCCCAGAGTCCCGCTCAGAAGAACTC |
|  |  | AGTACGCTAGCCAGGGGGTGGGATAAGAACGTGGAGCCTGACAGGACAGCTATCCTGTTCATCAAG |
| **FnCpf1 RR** | N607R K671R | GCGGGACTCTGGGGTTCG |
|  |  | ATCCCACCCCCTGGCTAGCGTACTATTTTCGAAGTTCAG |
|  |  | AACCCCAGAGTCCCGCTCAGAAGAACTC |
|  |  | AGTACGCTAGCCAGGGGGTGGGATAAGAACAAGGA |
|  |  | GAAGAACACCCTAGGCAGCATCTTATTTGCGC |
|  |  | ATGCTGCCTAGGGTGTTCTTCAGCGCCAAGAG |

**Supplementary Table S6 List of plasmids deposited at Addgene**

| **Addgene Number** | **Construct Name** | **Description** |
| --- | --- | --- |
| #80338 | pTE4495 | Expresses MbCpf1 nuclease and Mb crRNA. |
| #80339 | pTE4497 | Expresses FnCpf1 nuclease and Fn crRNA. |
| #80443 | WN10150 | Expresses dead1 (D908A) AsCpf1. |
| #88903 | pTE4565 | Expresses dead1 MbCpf1 nuclease. |
| #88904 | pTE4566 | Expresses Mb crRNA and dead1 MbCpf1 nuclease. |
| #107557 | pTE4567 | Expresses dead2 MbCpf1 nuclease. |
| #88905 | pTE4889 | Expresses Fn crRNA and dead1 FnCpf1 nuclease. |
| #107555 | pTE4999 | Expresses dead1 FnCpf1 nuclease. |
| #107525 | pTE4561 | Expresses Lb crRNA and mCherry. |
| #107526 | pTE4560 | Expresses As crRNA and mCherry. |
| #107527 | pTE4938 | Expresses Fn crRNA and mCherry. |
| #107528 | pTE3179 | Expresses Mb crRNA and mCherry. |
| #107529 | pTE3320 | Expresses LbCpf1 RVR mutant. |
| #107530 | pTE3314 | Expresses FnCpf1 RVR mutant. |
| #107531 | pTE3317 | Expresses MbCpf1 RVR mutant. |
| #107532 | pTE3316 | Expresses FnCpf1 RR mutant. |
| #107533 | pTE3319 | Expresses MbCpf1 RR mutant. |
| #107534 | pTE3330 | Expresses Lb crRNA and LbCpf1 RVR mutant. |
| #107535 | pTE3327 | Expresses As crRNA and AsCpf1 RVR mutant. |
| #107536 | pTE3331 | Expresses Fn crRNA and FnCpf1 RVR mutant. |
| #107537 | pTE3334 | Expresses Mb crRNA and MbCpf1 RVR mutant. |
| #107538 | pTE3329 | Expresses Lb crRNA and LbCpf1 RR mutant. |
| #107539 | pTE3328 | Expresses As crRNA and AsCpf1 RR mutant. |
| #107540 | pTE3333 | Expresses Fn crRNA and FnCpf1 RR mutant. |
| #107541 | pTE3336 | Expresses Mb crRNA and MbCpf1 RR mutant. |

**Supplementary Table S7. 1^st^ step PCR primers used to amplify FANCF or VEGFA amplicons from genomic DNA of human HEK293 cells.** Illumina index adapters are marked by bold grey (i5) and bold black (i7), and matching primer sequences are marked by italic.

| **amplicon** | **primer 1** | **primer 2** |
| --- | --- | --- |
| FANCF amplicon 1 - FWD | **TCGTCGGCAGCGTCAGATGTGTATAAGAGACAG***GGTGCTGACGTAGGTAGTGC* | **GTCTCGTGGGCTCGGAGATGTGTATAAGAGACAG***GTCACAGTATGTCTCTGGCGT* |
| FANCF amplicon 1 - REV | **GTCTCGTGGGCTCGGAGATGTGTATAAGAGACAG***GGTGCTGACGTAGGTAGTGC* | **TCGTCGGCAGCGTCAGATGTGTATAAGAGACAG***GTCACAGTATGTCTCTGGCGT* |
| FANCF amplicon 2 - FWD | **TCGTCGGCAGCGTCAGATGTGTATAAGAGACAG***GGGCCGGGAAAGAGTTGCTG* | **GTCTCGTGGGCTCGGAGATGTGTATAAGAGACAG***GCCCTACATCTGCTCTCCCTCC* |
| FANCF amplicon 1 - REV | **GTCTCGTGGGCTCGGAGATGTGTATAAGAGACAG***GGGCCGGGAAAGAGTTGCTG* | **TCGTCGGCAGCGTCAGATGTGTATAAGAGACAG***GCCCTACATCTGCTCTCCCTCC* |
| VEGFA amplicon - FWD | **TCGTCGGCAGCGTCAGATGTGTATAAGAGACAG***TCCAGATGGCACATTGTCAG* | **GTCTCGTGGGCTCGGAGATGTGTATAAGAGACAG***AGGGAGCAGGAAAGTGAGGT* |
| VEGFA amplicon - REV | **GTCTCGTGGGCTCGGAGATGTGTATAAGAGACAG***TCCAGATGGCACATTGTCAG* | **TCGTCGGCAGCGTCAGATGTGTATAAGAGACAG***AGGGAGCAGGAAAGTGAGGT* |

**Supplemental Methods and Materials**

**Plasmid construction**

Information for the construction and sequences of the following plasmids can be found in reference (19): pWN10042 plasmid (Addgene number: #89052), pcDNA3-mCherry (transfection control), pLbCpf1-DNMT1.3 (target 9), pAsCpf1-DNMT1.3 (target 9), pLbCpf1-crRNA (pTE4398, Addgene number: #74042), pAsCpf1-crRNA (pTE4396, Addgene number: #74041), LbCpf1 dead 1 (WN10151, Addgene number: #80441).

Details of target cloning into pWN10042 (pGF-ori-FP vector), and Cpf1 spacer cloning into pTE4396, pTE4398, pTE4495 and pTE4497 can also be found in reference (19). The sequences of the spacers and targets used are listed in Supplementary Table S1 and S2).

Construction of pMbCpf1-crRNA (pTE4495, Addgene number: #80338) and pFnCpf1-crRNA (pTE4497, Addgene number: #80339)

The human U6 promoter and the Mb- or FnCpf1 crRNA was inserted between the *Bcu*I and *Mun*I restriction enzyme sites of pY014 (Addgene number: #69986), or pY004 (Addgene number: #69976), respectively (23) in two steps. First, a human U6-NmCas9 crRNA cassette was inserted (see Sequence section), then the NmCas9 crRNA was replaced by Mb- and FnCpf1 crRNA. The following oligonucleotides were used for MbCpf1 cRNA: #1 CACCGAAATTTCTACTGTTTGTAGATGGAGACGTTCGAACGTCTCCTTTTTT, #2: GTACAAAAAAGGAGACGTTCGAACGTCTCCATCTACAAACAGTAGAAATTTC; and for FnCpf1 cRNA: #1 CACCGTAATTTCTACTGTTGTAGATGGAGACGTTCGAACGTCTCCTTTTTT, #2: GTACAAAAAAGGAGACGTTCGAACGTCTCCATCTACAACAGTAGAAATTAC.

Construction of Cpf1 (Lb, As, Mb, Fn) dead1 and dead2

Each of the vectors harbouring the CDS of the Cpf1 nuclease were amplified in two fragments with overlapping primer pairs at both ends. The overlapping primers at one end of both PCRs contain the desired mutation. The mutations for dead1 and dead2 mutants and the corresponding primers are listed in Supplementary Table S3).

The following inactive Cpf1 constructs were made: Lb-, As-, Fn- and MbCpf1 dead1 and dead2 vectors with crRNA expressing cassette (target 5 and 9) and without crRNA expressing cassette. Some of these constructs are deposited at Addgene [LbCpf1 dead1 without crRNA (pWN10151: #80441, AsCpf1 dead1 without cRNA (pWN10150): #80443, MbCpf1 dead1 without crRNA (pTE4565): #88903, MbCpf1 dead2 without crRNA (pTE4567): #107557, MbCpf1 dead1 with crRNA (pTE4566): #88904, FnCpf1 dead1 without crRNA (pTE4999): #107555, FnCpf1 dead1 with crRNA (pTE4889): #88905].

Construction of Lb crRNA_mCherry, As crRNA_mCherry, Fn crRNA_mCherry, Mb crRNA_mCherry plasmids

The crRNA of As-, Lb-, Fn- and MbCpf1 was inserted between the *Bpi*I and *Acc*65I restriction enzyme sites of pmCherry_gRNA (Addgene No.: #80457). The following oligonucleotides were used for AsCpf1 crRNA: #1 CACCGTAATTTCTACTCTTGTAGATGGAGACGGGATCCCGTCTCCTTTTTT, #2 GTACAAAAAAGGAGACGGGATCCCGTCTCCATCTACAAGAGTAGAAATTAC; for LbCpf1 crRNA: #1 CACCGTAATTTCTACTAAGTGTAGATGGAGACGGGATCCCGTCTCCTTTTTT, #2 GTACAAAAAAGGAGACGGGATCCCGTCTCCATCTACACTTAGTAGAAATTAC; for FnCpf1 crRNA: #1 CACCGTAATTTCTACTGTTGTAGATGGAGACGTTCGAACGTCTCCTTTTTT, #2 GTACAAAAAAGGAGACGTTCGAACGTCTCCATCTACAACAGTAGAAATTAC; for MbCpf1 crRNA: #1 CACCGAAATTTCTACTGTTTGTAGATGGAGACGTTCGAACGTCTCCTTTTTT, #2 GTACAAAAAAGGAGACGTTCGAACGTCTCCATCTACAAACAGTAGAAATTTC. The spacer sequence of these crRNA expressing vectors can be switched by following our spacer cloning protocol (19). The following constructs are deposited at Addgene: Lb crRNA_mCherry (pTE4561) #107525, As crRNA_mCherry (pTE4560) #107526, Fn crRNA_mCherry (pTE4938) #107527, Mb crRNA_mCherry (pTE3179) #107528.

Construction of RVR and RR mutants of different Cpf1 nucleases

Each of the vectors harbouring the CDS of the Cpf1 nuclease were amplified in two fragments with overlapping primer pairs at both ends. The overlapping primers at one end of both PCRs contain the desired mutation. The RR mutants were generated in two consecutive rounds. The mutations for RVR and RR mutants and the corresponding primers are listed in Supplementary Table S5. The following constructs were generated and deposited at Addgene: LbCpf1-RVR without crRNA (pTE3320) #107529, LbCpf1-RVR with crRNA (pTE3330) #107534, FnCpf1-RVR without crRNA (pTE3314) #107530, FnCpf1-RVR with crRNA (pTE3331) #107536, FnCpf1-RR without crRNA (pTE3316) #107532, FnCpf1-RR with crRNA (pTE3333) #107540, MbCpf1-RVR without crRNA (pTE3317) #107531, MbCpf1-RVR with crRNA (pTE3334) #107537, MbCpf1-RR without crRNA (pTE3319) #107533, MbCpf1-RR with crRNA (pTE3336) #107541.

The available As- and LbCpf1 mutants were brought from Addgene [AsCpf1-RVR without crRNA: #89353 (44), AsCpf1-RR without crRNA: #89351 (44), LbCpf1-RR without crRNA: #89355(44)]. The crRNA expression cassette was cloned into these vectors between *Psc*I and *Bcu*I sites from pTE4396 and from pTE4398 respectively. The following constructs were generated and deposited at Addgene: LbCpf1-RR with crRNA (pTE3329) #107538, AsCpf1-RVR with crRNA (pTE3327) #107535, AsCpf1-RR with crRNA (pTE3328) #107539.

SpCas9 spacer cloning

The ligation protocol for Cpf1 spacer cloning (19) was followed except that we used the *Bpi*I restriction enzyme for cloning into px330-U6-Chimeric_BB-CBh-hSpCas9 [Addgene number: #42230 (2)]. The sequences of the spacers used are listed in Supplementary Table S4).

Construction of pHRdonor-EGFP-Dpl1000 and pHRdonor-EGFP-Sho1000

1000nt-long arms homologous to the mouse PRND or SPRN locus were cloned into pEGFP-C1 vector (Clonetech). For its complete sequence please see the sequences below.

**Sequences**

human U6-NmCas9 crRNA cassette between *Bcu*I and *Mun*I restriction enzyme sites

ACTAGTCAATAATCAATGTCAACGCGCCAACTAGCATATCGATATGGATTCGGATCTAAGGTCGGGCAGGAAGAGGGCCTATTTCCCATGATTCCTTCATATTTGCATATACGATACAAGGCTGTTAGAGAGATAATTAGAATTAATTTGACTGTAAACACAAAGATATTAGTACAAAATACGTGACGTAGAAAGTAATAATTTCTTGGGTAGTTTGCAGTTTTAAAATTATGTTTTAAAATGGACTATCATATGCTTACCGTAACTTGAAAGTATTTCGATTTCTTGGCTTTATATATCTTGTGGAAAGGACGAAACACCGGAGACGGGATCCCGTCTCCGTTGTAGCTCCCTTTCTCATTTCGTTTTTTTCGTACGCAATTG

green: *Bcu*I site, blue: human U6 promoter, yellow: NmCas9 crRNA scaffold, pink: RNA polymerase III terminator, grey: *Mun*I site

pHRdonor-EGFP-Dpl1000 plasmid, full sequence

ACATGTGAAGACAACTAACCTGATACCGTGACCCTTTAATACAGTTCTTCATGTTGTGGTGACCCCAACGATAAAGTTATTTCTTTGCTGTTTCATAACTGTAATTTTGCTACTGTCATGAATTGTAATATAATATAAAATATGCAGGACCTTTGATATGTGACCCCCCCCCAAAGGCACCATGACCCACAGGTTGAGAACCACTGCTTTAACCTCTCTGGAAATCCCTCAACAGCCCTGATTTCTATTAGCTAAGGAGCCATCATGCCCCCAGCAACACACACCATTAGCAACACCTGCCCCTGAACTCCTCACAGTCAATGCAACTCAGTTATTGGTGTGTGCTCTCAGAATCAATGTTGAATTCGAATTGGCCACCTAATACATGCAGCTTAGGGCAAATTACTTTGATATCTCTGAATCCCAGGACAAGCTACAAAGATTGGACAAAAGTATTTTCTCCTTGTGAGAATTAGGTCATACCACAAAAAGGTTTTACATAGTATACGGGCTGACATTTAATATGCTATTAGAATGAGCAGAGGGTACATTGTCCCTGGGTGAAACTGGGAAAGTTCCTAACAGCACGTTTTGACAGTGATGTAAGAGACTGGGACCCCTGACTGCATGTCCACTCTCCTTTTCTCCTCCAATTTAGCCTCCTTCTCTCCCTGGTTTCAGAATCAGCAGCCAGTTTCTGGGAGCAGATCTGGTCTGGAACGGTGCCAACTCAGGGCACTAGAAAGTAAGAAAGACTAGGTCAGGCCATTTGATTCCAACTCTTGCCTCCCAGGGATCATTAACTTGAAAGACTGGGAAACACAAGAGAAAGCTGCCTTCCTTATGCATCTGTGTTCCTATGCATGATCAGCAGCTCTGTCCCAGACAACCAAAATCAGACCAAGAGGAGTAACCCCTGGCCAAAACAAAGAAAGCAAGCTGTCAGTGAGTTGACCCACTGCAGCCTGTGCAATAGACATCATTATGATCTGAGGATTCCTTTAAAATCTGTCACTTGTTGGAGGGTGGGAGGCCACCTGAGGAGAGAGTGACAGCCCAGCCTTTCCCTTGCAGATTCACCACACCTGTCTTCATTAATAGTAATCAATTACGGGGTCATTAGTTCATAGCCCATATATGGAGTTCCGCGTTACATAACTTACGGTAAATGGCCCGCCTGGCTGACCGCCCAACGACCCCCGCCCATTGACGTCAATAATGACGTATGTTCCCATAGTAACGCCAATAGGGACTTTCCATTGACGTCAATGGGTGGAGTATTTACGGTAAACTGCCCACTTGGCAGTACATCAAGTGTATCATATGCCAAGTACGCCCCCTATTGACGTCAATGACGGTAAATGGCCCGCCTGGCATTATGCCCAGTACATGACCTTATGGGACTTTCCTACTTGGCAGTACATCTACGTATTAGTCATCGCTATTACCATGGTGATGCGGTTTTGGCAGTACATCAATGGGCGTGGATAGCGGTTTGACTCACGGGGATTTCCAAGTCTCCACCCCATTGACGTCAATGGGAGTTTGTTTTGGCACCAAAATCAACGGGACTTTCCAAAATGTCGTAACAACTCCGCCCCATTGACGCAAATGGGCGGTAGGCGTGTACGGTGGGAGGTCTATATAAGCAGAGCTGGTTTAGTGAACCGTCAGATCCGCTAGCGCTACCGGTCGCCACCATGGTGAGCAAGGGCGAGGAGCTGTTCACCGGGGTGGTGCCCATCCTGGTCGAGCTGGACGGCGACGTAAACGGCCACAAGTTCAGCGTGTCCGGCGAGGGCGAGGGCGATGCCACCTACGGCAAGCTGACCCTGAAGTTCATCTGCACCACCGGCAAGCTGCCCGTGCCCTGGCCCACCCTCGTGACCACCCTGACCTACGGCGTGCAGTGCTTCAGCCGCTACCCCGACCACATGAAGCAGCACGACTTCTTCAAGTCCGCCATGCCCGAAGGCTACGTCCAGGAGCGCACCATCTTCTTCAAGGACGACGGCAACTACAAGACCCGCGCCGAGGTGAAGTTCGAGGGCGACACCCTGGTGAACCGCATCGAGCTGAAGGGCATCGACTTCAAGGAGGACGGCAACATCCTGGGGCACAAGCTGGAGTACAACTACAACAGCCACAACGTCTATATCATGGCCGACAAGCAGAAGAACGGCATCAAGGTGAACTTCAAGATCCGCCACAACATCGAGGACGGCAGCGTGCAGCTCGCCGACCACTACCAGCAGAACACCCCCATCGGCGACGGCCCCGTGCTGCTGCCCGACAACCACTACCTGAGCACCCAGTCCGCCCTGAGCAAAGACCCCAACGAGAAGCGCGATCACATGGTCCTGCTGGAGTTCGTGACCGCCGCCGGGATCACTCTCGGCATGGACGAGCTGTACAAGTAAGGATCCACCGGATCTAGATAACTGATCATAATCAGCCATACCACATTTGTAGAGGTTTTACTTGCTTTAAAAAACCTCCCACACCTCCCCCTGAACCTGAAACATAAAATGAATGCAATTGTTGTTGTTAACTTGTTTATTGCAGCTTATAATGGTTACAAATAAAGCAATAGCATCACAAATTTCACAAATAAAGCATTTTTTTCACTGCATTCTAGTTGTGGTTTGTCCAAACTCATCAATGTATCTTAACGCGTCGTCTCTCCTCCAATGAAGCTGGCAGCCACAGAGGTGAGCTGGTGGGCAAAGGTAGACAGAGGTAGCCCAGTTCTCTCTATCTAGCCCCCGAGTGTTCTGAAAGTACAACGTGTAGCGTTTCAGGGCATTTCAAAAGTCCCTCCCAAGTACTCCCCCTACTCCATGTGTTTGATAATGTGTTTCAGTGCCCCTATGCTCCACCCCTGTGAGACCTGGCCTGTTCCTGCCTTTGCAGCTACACTAGGTGAGAAACCAGCCAAAGGATACAAGAATTGTCCTGTGCACTCCACCTGATTTTCCAACTCCAGGAAACTGAGGCTCACATTGCAGGAGTCCCTGTCCAGCACAGTTAGAAAACAGAAGAGCTGGGGTTCCCCTGCTCTGTCTTCCCTCAGGTTTTCTGCTCTGTGTAGCCTAGAGCTGCCTCCTGATCCCTCCAAGACTCAGGTCCAAGTGCAGTCCCGAATGTGGACTGAAATGTGGAAGTCACACCCTGAACTAAGTGGGAAGCCCTGAGCGACAACCGTGCACCTTGACACCTTACGTGAGACAATATAACAAAAATTAAGCTAAAGTAGACATGACACTAGACAACGGCTCAGAAATCAGATTGTAGGGAAATTTGCTATGCACCTCCCGGAGAGCAGCTTCAGGGCAGGATGTGTTTGTGGTGTCTAGATGCGTCTTCACAAGGACTGGTGTTTGCTGGTTGGGCCCCTGCAATGTCTGCAAAGTTAAGAAAACCTTTTTAAGTGACACATTGTGCGTGCGTGTGCATGCCTGTGTGTGTGTGTGTGTGTGTGTGTGTGTGTTTATTTGTTTTCTAGATAGAAATTTTTCTATGTAGCCCAGGTTGACCCCATAATCATGATCTTCTGGCCTCTGCCTCCTGAATGCTGGGATTGCAGACATGCCACCAAGCCCAATATGTGTTTACTTCTGATGTCCCCTGGCCCTATGTGCATGTGTGTTCTTAGGCATCTTAAGATGCTCACTGCGCACAGAGCTCTCTGCAAACACTGTCTGTTTTTAAAACCCACTTTGGTAAAAGCTAATCTCCGTGAATCATTTCTCGAGACGCACTACGTGAACCATCACCCTAATCAAGTTTTTTGGGGTCGAGGTGCCGTAAAGCACTAAATCGGAACCCTAAAGGGAGCCCCCGATTTAGAGCTTGACGGGGAAAGCCGGCGAACGTGGCGAGAAAGGAAGGGAAGAAAGCGAAAGGAGCGGGCGCTAGGGCGCTGGCAAGTGTAGCGGTCACGCTGCGCGTAACCACCACACCCGCCGCGCTTAATGCGCCGCTACAGGGCGCGTCAGGTGGCACTTTTCGGGGAAATGTGCGCGGAACCCCTATTTGTTTATTTTTCTAAATACATTCAAATATGTATCCGCTCATGAGACAATAACCCTGATAAATGCTTCAATAATATTGAAAAAGGAAGAGTCCTGAGGCGGAAAGAACCAGCTGTGGAATGTGTGTCAGTTAGGGTGTGGAAAGTCCCCAGGCTCCCCAGCAGGCAGAAGTATGCAAAGCATGCATCTCAATTAGTCAGCAACCAGGTGTGGAAAGTCCCCAGGCTCCCCAGCAGGCAGAAGTATGCAAAGCATGCATCTCAATTAGTCAGCAACCATAGTCCCGCCCCTAACTCCGCCCATCCCGCCCCTAACTCCGCCCAGTTCCGCCCATTCTCCGCCCCATGGCTGACTAATTTTTTTTATTTATGCAGAGGCCGAGGCCGCCTCGGCCTCTGAGCTATTCCAGAAGTAGTGAGGAGGCTTTTTTGGAGGCCTAGGCTTTTGCAAAGATCGATCAAGAGACAGGATGAGGATCGTTTCGCATGATTGAACAAGATGGATTGCACGCAGGTTCTCCGGCCGCTTGGGTGGAGAGGCTATTCGGCTATGACTGGGCACAACAGACAATCGGCTGCTCTGATGCCGCCGTGTTCCGGCTGTCAGCGCAGGGGCGCCCGGTTCTTTTTGTCAAGACCGACCTGTCCGGTGCCCTGAATGAACTGCAAGACGAGGCAGCGCGGCTATCGTGGCTGGCCACGACGGGCGTTCCTTGCGCAGCTGTGCTCGACGTTGTCACTGAAGCGGGAAGGGACTGGCTGCTATTGGGCGAAGTGCCGGGGCAGGATCTCCTGTCATCTCACCTTGCTCCTGCCGAGAAAGTATCCATCATGGCTGATGCAATGCGGCGGCTGCATACGCTTGATCCGGCTACCTGCCCATTCGACCACCAAGCGAAACATCGCATCGAGCGAGCACGTACTCGGATGGAAGCCGGTCTTGTCGATCAGGATGATCTGGACGAAGAGCATCAGGGGCTCGCGCCAGCCGAACTGTTCGCCAGGCTCAAGGCGAGCATGCCCGACGGCGAGGATCTCGTCGTGACCCATGGCGATGCCTGCTTGCCGAATATCATGGTGGAAAATGGCCGCTTTTCTGGATTCATCGACTGTGGCCGGCTGGGTGTGGCGGACCGCTATCAGGACATAGCGTTGGCTACCCGTGATATTGCTGAAGAGCTTGGCGGCGAATGGGCTGACCGCTTCCTCGTGCTTTACGGTATCGCCGCTCCCGATTCGCAGCGCATCGCCTTCTATCGCCTTCTTGACGAGTTCTTCTGAGCGGGACTCTGGGGTTCGAAATGACCGACCAAGCGACGCCCAACCTGCCATCACGAGATTTCGATTCCACCGCCGCCTTCTATGAAAGGTTGGGCTTCGGAATCGTTTTCCGGGACGCCGGCTGGATGATCCTCCAGCGCGGGGATCTCATGCTGGAGTTCTTCGCCCACCCTAGGGGGAGGCTAACTGAAACACGGAAGGAGACAATACCGGAAGGAACCCGCGCTATGACGGCAATAAAAAGACAGAATAAAACGCACGGTGTTGGGTCGTTTGTTCATAAACGCGGGGTTCGGTCCCAGGGCTGGCACTCTGTCGATACCCCACCGAGACCCCATTGGGGCCAATACGCCCGCGTTTCTTCCTTTTCCCCACCCCACCCCCCAAGTTCGGGTGAAGGCCCAGGGCTCGCAGCCAACGTCGGGGCGGCAGGCCCTGCCATAGCCTCAGGTTACTCATATATACTTTAGATTGATTTAAAACTTCATTTTTAATTTAAAAGGATCTAGGTGAAGATCCTTTTTGATAATCTCATGACCAAAATCCCTTAACGTGAGTTTTCGTTCCACTGAGCGTCAGACCCCGTAGAAAAGATCAAAGGATCTTCTTGAGATCCTTTTTTTCTGCGCGTAATCTGCTGCTTGCAAACAAAAAAACCACCGCTACCAGCGGTGGTTTGTTTGCCGGATCAAGAGCTACCAACTCTTTTTCCGAAGGTAACTGGCTTCAGCAGAGCGCAGATACCAAATACTGTTCTTCTAGTGTAGCCGTAGTTAGGCCACCACTTCAAGAACTCTGTAGCACCGCCTACATACCTCGCTCTGCTAATCCTGTTACCAGTGGCTGCTGCCAGTGGCGATAAGTCGTGTCTTACCGGGTTGGACTCAAGACGATAGTTACCGGATAAGGCGCAGCGGTCGGGCTGAACGGGGGGTTCGTGCACACAGCCCAGCTTGGAGCGAACGACCTACACCGAACTGAGATACCTACAGCGTGAGCTATGAGAAAGCGCCACGCTTCCCGAAGGGAGAAAGGCGGACAGGTATCCGGTAAGCGGCAGGGTCGGAACAGGAGAGCGCACGAGGGAGCTTCCAGGGGGAAACGCCTGGTATCTTTATAGTCCTGTCGGGTTTCGCCACCTCTGACTTGAGCGTCGATTTTTGTGATGCTCGTCAGGGGGGCGGAGCCTATGGAAAAACGCCAGCAACGCGGCCTTTTTACGGTTCCTGGCCTTTTGCTGGCCTTTTGCTC

yellow: mouse PRND 1000nt homologous arms, grey: CMV promoter, green: GFP coding sequence, pink: polyadenylation signal, red shadow and letters: ampR and SV40 hibrid promoter, blue: aminoglycoside phosphotransferase from Tn5 coding sequence (kanamycin / neomycin resistance), green letters: origin of replication

pHRdonor-EGFP-Sho1000, full sequence

ACATGTGAAGACAACTAACAAAATCCAGTCGTGAGCTCTGCCTAAAGAAAAGGGTCCTCGCTGCCGCACCTTTCCGCTTGGCCGGTCAAGGCCCCTAAATCGCTATCCGACCTAGGCTTGTGACCAGTAAGTAGGGGAGGTGGATGAACCCAGTGATGCTGGGAGGGAGGGGGAGGGGAGAGAGGACCTGAGGAGGATGGAGCTGCCGCCACCGAGATGGCTGGTCCACAGCCAGCCGGAACCCATCCTGATCGGTTTACCTGTCCAGGATACTGCCCTGGACAAACCCAAAAAGGGTGGAGCTGGAGCGGGGAAGAGACGTAATTACCTAGGGTCTGGGCCTGCTGGGCGGTTCACCCATCGCTAGTTGTTTGCGTTGCAAGGAATCGCTGATCTGGATTCTGACCCCCACCCTCACCCAGTGCATTCAGCCGCAGCCACTGGCTGAAAGACTATCTCTTAAGGCATCAGGAGATCCAGATGCCAGAGCAAAAGTCACAAGGCTGTCCATTCTAACCATAATCTGGGGGTATTGAAGGCTCTCCATTCCAAACTAGAATCCTAATCCACTAATCCATCCCCTTCCAGAGACTCGTGTGCAGAGCGGGGGATTTGTGCCCCCCCCCCAGGCCTGAGCCCCACTGTAGGAGCTCCGCAAACCCCATTCTGGGACCCATCTCCACCCTATCACAATAGTAAAACGGCCGCCCTGTAGTTAAACCCTTTCCCCACCACCCCATCTCCACCTGGTTATACAGCAGGAACCCAAAACCAAAGTCCTGGTGCTGGAGTTTAAAGAACCCCTTCCCCAACCCTGAGCCCACTGTCCTCCAAGATGCTGGGAGCCCTCTATCGGATTACCACAAAACCAGAGGCTGAAGTAGGTGTCCCCAGGTCCAGATGAGCCTATTCCCAAGCCCTGATACCCTCTTGCCCTGGTCCTAAACCACGCTCCACCCCTGCACAGAAGCTGAAGCCCCTTCCACCCTCTTCTCGCAGATTCTGCCCAGTAGGACACCTGTCTTCATTAATAGTAATCAATTACGGGGTCATTAGTTCATAGCCCATATATGGAGTTCCGCGTTACATAACTTACGGTAAATGGCCCGCCTGGCTGACCGCCCAACGACCCCCGCCCATTGACGTCAATAATGACGTATGTTCCCATAGTAACGCCAATAGGGACTTTCCATTGACGTCAATGGGTGGAGTATTTACGGTAAACTGCCCACTTGGCAGTACATCAAGTGTATCATATGCCAAGTACGCCCCCTATTGACGTCAATGACGGTAAATGGCCCGCCTGGCATTATGCCCAGTACATGACCTTATGGGACTTTCCTACTTGGCAGTACATCTACGTATTAGTCATCGCTATTACCATGGTGATGCGGTTTTGGCAGTACATCAATGGGCGTGGATAGCGGTTTGACTCACGGGGATTTCCAAGTCTCCACCCCATTGACGTCAATGGGAGTTTGTTTTGGCACCAAAATCAACGGGACTTTCCAAAATGTCGTAACAACTCCGCCCCATTGACGCAAATGGGCGGTAGGCGTGTACGGTGGGAGGTCTATATAAGCAGAGCTGGTTTAGTGAACCGTCAGATCCGCTAGCGCTACCGGTCGCCACCATGGTGAGCAAGGGCGAGGAGCTGTTCACCGGGGTGGTGCCCATCCTGGTCGAGCTGGACGGCGACGTAAACGGCCACAAGTTCAGCGTGTCCGGCGAGGGCGAGGGCGATGCCACCTACGGCAAGCTGACCCTGAAGTTCATCTGCACCACCGGCAAGCTGCCCGTGCCCTGGCCCACCCTCGTGACCACCCTGACCTACGGCGTGCAGTGCTTCAGCCGCTACCCCGACCACATGAAGCAGCACGACTTCTTCAAGTCCGCCATGCCCGAAGGCTACGTCCAGGAGCGCACCATCTTCTTCAAGGACGACGGCAACTACAAGACCCGCGCCGAGGTGAAGTTCGAGGGCGACACCCTGGTGAACCGCATCGAGCTGAAGGGCATCGACTTCAAGGAGGACGGCAACATCCTGGGGCACAAGCTGGAGTACAACTACAACAGCCACAACGTCTATATCATGGCCGACAAGCAGAAGAACGGCATCAAGGTGAACTTCAAGATCCGCCACAACATCGAGGACGGCAGCGTGCAGCTCGCCGACCACTACCAGCAGAACACCCCCATCGGCGACGGCCCCGTGCTGCTGCCCGACAACCACTACCTGAGCACCCAGTCCGCCCTGAGCAAAGACCCCAACGAGAAGCGCGATCACATGGTCCTGCTGGAGTTCGTGACCGCCGCCGGGATCACTCTCGGCATGGACGAGCTGTACAAGTCCGGACTCAGATCTCGAGCTCAAGCTTCGAATTCTGCAGTCGACGGTACCGCGGGCCCGGGATCCACCGGATCTAGATAACTGATCATAATCAGCCATACCACATTTGTAGAGGTTTTACTTGCTTTAAAAAACCTCCCACACCTCCCCCTGAACCTGAAACATAAAATGAATGCAATTGTTGTTGTTAACTTGTTTATTGCAGCTTATAATGGTTACAAATAAAGCAATAGCATCACAAATTTCACAAATAAAGCATTTTTTTCACTGCATTCTAGTTGTGGTTTGTCCAAACTCATCAATGTATCTTAACGCGTCGTCTCTCCTCACCAGGCTAAACTCCATCCCAGGTCTAGCTCCTAGCCTGTCTTAAGGCCCCTAGGGCCCACCCTAATGGCCTCCTGCCCAGGGGGTAACTTCATTTTGCTAACTATGATTCCCCAGCCCAGAGAAGAGTCTGGCCATTCTGGGCCCACAAGGCCTCTTGCAATTTGTGGATGTAGTTTACTCTCTTGCCCATCCCAGTTTCCCAGTCTTCCACTCTGGCAGGAACTAACAGCCCTACTACCAGAGAGGCAGGCTGCCCTTGACTCTCCAGAACTGCCCAAGCAGGCATGCCTGTCTGCTTGCCCACCCTGACACGAGGACATGAACAATGCTGACCGCCAAGAAGAGGCCATCCTTGGGTGGGCCCGTCTGATTCTGCCACTAAAGCCCCCAAAGACCTTGAGCCTCCCATGGAACCAGAGGGACTTATAGTTGATGAAGAGAGCATTCACTTAGACTGCAGTGTGAGAAATGTCAGGTGCTAACCACCTGCCCAGAGCAGGTCCCAATGATGTCATAGCTCAGAAAACATCCAGAGCAGTAAAATAACCATCCCAGGACGCCCACCATGCTCCTCCTACAACATGCTACCAAAGCCAAGTAGTATGTTTCTTCCTGGCAGACTGCCTAAGAGACCTTGTGTCAGAAGGGTTTCCACTTGAAGCCACTTGGTCCTAAAATCCACTGAGGGTAGAGGTTTTGAATACACTTTCAAAAACATTCCATTCTGCTTGAGCTTAAGGGTCATGAGTGAGGGTCACTTGGATATAATACCAATCCTGCTGGGGCCTTCTTTGTATATAACCCAAACTGCAAGATTCCCATAGTTCCAGTAGATAGCAGCATTTTATGTTGGGAGACCCCTCCCTTGGAAACGGTTGGACGGGGTTGGGGTGGGGAGGTGAGACAGAGCATGGCACAGCTGACAGCTGGCAAACTGAACAGTGGAAGGGGCAGCAGATCTACAGCCCCACTGTGCCAGAACAAAGCTAGCAGACAGATTCTCGAGACGCACTACGTGAACCATCACCCTAATCAAGTTTTTTGGGGTCGAGGTGCCGTAAAGCACTAAATCGGAACCCTAAAGGGAGCCCCCGATTTAGAGCTTGACGGGGAAAGCCGGCGAACGTGGCGAGAAAGGAAGGGAAGAAAGCGAAAGGAGCGGGCGCTAGGGCGCTGGCAAGTGTAGCGGTCACGCTGCGCGTAACCACCACACCCGCCGCGCTTAATGCGCCGCTACAGGGCGCGTCAGGTGGCACTTTTCGGGGAAATGTGCGCGGAACCCCTATTTGTTTATTTTTCTAAATACATTCAAATATGTATCCGCTCATGAGACAATAACCCTGATAAATGCTTCAATAATATTGAAAAAGGAAGAGTCCTGAGGCGGAAAGAACCAGCTGTGGAATGTGTGTCAGTTAGGGTGTGGAAAGTCCCCAGGCTCCCCAGCAGGCAGAAGTATGCAAAGCATGCATCTCAATTAGTCAGCAACCAGGTGTGGAAAGTCCCCAGGCTCCCCAGCAGGCAGAAGTATGCAAAGCATGCATCTCAATTAGTCAGCAACCATAGTCCCGCCCCTAACTCCGCCCATCCCGCCCCTAACTCCGCCCAGTTCCGCCCATTCTCCGCCCCATGGCTGACTAATTTTTTTTATTTATGCAGAGGCCGAGGCCGCCTCGGCCTCTGAGCTATTCCAGAAGTAGTGAGGAGGCTTTTTTGGAGGCCTAGGCTTTTGCAAAGATCGATCAAGAGACAGGATGAGGATCGTTTCGCATGATTGAACAAGATGGATTGCACGCAGGTTCTCCGGCCGCTTGGGTGGAGAGGCTATTCGGCTATGACTGGGCACAACAGACAATCGGCTGCTCTGATGCCGCCGTGTTCCGGCTGTCAGCGCAGGGGCGCCCGGTTCTTTTTGTCAAGACCGACCTGTCCGGTGCCCTGAATGAACTGCAAGACGAGGCAGCGCGGCTATCGTGGCTGGCCACGACGGGCGTTCCTTGCGCAGCTGTGCTCGACGTTGTCACTGAAGCGGGAAGGGACTGGCTGCTATTGGGCGAAGTGCCGGGGCAGGATCTCCTGTCATCTCACCTTGCTCCTGCCGAGAAAGTATCCATCATGGCTGATGCAATGCGGCGGCTGCATACGCTTGATCCGGCTACCTGCCCATTCGACCACCAAGCGAAACATCGCATCGAGCGAGCACGTACTCGGATGGAAGCCGGTCTTGTCGATCAGGATGATCTGGACGAAGAGCATCAGGGGCTCGCGCCAGCCGAACTGTTCGCCAGGCTCAAGGCGAGCATGCCCGACGGCGAGGATCTCGTCGTGACCCATGGCGATGCCTGCTTGCCGAATATCATGGTGGAAAATGGCCGCTTTTCTGGATTCATCGACTGTGGCCGGCTGGGTGTGGCGGACCGCTATCAGGACATAGCGTTGGCTACCCGTGATATTGCTGAAGAGCTTGGCGGCGAATGGGCTGACCGCTTCCTCGTGCTTTACGGTATCGCCGCTCCCGATTCGCAGCGCATCGCCTTCTATCGCCTTCTTGACGAGTTCTTCTGAGCGGGACTCTGGGGTTCGAAATGACCGACCAAGCGACGCCCAACCTGCCATCACGAGATTTCGATTCCACCGCCGCCTTCTATGAAAGGTTGGGCTTCGGAATCGTTTTCCGGGACGCCGGCTGGATGATCCTCCAGCGCGGGGATCTCATGCTGGAGTTCTTCGCCCACCCTAGGGGGAGGCTAACTGAAACACGGAAGGAGACAATACCGGAAGGAACCCGCGCTATGACGGCAATAAAAAGACAGAATAAAACGCACGGTGTTGGGTCGTTTGTTCATAAACGCGGGGTTCGGTCCCAGGGCTGGCACTCTGTCGATACCCCACCGAGACCCCATTGGGGCCAATACGCCCGCGTTTCTTCCTTTTCCCCACCCCACCCCCCAAGTTCGGGTGAAGGCCCAGGGCTCGCAGCCAACGTCGGGGCGGCAGGCCCTGCCATAGCCTCAGGTTACTCATATATACTTTAGATTGATTTAAAACTTCATTTTTAATTTAAAAGGATCTAGGTGAAGATCCTTTTTGATAATCTCATGACCAAAATCCCTTAACGTGAGTTTTCGTTCCACTGAGCGTCAGACCCCGTAGAAAAGATCAAAGGATCTTCTTGAGATCCTTTTTTTCTGCGCGTAATCTGCTGCTTGCAAACAAAAAAACCACCGCTACCAGCGGTGGTTTGTTTGCCGGATCAAGAGCTACCAACTCTTTTTCCGAAGGTAACTGGCTTCAGCAGAGCGCAGATACCAAATACTGTTCTTCTAGTGTAGCCGTAGTTAGGCCACCACTTCAAGAACTCTGTAGCACCGCCTACATACCTCGCTCTGCTAATCCTGTTACCAGTGGCTGCTGCCAGTGGCGATAAGTCGTGTCTTACCGGGTTGGACTCAAGACGATAGTTACCGGATAAGGCGCAGCGGTCGGGCTGAACGGGGGGTTCGTGCACACAGCCCAGCTTGGAGCGAACGACCTACACCGAACTGAGATACCTACAGCGTGAGCTATGAGAAAGCGCCACGCTTCCCGAAGGGAGAAAGGCGGACAGGTATCCGGTAAGCGGCAGGGTCGGAACAGGAGAGCGCACGAGGGAGCTTCCAGGGGGAAACGCCTGGTATCTTTATAGTCCTGTCGGGTTTCGCCACCTCTGACTTGAGCGTCGATTTTTGTGATGCTCGTCAGGGGGGCGGAGCCTATGGAAAAACGCCAGCAACGCGGCCTTTTTACGGTTCCTGGCCTTTTGCTGGCCTTTTGCTC

yellow: mouse SPRN 1000nt homologous arms, grey: CMV promoter, green: GFP coding sequence, pink: polyadenylation signal, red shadow and letters: ampR and SV40 hibrid promoter, blue: aminoglycoside phosphotransferase from Tn5 coding sequence (kanamycin / neomycin resistance), green letters: origin of replication

**Supplementary References**

(2) Cong, L, Ran, FA, Cox, D, Lin, S, Barretto, R, Habib, N*, et al.* (2013). Multiplex genome engineering using CRISPR/Cas systems. *Science* **339**: 819-823.

(19) Toth, E, Weinhardt, N, Bencsura, P, Huszar, K, Kulcsar, PI, Talas, A*, et al.* (2016). Cpf1 nucleases demonstrate robust activity to induce DNA modification by exploiting homology directed repair pathways in mammalian cells. *Biol Direct* **11**: 46.

(23) Zetsche, B, Gootenberg, JS, Abudayyeh, OO, Slaymaker, IM, Makarova, KS, Essletzbichler, P*, et al.* (2015). Cpf1 is a single RNA-guided endonuclease of a class 2 CRISPR-Cas system. *Cell* **163**: 759-771.

(44) Gao, L., Cox, D.B.T., Yan, W.X., Manteiga, J.C., Schneider, M.W., Yamano, T., Nishimasu, H., Nureki, O., Crosetto, N. and Zhang, F. (2017) Engineered Cpf1 variants with altered PAM specificities. *Nat Biotechnol*, **35**, 789-792.
